# Curved Nanographenes as Stoppers in a [2]Rotaxane with Two-Photon Excited Emission

**DOI:** 10.1021/acs.joc.4c00486

**Published:** 2024-06-22

**Authors:** Marcos D. Codesal, Arthur H. G. David, Carla I. M. Santos, Maria J. Álvaro-Martins, Ermelinda Maçôas, Araceli G. Campaña, Victor Blanco

**Affiliations:** † Departamento de Química Orgánica, Unidad de Excelencia de Química, Facultad de Ciencias, Universidad de Granada, Avda. Fuente Nueva s/n, 18071 Granada, Spain; ‡ Centro de Química Estrutural and Institute of Molecular Sciences, Instituto Superior Técnico, Universidade de Lisboa, Av. Rovisco Pais, 1, 1049-001 Lisboa, Portugal

## Abstract

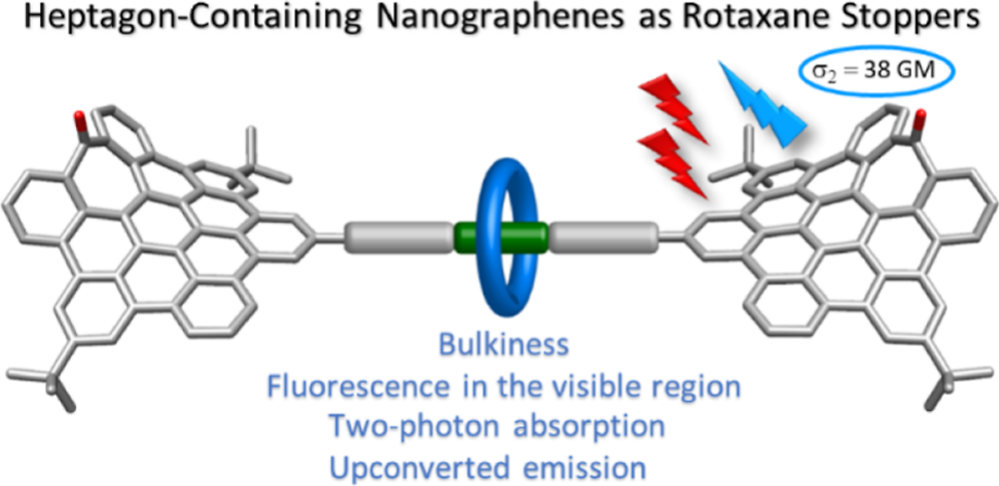

Heptagon-containing distorted nanographenes are used as stoppers for the capping of a [2]rotaxane through a Michael-type addition reaction to vinyl sulfone groups. These curved aromatics are bulky enough to prevent the disassembly of the rotaxane but also give emissive and nonlinear (two-photon absorption and emission) optical properties to the structure.

## Introduction

Over the years, most of the research carried out in the field of rotaxanes has been focused on the search of new synthetic strategies to access such interlocked structures and on the development of molecular devices or machines based on them.^1,2^ Since the introduction of the template effect in rotaxane synthesis,^3^ there has been an impressive advance on the structural motifs that can be placed in the thread or the macrocycle (or their precursors) to preorganize the components through a variety of noncovalent interactions.^1,4^ In the same way, many reactions have been investigated for thread capping or ring closing in the synthesis of interlocked structures.^1,4^

Initially, the development of synthetic strategies toward rotaxane architectures did not pay much attention to the nature of the stoppers, the bulky groups attached to both ends of the linear component to prevent the disassembly of the system. In fact, most examples of reported rotaxanes displayed arene (e.g., trityl-based stoppers) or alkane (e.g., functionalized with *t*-butyl groups) motifs or combinations of them as stoppers, with no properties or functions other than their bulkiness.^1,4^ There are, however, exceptions, and some early designs already incorporated stopper units with different properties that could play a functional role such as fullerenes^5^ or porphyrins,^6^ which have continued to be used over the years.^7^ Since then, an increasingly amount of potentially functional structures such as peptides or proteins,^8^ nanoparticles or inorganic clusters,^9^ oligonucleotides,^10^ subpthalocyanines,^11^ drugs,^12^ cyclodextrins or calixarenes,^13^ ligands,^14^ redox or photo-active groups,^15^ or radicals^16^ have been progressively incorporated as stopper units in rotaxanes. In this sense, we can highlight functional stoppers with luminescence properties.^17^ For instance, the anthracene core has been used as fluorescent stopper in rotaxanes, and its emission has been modulated in different stimuli-responsive molecular machines.^17a,17d,17e^ However, to the best of our knowledge, there are no examples reported of nanographenes only as rotaxane stoppers.^18^

In recent years, our group has developed new synthetic strategies for the synthesis of distorted hexa-*peri*-hexabenzocoronene (HBC) derivatives containing a nonhexagonal ring,^19^ mainly a cycloheptatrienone moiety.^19a^ The introduction of a heptagonal ring leads to a saddle-shaped nanographene with much better solubility in organic solvents than that of their planar counterparts. The heptagon-containing HBC analogues (*hept*-HBC) were incorporated into a variety of structures, resulting in nanographenes with remarkable optical properties.^19a,20^ In particular, these nanographenes are fluorescent, with high emission quantum yields in comparison with those of other polycyclic aromatic hydrocarbons (PAHs), and they emit at longer wavelengths (λ_em_) when embedded into π-extended systems. Moreover, many display nonlinear optical properties,^20b,20c^ namely, two-photon absorption (TPA) and two-photon emission (TPE), in which the heptagonal ring has been proven to have a positive effect.^20c^*Hept*-HBCs were also embedded in chiral structures, giving rise to chiroptical properties such as circularly polarized luminescence.^20a,20b^

Hence, the high bulkiness, good solubility, and these interesting optical properties have prompted us to study the use of a saddle-shaped *hept*-HBC as a stopper in rotaxane architectures. Within this context, here, we report the synthesis through a threading-and-capping strategy of a [2]rotaxane using distorted *hept*-HBC derivatives as stoppers. These *hept*-HBC units act as functional stoppers because they are bulky enough to prevent the dethreading of the macrocycle while also giving the rotaxane new linear and nonlinear optical properties (TPA and TPE).

The study of the nonlinear absorption/emission processes is especially relevant since it can have practical implications in the precision with which systems are actuated by light. Nevertheless, the nonlinear absorption/emission processes in rotaxane architectures have been clearly overlooked^21^ with respect to linear optical properties and even other nonlinear optical phenomena, such as the second- and third-order harmonic generation and the optical Kerr effect.^22^ To the best of our knowledge, there is only a single example reported of a [2]rotaxane with two-photon-excited fluorescence properties.^21^ In that example, the interlocked architecture is selected to enhance the stability of a squaraine recognition motif acting as the nonlinear fluorophore, and therefore, this approach is restricted to that specific recognition unit. The inclusion of robust heptagon-containing nanographenes as versatile stoppers could allow for a near-infrared (NIR)-excited emission in [2]rotaxanes with a wide variety of motifs both in the thread and the macrocycle through a variety of noncovalent interactions. New linear and nonlinear optical properties could then be further explored in light responsive molecular machines and optical switching elements.

## Results and Discussion

### Design

**Scheme 1:**
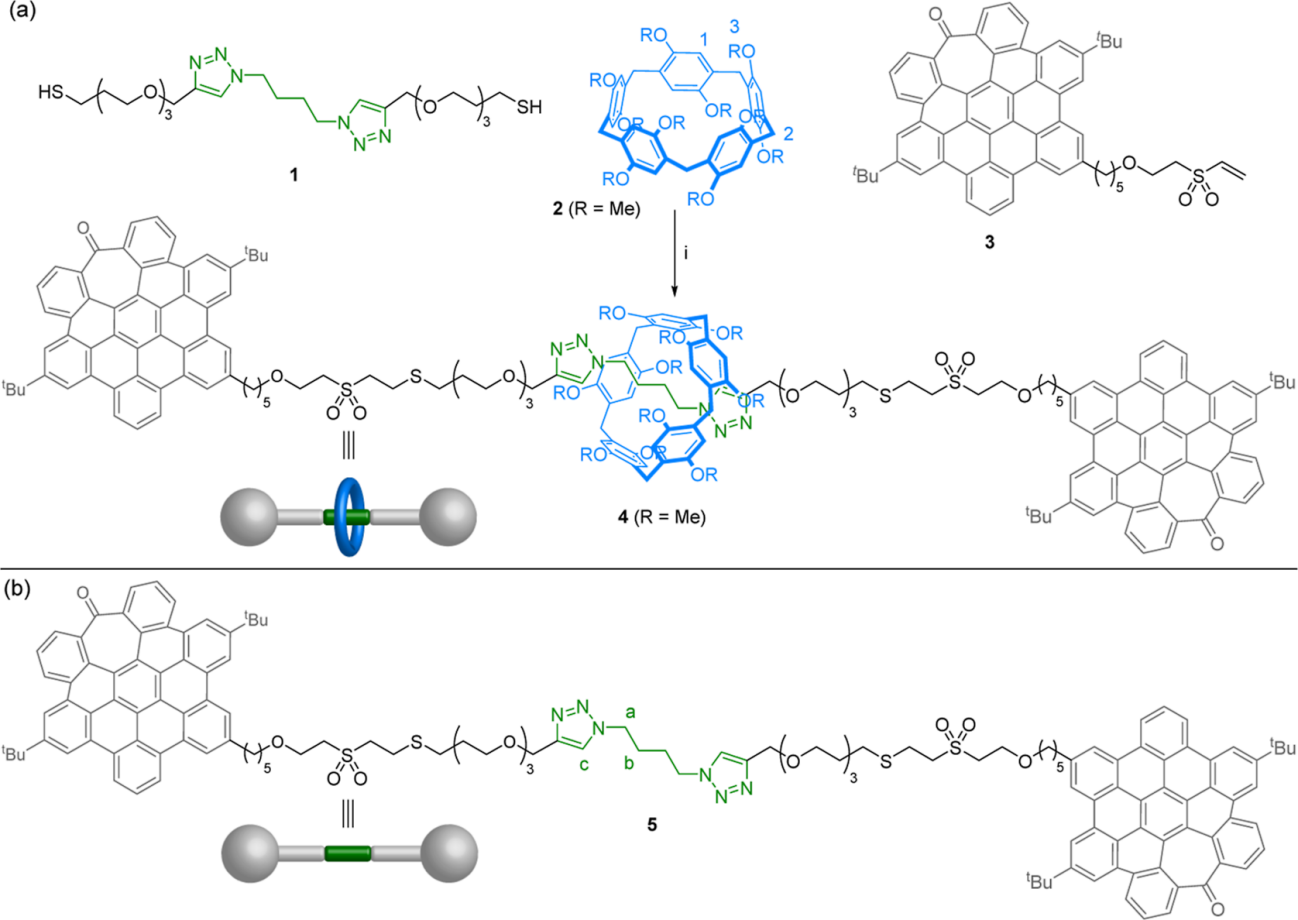
(a) Structure of Rotaxane **4** with Two *hept*-HBC Units as Stoppers and Synthesis from Its Corresponding Building Blocks and (b) Structure of Thread **5** Reagents and conditions: (i) PPh_3_, Et_3_N, CHCl_3_, r.t., 24 h, 40%.

The system in which we contemplated to study the use of *hept*-HBC derivatives as stoppers is [2]rotaxane **4** (Scheme 1a) incorporating a *per*-*O*-methyl-pillar[5]arene as the macrocycle and 1,4-di(1,2,3-triazol-1-yl)-butane as the recognition motif on the thread. This host–guest system displays a good binding affinity in chlorinated solvents [*K*_a_ = (1.6 ± 0.3) × 10^4^ M^–1^ at 298 K in CDCl_3_ using *per*-*O*-ethyl-pillar[5]arene]^23^ and has been previously used in the development of [2]rotaxanes.^24^

As the reaction for the capping step, we chose the Michael-type addition reaction to the vinyl sulfonyl group.^25^ As we previously demonstrated, this reaction has been shown to be efficient and versatile in the synthesis of rotaxanes,^24,26^ including examples based on pillar[5]arene and the binding unit proposed here.^24^ In order to apply this strategy, we designed a *hept*-HBC derivative (compound **3**) bearing a vinyl sulfone group attached to the aromatic core through an aliphatic linker. The thread precursor consists of the ditriazolyl butane core functionalized with alkyl chains bearing diethylene glycol units and terminal thiol groups (**1**), which can act as nucleophiles in the thia-Michael addition to the stopper vinyl sulfone (Scheme 1a). In this work, we aim to demonstrate a general strategy to introduce new properties that are additional or even orthogonal to those of the rotaxane. Therefore, the proposed structure is designed so that the binding site and the nanographene stoppers are far away from each other to ensure that the optical response of the *hept*-HBC is not altered in the final interlocked structure.

**Figure 1:**
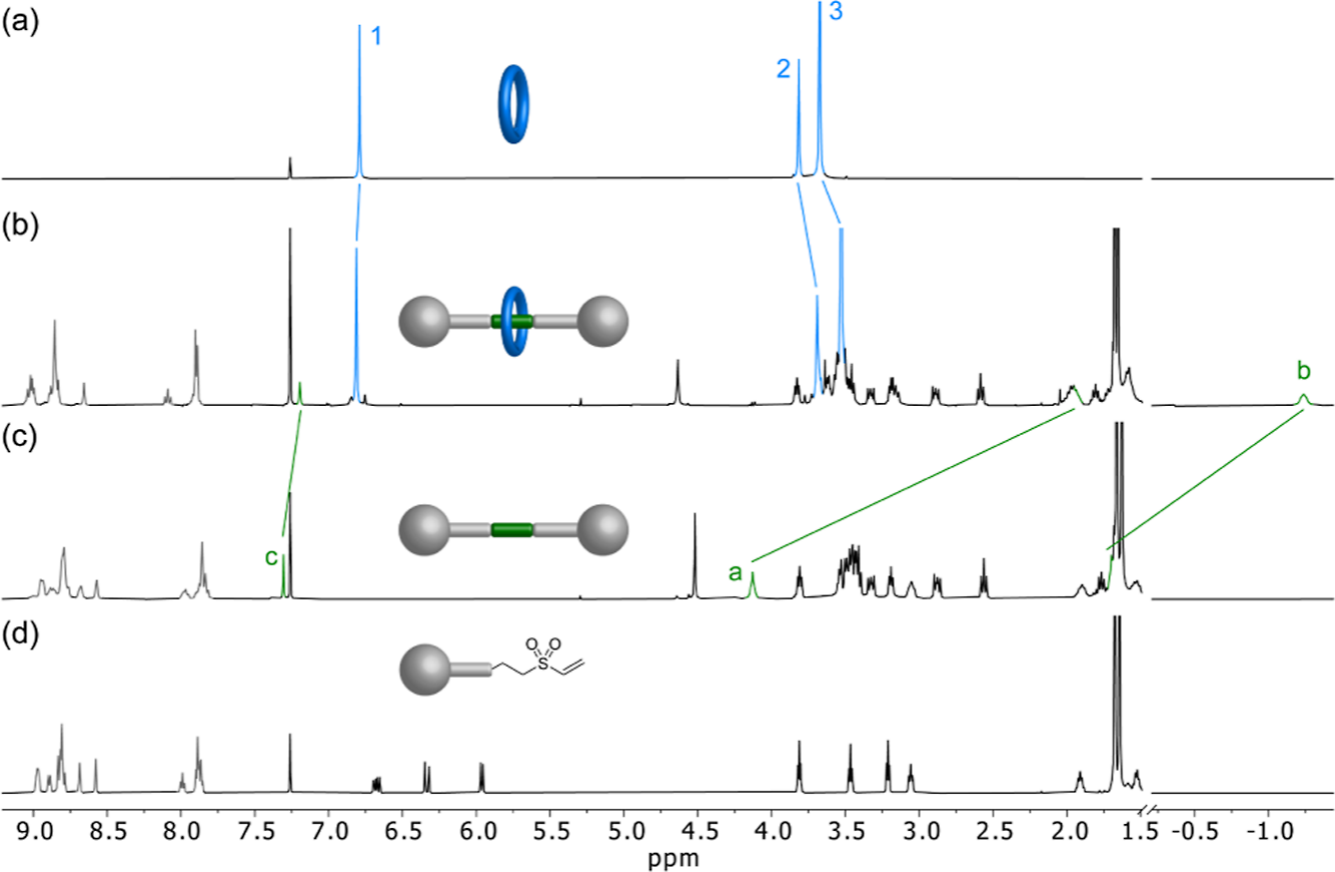
^1^H NMR (CDCl_3_) spectra of (a) Macrocycle **2** (400 MHz); (b) rotaxane **4** (400 MHz); (c) thread **5** (400 MHz); and (d) vinyl sulfone stopper **3** (600 MHz). The assignment and color coding of the signals correspond to those shown in Scheme 1.

### Synthesis and Characterization

**Scheme 2:**
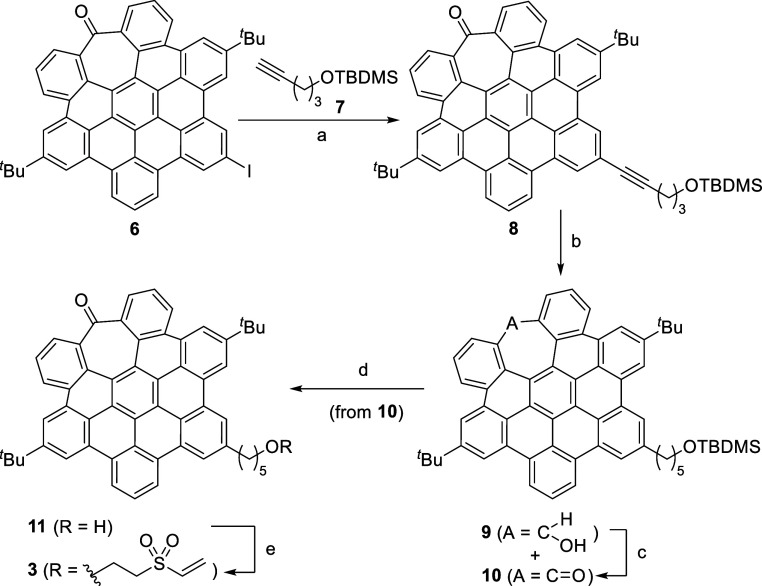
Synthesis of Vinyl Sulfone *hept*-HBC-Based Stopper **3** Reagents and conditions: (a) Pd(PPh_3_)_2_Cl_2_, CuI, Et_3_N, THF, r.t., 20 h, 92%; (b) H_2_, PtO_2_, THF/MeOH, r.t., 24 h; (c) Dess–Martin periodinane, CH_2_Cl_2_, 0 °C to r.t., 24 h, (73% over two steps); (d) TBAF, THF, r.t., 2 h, 63%; (e) divinyl sulfone, ^*t*^BuOK, THF, r.t., 55 min, 53%.

The synthesis of the stopper starts with the iodine-functionalized *hept*-HBC derivative **6** (Scheme 2) previously prepared following the synthetic methodology developed by our group.^19a^ This compound bears an aryl-iodide that enabled a Sonogashira coupling with TBDMS-protected 4-pentyl-1-ol (compound **7**) to afford **8** in 92% yield. Hydrogenation of the resulting alkyne with H_2_/PtO_2_ led to a mixture of compounds **9** and **10**, which after treatment with Dess–Martin periodinane afforded compound **10** in 73% yield over two steps. Removal of the TBDMS group with TBAF gave alcohol **11** (63% yield), which allowed us to introduce the vinyl sulfone group by the reaction with divinyl sulfone in the presence of ^*t*^BuOK as a base, obtaining the target *hept*-HBC derivative **3** with the suitable Michael acceptor vinyl sulfone group in moderate yield (53%).

**Scheme 3:**
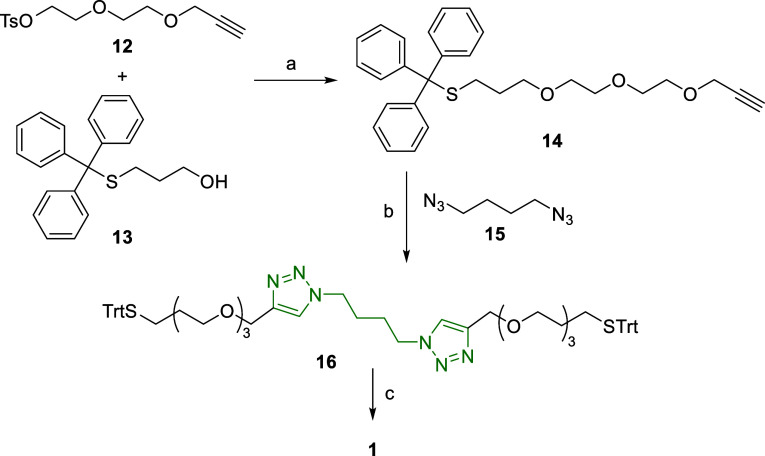
Synthesis of the Thiol-Functionalized Thread Precursor **1** Trt = trityl. Reagents and conditions: (a) NaH, ^*n*^Bu_4_NI, THF, 0 °C to reflux, 24 h, 55%; (b) Cu(CH_3_CN)_4_PF_6_, TBTA, CH_2_Cl_2_, r.t., 24 h, 94%; (c) CF_3_CO_2_H, Et_3_SiH, CH_2_Cl_2_, r.t., 5 h, 98%.

The strategy to obtain the linear component followed a convergent route in which the 1,4-di(1,2,3-triazol-1-yl)-butane was formed in the final steps of the synthesis (Scheme 3). Thus, we first prepared compound **14** by the reaction of the tosylated monopropargyl diethylene glycol **12** with trityl-protected 3-mercaptopropan-1-ol (**13**, see Scheme S1 for its synthesis). Compound **14** exhibits on one end a protected thiol, which would enable the capping step with the stopper, and a terminal alkyne, required to build the 1,2,3-triazole moieties in the recognition motif. Thus, the Cu-catalyzed azide–alkyne cycloaddition (CuAAC) reaction of **14** with 1,4-diazidobutane (**15**) afforded compound **16**, which already displays the 1,4-di(1,2,3-triazol-1-yl)-butane recognition motif, in 94% yield. Finally, removal of the trityl protecting groups quantitatively afforded the thread precursor **1**, ready for its use in rotaxane formation.

With all components in hand, we tackled the synthesis of both the [2]rotaxane and the free thread. In the absence of the macrocycle, the double thia-Michael addition of the thiol groups of precursor **1** to the vinyl sulfone moiety of *hept*-HBC stopper **3** led to the corresponding free thread **5** in 50% yield (see the Supporting Information for details). In the same way, when the reaction was carried out with the pseudorotaxane, formed by supramolecular assembly of **1** and *per*-*O*-methyl-pillar[5]arene (**2**) (Figure S1), the target [2]rotaxane **4** was obtained in 40% yield (Scheme 1).

[2]rotaxane **4** and free thread **5** were characterized by means of 1D and 2D NMR spectroscopy. In the ^1^H NMR spectrum of the [2]rotaxane, we can observe signals corresponding to the three components, *hept*-HBC stoppers, macrocycle, and recognition motif (Figure 1b). There is also a drastic shift toward lower frequencies of the aliphatic signals of the ditriazolyl binding site (*H*_a_: δ = 1.94 ppm, Δδ = −2.19 ppm; H_b_: δ = −1.24 ppm, Δδ = −2.94 ppm) in comparison with the free thread. These are the typical changes in the chemical shift reported for this recognition motif in mechanically interlocked molecules based on its interaction with pillar[5]arenes.^23,24^ This shift is due to the shielding effect of the macrocycle aromatic rings upon inclusion of the linear triazolyl butane moiety into its cavity (Figure 1). DOSY NMR experiments also support the interlocked nature of the structure formed as the different signals of both components have the same diffusion coefficient (*D* = 3.2 × 10^–10^ m^2^ s^–1^), showing that the compound diffuses as a single species (Figure S57). High-resolution mass spectrometry exact mass and isotopic distribution confirmed the identity of the [2]rotaxane (Figures S67–S68).

In addition, we have also studied the system in DMSO-*d*_6_. In this solvent, the macrocycle shows a negligible interaction with the binding site, as can be observed from the ^1^H NMR spectrum of the mixture of **1** and **2**, which does not show any significant shifts of the signals of the recognition motif (Figure S2). Therefore, the analysis of the spectrum of **4** in DMSO-*d*_6_ can provide valuable information to further validate both that the *hept*-HBC core has the appropriate size and is bulky enough to act as a true stopper and the interlocked nature of the [2]rotaxane. In this sense, we considered the signal of *H*_b_ as the key diagnostic signal since it appears below −1.0 ppm, a region usually with no signals and, therefore, easy to analyze. This signal could be clearly identified in the ^1^H NMR spectrum of **4** in DMSO-*d*_6_, thus confirming that the linear component is threaded through the cavity of the macrocycle, without the possibility of disassembly due to the bulkiness of the *hept*-HBC-based stopper (Figure S3).

### Optical Properties

**Figure 2:**
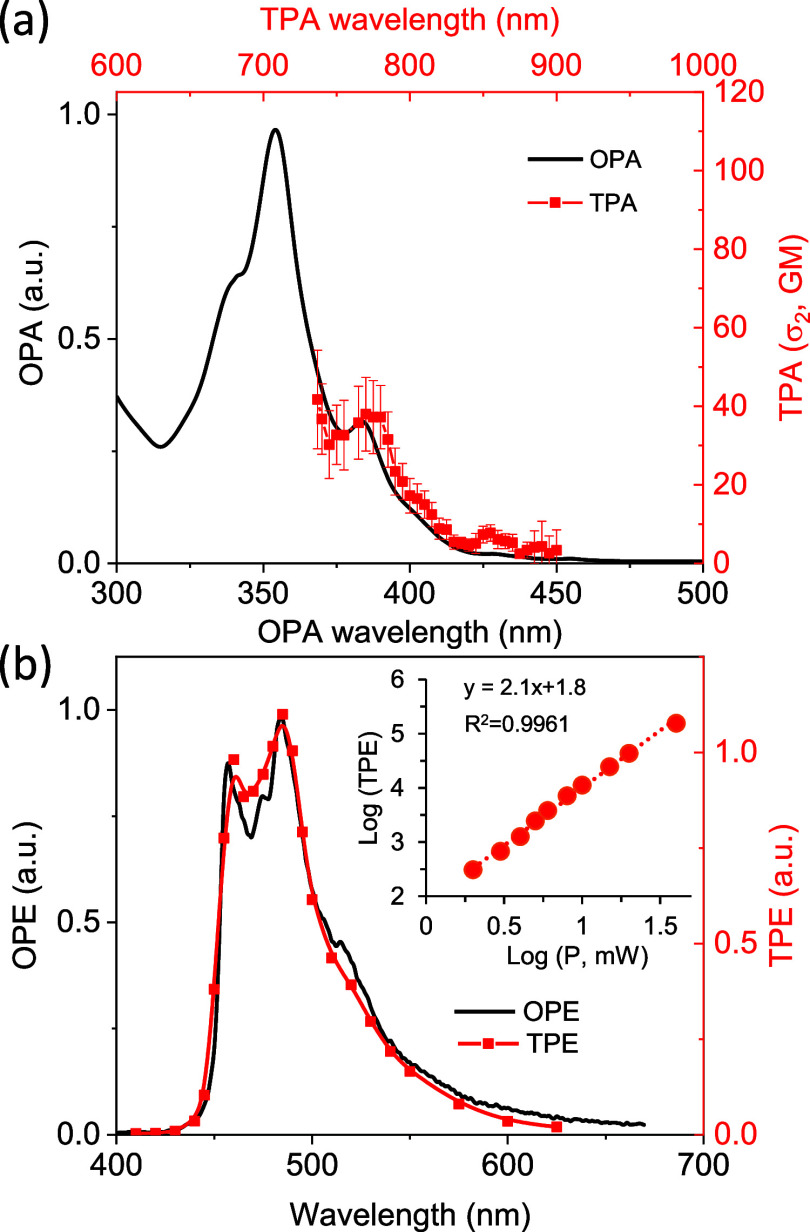
(a) Absorbance (OPA, black line, 10 μM) and TPA (red line, 14 μM) spectra of [2]rotaxane **4** in CH_2_Cl_2_ and (b) fluorescence (OPE, black line, λ_exc_ = 354 nm) and two-photon induced emission (TPE, red line, λ_exc_ = 785 nm) spectra of **4**. Inset: log–log plot of the TPE intensity against the power of the excitation source.

The use of the *hept*-HBC derivatives as stoppers affords a [2]rotaxane architecture that exhibits linear and nonlinear optical responses similar to those reported earlier for distorted nanographenes. The linear and nonlinear absorption and emission of [2]rotaxane **4** was investigated in CH_2_Cl_2_. The UV–vis spectrum shows absorption in the 315–440 nm region, peaking at 354 nm (ε = 1.7 × 10^5^ M^–1^ cm^–1^) with shoulders at 341 and 384 nm (Figure 2a). The structured absorption is due to strongly allowed π–π* transitions within the *hept*-HBC core. The assignment is supported by the calculated transitions at the CAM-B3LYP/def2TZVP level (Table S3). In addition, the contribution of vibronic progressions to the structure of the main band cannot be excluded. As typically observed in PAHs,^27^ the absorption spectrum shows a red edge, extending up to 500 nm, due to several weakly allowed π–π* transitions (Figures 2 and S79). The relative position and intensity of the time-dependent density functional theory computed transitions are in good agreement with the observed spectrum (Figure S79). Compound **4** is fluorescent, displaying an emission band between 440 and 550 nm, with a maximum at 484 nm and a tail extending up to 600 nm (Figure 2b). The structured emission is related with strong coupling of vibrational modes with the electronic transition. The emission quantum yield is ϕ = 1% and the fluorescence lifetime is τ_av_ = 3.6 ns. These features agree with those measured for thread **5** and the *hept*-HBC derivative **11** (Figures S71 and S72 and Table S1), demonstrating that the stopper unit is indeed the source of the photoluminescence of rotaxane **4**. Furthermore, the emission spectrum is excitation wavelength independent (Figure S70), highlighting the monomeric nature of the compound in solution and its spectroscopic purity.

[2]Rotaxane **4** also shows nonlinear absorption when excited in the NIR region that roughly follows the linear absorption at half the wavelength (twice the energy). The TPA spectrum was measured by two-photon induced excitation in the 730–900 nm range upon excitation with a high-power density femtosecond laser. An absorption peak is seen within our observation window at ca. 770 nm with a cross-section of σ_2_ = 38 ± 9 GM (Figure 2a). The two-photon induced emission overlaps with that recorded upon conventional one-photon excitation (Figure 2b). A plot of the NIR-excited emission intensity against the irradiation power shows a quadratic dependence (inset in Figure 2b) that confirms the two-photon nature of the process. As expected for the studied system, thread **5** shows a similar behavior, with a TPA of σ_2_ = 40 ± 12 GM (Figure S73) that is equal to that of [2]rotaxane **4**, within experimental error. Likewise, a complete overlap of the one- and two-photon induced emissions is observed (Figure S75). Compound **11** shows similar TPA and TPE spectra, but with a TPA cross-section that is roughly half of the one observed for both rotaxane and the thread (σ_2_ = 17 ± 5 GM, Figure S74). This additive effect was already reported in the literature for systems with multiple unconjugated chromophores^28^ and was expected in our case based on the presence of only one *hept*-HBC unit in the structure of **11** and the lack of conjugation of the *hept*-HBC units in thread **5** and in [2]rotaxane **4** with the rest of the molecule. Moreover, no intermolecular interactions that could affect the optical properties should be present at low concentrations.

These results open the possibility of new designs combining the well-known possibilities of molecular machines with advantages brought about by nonlinear excitation in the NIR region (enhanced light penetration depth in scattering media and high spatial localization) and applications related with nonlinear emission, such as sensing or bioimaging. Moreover, the use of well-defined curved nanographenes with nonhexagonal rings opens the way to exploit more complex structures with enhanced nonlinear responses, including chiral units.

## Conclusions

The inclusion of negatively curved nanographenes as stoppers in a [2]rotaxane is demonstrated for the first time. The saddle-shaped graphene molecule acts as a bulky blocking group and also endorses the final assembly with interesting optical properties. In this sense, high UV–vis absorption and fluorescence emission together with nonlinear optical properties, TPA, and upconverted emission are described. These results represent a proof of concept for a new strategy to introduce nonlinear optical properties in rotaxane architectures. Although further studies on systems based on different interactions or recognition systems are advised to evaluate its scope, a potential advantage of this approach is its versatility as the nonlinear optical properties arise from the stopper unit and should be independent of the recognition motif. Thus, the incorporation of *hept*-HBC stoppers opens a potential way to confer a nonlinear optical response to a wide variety of rotaxane-based molecular devices and machines.

## Data Availability

The data underlying this study are available in the published article and its Supporting Information.

## Supporting Information Available

Synthetic procedures and characterization data, NMR spectra, additional supporting figures, and computational methods (PDF)
