# Supplementary material for: Curved Nanographenes as Stoppers in a [2]Rotaxane with Two-Photon Excited Emission
Source: J Org Chem. 2024 Jun 22;89(13):9344–51. doi: 10.1021/acs.joc.4c00486 (PMC11232015; doi:10.1021/acs.joc.4c00486)
Supplement: Supplementary file 1 — jo4c00486_si_001.pdf [file jo4c00486_si_001.pdf]

Curved Nanographenes as Stoppers in a [2]Rotaxane with Two-Photon Excited Emission

Marcos D. Codesal,[†] Arthur H. G. David,[†] Carla I. M. Santos,[‡] Maria J. Álvaro-Martins,[‡] Ermelinda Maçôas,[‡] Araceli G. Campaña,[†*] and Victor Blanco[†*]

[†] *Departamento de Química Orgánica, Facultad de Ciencias, Universidad de Granada, Avenida Fuente Nueva s/n, 18071, Granada*
[‡] *Centro de Química Estrutural and Institute of Molecular Sciences, Instituto Superior Técnico, Universidade de Lisboa, Av. Rovisco Pais, 1, 1049-001 Lisboa, Portugal*

E-mail: araceligc@ugr.es; victorblancos@ugr.es

- Supporting Information -

Table of contents

1. Experimental procedures **S3**

1.1. General details S3

1.2. Synthesis S4

1.3. Synthetic procedures and characterization details S5

2. NMR spectra **S11**

2.1. Additional NMR stack plots S11

2.2. NMR spectra of new compounds S14

2.3. ^{1}H NMR spectra of known compounds S55

3. MALDI-HRMS spectra **S56**

4. Photophysical properties **S57**

5. Computational methods **S63**

5.1. Geometry optimization S64

5.2. TD-DFT S66

6. References **S68**

1. Experimental procedures

1.1. General details

Unless otherwise stated, all reagents, solvents, HPLC grade solvents and anhydrous solvents were purchased from commercial sources and used without further purification. Anhydrous THF was freshly distilled over Na/benzophenone. Compounds **2**,[S1] **6**,[S2] **7**,[S3] **12**,[S4] and **15**[S5] were prepared according to literature procedures.

Flash column chromatography was carried out using Silica Gel 60 (40-63 μm, VWR) as the stationary phase. Analytical TLC was carried out on aluminium sheets coated with silica gel with fluorescent indicator UV_{254} (Merck Silica Gel 60 F_{254}) and observed under UV light (254 nm) or stained with phosphomolybdic acid (5% ethanol solution) or potassium permanganate (1% w/v in water). Size exclusion chromatography was carried out in a glass column using Biobeads® SX−1 resin beads as stationary phase.

^{1}H and $^{13}C\{^{1}H\}$ NMR spectra were acquired on a Varian Direct Drive (500 MHz or 600 MHz), Bruker Avance III HD NanoBay (400 MHz) or Bruker Avance Neo (400 MHz or 500 MHz) spectrometers at 298 K. Chemical shifts are given in ppm. The signal of the residual protiated solvents (^{1}H: δ = 7.26 for $CDCl_3$, δ = 5.32 for CD_2Cl_2 and δ = 2.50 for DMSO-d_6) or the signal of the TMS (^{1}H: δ = 0.00) were used as reference in the ^{1}H NMR spectra. In the same way, the $^{13}C\{^{1}H\}$ spectra were referenced using the signal of the solvents (^{13}C: δ = 77.16 for $CDCl_3$ and δ = 54.00 for CD_2Cl_2). Coupling constants (*J*) are reported in Hertz (Hz). Standard abbreviations indicating multiplicity were used as follows: m = multiplet, quint = quintet, q = quartet, t = triplet, d = doublet, dd = double doublet, s = singlet, br = broad. The multiplicity of the $^{13}C\{^{1}H\}$ NMR spectra was assigned by means of DEPT experiments. Structural assignments were made with additional information from gCOSY, gHSQC, and gHMBC experiments.

ESI-TOF mass spectra were recorded on a Waters XEVO G2-XS QTof mass spectrometer. MALDI-TOF mass spectra were recorded on a Bruker Ultraflex III mass spectrometer using *trans*-2-[3-(4-*tert*-butylphenyl)-2-methyl-2-propenylidene]malononitrile (DCTB) as matrix, doped with NaI. IR-ATR spectra were recorded on a Perkin Elmer Spectrum Two IR Spectrometer.

1.2. Synthesis

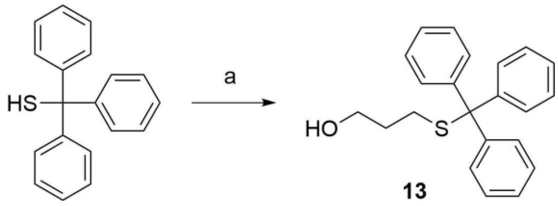

Scheme S1. Synthesis of compound **13**: Reagents and conditions: (a) 3-bromo-1-propanol, LiHMDS, THF, 0 ºC to r.t., 2 h, 74%.

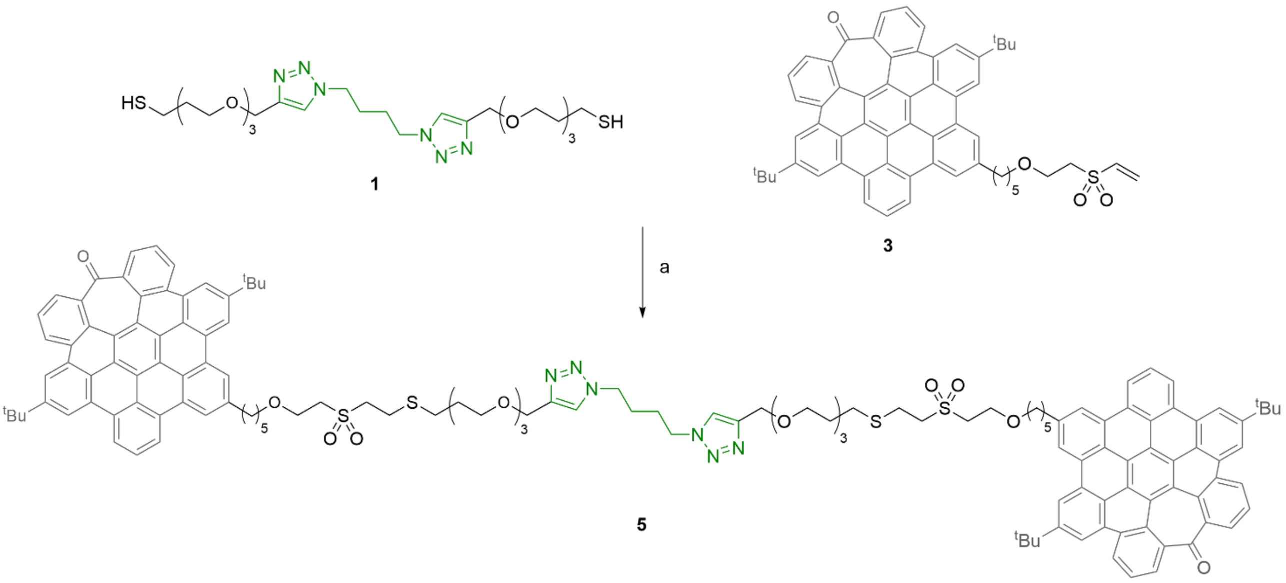

Scheme S2. Synthesis of thread **5**: Reagents and conditions: (a) PPh_3, Et_3N, $CHCl_3$, r.t., 24 h, 50%.

1.3. Synthetic procedures and characterization details

Compound 8

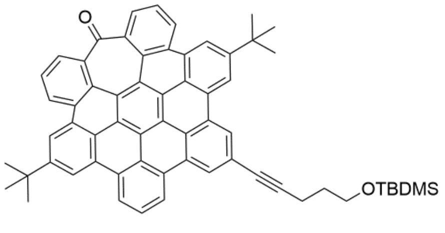

Under Ar, compound **6** (350 mg, 0.444 mmol), $Pd(PPh_3)_2Cl_2$ (16 mg, 0.022 mmol) and CuI (8 mg, 0.044 mmol) were suspended on degassed Et_3N (10 mL). A solution of **7** (264 mg, 1.33 mmol) in degassed anhydrous THF (5 mL) was added to the previous suspension and stirred at room temperature for 20 h. The solvent was removed under reduced pressure. The solid was redissolved in CH_2Cl_2 (100 mL) and washed with $NH_4Cl_{(sat.)}$ (3×100 mL) and H_2O (100 mL). The organic layer was dried over anhydrous Na_2SO_4 and the solvent removed under reduced pressure. The crude was purified by column chromatography (SiO_2, hexane/CH_2Cl_2 1:1) affording **8** as a yellow solid (410 mg, 92%).

^{1}H NMR (500 MHz, $CDCl_3$): δ 8.95 (m, 2H), 8.91 (s, 1H), 8.86 – 8.83 (m, 2H), 8.81 – 8.77 (m, 5H), 7.99 (t, *J* = 7.8 Hz, 1H), 7.89 – 7.83 (m, 4H), 3.91 (t, *J* = 6.0 Hz, 2H), 2.71 (t, *J* = 7.1 Hz, 2H), 1.99 (quint, *J* = 6.6 Hz, 2H), 1.68 (s, 9H), 1.63 (s, 9H), 1.00 (s, 9H), 0.19 (s, 6H). ^{13}C{^{1}H} NMR (126 MHz, $CDCl_3$): δ 202.8 (C), 150.2 (C), 150.1 (C), 142.6 (C), 131.54 (C), 131.47 (C), 130.6 (C), 130.5 (C), 130.1 (C), 129.7 (C), 129.5 (C), 129.2 (C), 128.38 (C), 128.36 (C), 127.7 (CH), 127.5 (C), 127.4 (CH), 126.5 (CH), 125.0 (C), 124.94 (C), 124.91 (CH), 124.8 (CH), 124.7 (C), 124.1 (CH), 123.9 (C), 123.5 (C), 123.3 (C), 123.24 (C), 123.20 (C), 123.0 (C), 122.3 (CH), 121.8 (CH), 121.2 (C), 121.1 (CH), 120.9 (CH), 120.8 (C), 118.5 (CH), 118.3 (CH), 91.4 (C), 81.6 (C), 62.0 (CH_2), 35.8 (C), 35.7 (C), 32.0 (CH_3), 31.9 (CH_3), 29.9 (CH_2), 26.2 (CH_3), 18.6 (C), 16.3 (CH_2). IR (neat): ν 2951, 2927, 2855, 1677, 1608, 1470, 1254, 1101, 832, 758 cm^{-1}. HR-MS (ESI$^+$) m/z: $[M+Na]^+$ Calcd for $C_{62}H_{54}O_2NaSi$: 881.3791; Found 881.3815.

Compound 10

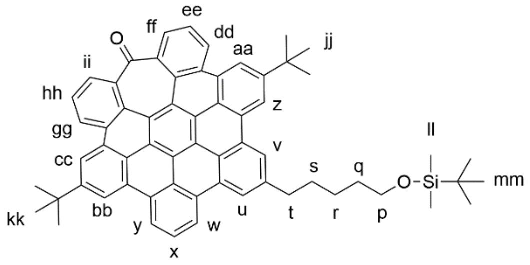

Under Ar, *hept*-HBC derivative **8** (270 mg, 0.314 mmol) was dissolved in a mixture of anhydrous THF/MeOH (10:1, 22 mL). Then, PtO_2 (173 mg, 0.707 mmol) was added, and the resulting suspension was bubbled with Ar. After three vacuum-Ar cycles, H_2 was bubbled into the solution and finally the reaction was stirred under a H_2 atmosphere at room temperature for 24 h. The solvent was removed under reduced pressure and the crude was purified by column chromatography (SiO_2, hexane/CH_2Cl_2 4:6) affording a mixture of **9** and **10** as a yellow solid (213 mg).

This mixture of products (213 mg) was dissolved in anhydrous CH_2Cl_2 (15 mL) and cooled down to 0 ºC. Then, Dess-Martin periodinane (125 mg, 0.295 mmol) was added and the mixture stirred at r.t. for 24 h. The resulting mixture was diluted with CH_2Cl_2 (100 mL) and washed with an aqueous mixed solution of $NaHCO_3$ (0.6 M) and $Na_2S_2O_3$ (0.5 M) (100 mL) and H_2O (100 mL). The organic layer was dried over anhydrous Na_2SO_4

and the solvent removed under reduced pressure. The crude was purified by column chromatography (SiO_2, hexane/CH_2Cl_2 4:6) affording **10** as a yellow solid (199 mg, 73% over two steps).

^{1}H NMR (400 MHz, $CDCl_3$): δ 8.84 (m, 2H, H_{dd}+H_{gg}), 8.76 (s, 1H, H_z/H_{aa}/H_{bb}/H_{cc}), 8.74 (s, 1H, H_z/H_{aa}/H_{bb}/H_{cc}), 8.69 (s, 1H, H_z/H_{aa}/H_{bb}/H_{cc}), 8.67 (s, 1H, H_z/H_{aa}/H_{bb}/H_{cc}), 8.58 (m, 2H, H_w+H_y), 8.46 (s, 1H, H_u/H_v), 8.41 (s, 1H, H_u/H_v), 7.85 (m, 2H, H_{ff}+H_{ii}), 7.77 – 7.71 (m, 3H, H_{ee}+H_{hh}+H_x), 3.67 (t, *J* = 6.5 Hz, 2H, H_p), 2.91 (t, *J* = 7.9 Hz, 2H, H_t), 1.83 (quint, *J* = 7.7 Hz, 2H, H_s), 1.68 – 1.60 (m, 20H, H_q+H_{jj}+H_{kk}), 1.51 (m, 2H, H_r), 0.93 (s, 9H, H_{mm}), 0.09 (s, 6H, H_{ll}). ^{13}C{^{1}H} NMR (101 MHz, $CDCl_3$): δ 203.0 (C), 149.8 (C), 149.7 (C), 142.48 (C), 142.46 (C), 141.8 (C), 131.4 (C), 131.3 (C), 130.3 (C), 130.2 (C), 129.9 (C), 129.8 (C), 129.7 (C), 129.6 (C), 128.31 (C), 128.26 (C), 127.6 (C), 127.5 (C), 127.4 (CH), 127.3 (CH), 127.0 (CH), 126.34 (CH), 126.30 (CH), 124.7 (C), 124.4 (C), 124.3 (C), 123.94 (CH), 123.86 (CH), 123.3 (C), 123.2 (C), 123.1 (C), 122.8 (C), 122.7 (C), 122.0 (CH), 121.6 (CH), 121.5 (CH), 121.4 (CH), 121.1 (C), 120.69 (C), 120.65 (CH), 120.5 (CH), 118.1 (CH), 118.0 (CH), 63.4 (CH_2), 37.0 (CH_2), 35.7 (C), 35.6 (C), 32.9 (CH_2), 32.0 (CH_3), 31.9 (CH_3), 31.8 (CH_2), 26.2 (CH_3), 26.0 (CH_2), 18.5 (C), −5.1 (CH_3). IR (neat): ν 2952, 2926, 2854, 1676, 1610, 1461, 1254, 1093, 830, 758 cm^{-1}. HR-MS (ESI$^+$) m/z: [M+Na]$^+$ Calcd for $C_{62}H_{58}O_2NaSi$: 885.4104; Found 885.4133.

Compound 11

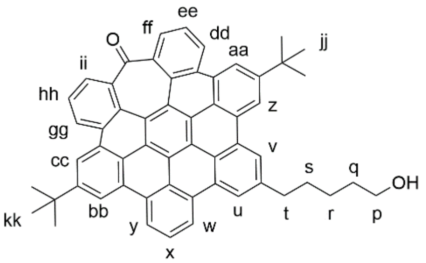

Under Ar, **10** (70 mg, 0.081 mmol) was dissolved in anhydrous THF (10 mL). Then, TBAF (127 mg, 0.487 mmol) was added and the resulting solution was stirred at r.t. for 2 h. The solvent was removed under reduced pressure. The solid was redissolved in EtOAc (50 mL) and washed with $NaHCO_{3(sat.)}$ (3×50 mL). The organic layer was dried over anhydrous Na_2SO_4 and the solvent removed under reduced pressure. The crude was purified by column chromatography (SiO_2, CH_2Cl_2 to CH_2Cl_2/MeOH 98:2) affording **11** as a yellow solid (38 mg, 63%).

^{1}H NMR (500 MHz, $CDCl_3$): δ 8.88 (m, 2H, H_{dd}+H_{gg}), 8.76 (d, *J* = 1.8 Hz, 1H, H_z/H_{aa}/H_{bb}/H_{cc}), 8.73 (m, 2H, H_z/H_{aa}/H_{bb}/H_{cc}+ H_z/H_{aa}/H_{bb}/H_{cc}), 8.70 (d, *J* = 1.8 Hz, 1H, H_z/H_{aa}/H_{bb}/H_{cc}), 8.61 (m, 2H, H_w+H_y), 8.42 (s, 1H, H_u/H_v), 8.39 (s, 1H, H_u/H_v), 7.87 (m, 2H, H_{ff}+H_{ii}), 7.79 (m, 2H, H_{ee}+H_{hh}), 7.75 (t, *J* = 7.8 Hz, 1H, H_x), 3.65 (t, *J* = 6.5 Hz, 2H, H_p), 2.85 (t, *J* = 7.9 Hz, 2H, H_t), 1.79 (quint, *J* = 7.8 Hz, 2H, H_s), 1.65 – 1.61 (m, 20H, H_q+H_{jj}+H_{kk}), 1.48 (m, 2H H_r). ^{13}C{^{1}H} NMR (126 MHz, $CDCl_3$): δ 203.1 (C), 149.9 (C), 149.8 (C), 142.52 (C), 142.50 (C), 141.6 (C), 131.5 (C), 131.4 (C), 130.3 (C), 130.2 (C), 129.9 (C), 129.8 (C), 129.70 (C), 129.67 (C), 128.32 (C), 128.29 (C), 127.61 (C), 127.58 (C), 127.5 (CH), 127.4 (CH), 127.0 (CH), 126.42 (CH), 126.38 (CH), 124.7 (C), 124.5 (C), 124.4 (C), 124.0 (CH), 123.9 (CH), 123.4 (C), 123.21 (C), 123.15 (C), 122.9 (C), 122.7 (C), 122.0 (CH), 121.7 (CH), 121.5 (CH), 121.4 (CH), 121.2 (C), 120.8 (C), 120.7 (CH), 120.6 (CH), 118.11 (CH), 118.06 (CH), 63.0 (CH_2), 36.8 (CH_2), 35.7 (C), 35.6 (C), 32.8 (CH_2), 32.0 (CH_3), 31.9 (CH_3), 31.7 (CH_2), 25.8 (CH_2). IR (neat): ν 2956, 2932, 2864, 1676, 1611, 1335, 1257, 760 cm^{-1}. HR-MS (ESI$^+$) m/z: [M+Na]$^+$ calcd for $C_{56}H_{44}O_2Na$: 771.3239; Found 771.3268.

Compound 3

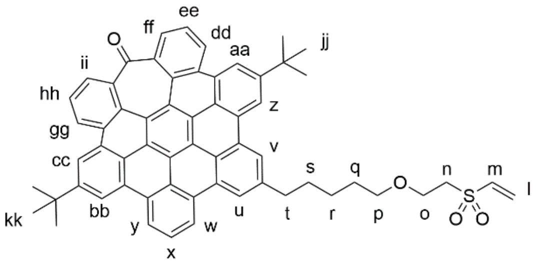

Under Ar, compound **11** (69 mg, 0.092 mmol) was dissolved in anhydrous THF (5 mL). Then, ^{t}BuOK (4 mg, 0.037 mmol) was added and the mixture was stirred at r.t. for 10 min. Divinylsulfone (37 µL, 0.37 mmol) was added to the previous solution and the mixture was stirred at r.t. for additional 45 min. The solvent was removed under reduced pressure. The crude was purified by column chromatography (SiO_2, CH_2Cl_2 to CH_2Cl_2/EtOAc 98:2) to obtain **3** as a yellow solid (42 mg, 53%).

^{1}H NMR (600 MHz, $CDCl_3$): δ 8.97 (m, 2H, H_{dd}+H_{gg}), 8.89 (d, J = 7.8 Hz, 1H, H_y/H_w), 8.83 – 8.79 (m, 5H, H_z+H_{aa}+H_{bb}+H_{cc}+H_y/H_w), 8.69 (s, 1H, H_u/H_v), 8.58 (s, 1H, H_u/H_v), 7.99 (t, J = 7.7 Hz, 1H, H_x), 7.90 – 7.85 (m, 4H, H_{ee}+H_{ff}+H_{hh}+H_{ii}), 6.68 (dd, J = 16.7, 9.9 Hz, 1H, H_m), 6.33 (d, J = 16.6 Hz, 1H, H_l), 5.96 (d, J = 9.9 Hz, 1H, $H_{l'}$), 3.81 (t, J = 5.7 Hz, 2H, H_o), 3.47 (t, J = 6.5 Hz, 2H, H_p), 3.21 (t, J = 5.7 Hz, 2H, H_n), 3.06 (t, J = 7.9 Hz, 2H, H_t), 1.91 (quint, J = 7.7 Hz, 2H, H_s), 1.70 – 1.64 (m, 20H, H_q+H_{jj}+H_{kk}), 1.53 (m, 2H, H_r). ^{13}C{^{1}H} NMR (126 MHz, $CDCl_3$): δ 203.0 (C), 150.09 (C), 150.06 (C), 142.7 (C), 142.6 (C), 141.8 (C), 137.9 (CH), 131.54 (C), 131.50 (C), 130.6 (C), 130.5 (C), 130.2 (C), 130.0 (C), 129.77 (C), 129.75 (C), 128.8 (CH_2), 128.43 (C), 128.39 (C), 127.67 (C), 127.65 (C), 127.62 (CH), 127.56 (CH), 127.3 (CH), 126.54 (CH), 126.51 (CH), 124.9 (C), 124.8 (C), 124.6 (C), 124.1 (CH), 124.0 (CH), 123.5 (C), 123.4 (C), 123.3 (C), 123.00 (C), 122.97 (C), 122.3 (CH), 122.1 (CH), 121.8 (CH), 121.7 (CH), 121.3 (C), 120.90 (CH), 120.87 (C), 120.8 (CH), 118.3 (CH), 118.2 (CH), 71.6 (CH_2), 64.3 (CH_2), 55.2 (CH_2), 37.0 (CH_2), 35.8 (C), 35.7 (C), 32.0 (CH_3), 31.93 (CH_3), 31.88 (CH_2), 29.6 (CH_2), 26.2 (CH_2). IR (neat): ν 2955, 2930, 2864, 1681, 1611, 1318, 1129, 1116, 761 cm^{-1}. HR-MS (ESI$^+$) m/z: $[M+Na]^+$ Calcd for $C_{60}H_{50}O_4NaS$: 889.3328; Found 889.3353.

Compound 13

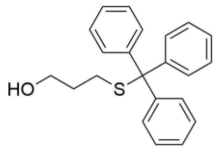

Under Ar, triphenylmethanethiol (2.00 g, 7.24 mmol) was dissolved in anhydrous THF (25 mL) and stirred at 0 ºC for 10 min. LiHMDS (7.24 mL, 1 M in THF, 7.24 mmol) was added and stirred at 0 ºC until a precipitate was formed. Then, 3-bromo-1-propanol (595 µL, 6.58 mmol) was added and the mixture was stirred at r.t. for 2 h. The solvent was removed under reduced pressure. The solid was redissolved in EtOAc (200 mL) and washed with H_2O (3×200 mL). The organic layer was dried over anhydrous Na_2SO_4 and the solvent removed under reduced pressure. The crude was purified by column chromatography (SiO_2, CH_2Cl_2) affording **13** as a white solid (1.63 g, 74%).

^{1}H NMR (400 MHz, $CDCl_3$): δ 7.46 (d, J = 7.8 Hz, 6H), 7.31 (t, J = 7.5 Hz, 6H), 7.23 (t, J = 7.0 Hz, 3H), 3.55 (t, J = 6.2 Hz, 2H), 2.30 (t, J = 7.2 Hz, 2H), 1.64 (quint, J = 6.7 Hz, 2H). Spectroscopic data agree with the values previously reported.[S6]

Compound 14

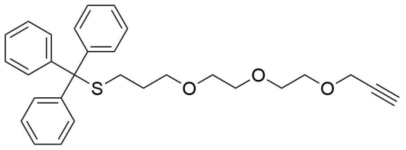

Safety note: *NaH releases flammable gases (H_2) in contact with water. Anhydrous solvent should be use and precautions upon quenching of the reaction should be taken (slow addition of water while cooling in a water ice-bath), specially if the reaction is scaled up or a larger excess is used. Moreover, it has been reported that NaH is incompatible with DMSO, DMF or N,N-dimethylacetamide (Org. Process Res. Dev.* ***2019****, 23, 2210) as explosive mixtures are formed. Therefore, those combinations must be avoided.*

Under an Ar atmosphere, **13** (510 mg, 1.53 mmol) was dissolved in anhydrous THF (15 mL). NaH (110 mg, 60% in mineral oil, 2.75 mmol) was added and stirred at 0 ºC for 10 min. A solution of **12** (682 mg, 2.29 mmol) in anhydrous THF (15 mL) and a catalytic amount of $^{n}Bu_4NI$ were added and refluxed for 24 h. The solvent was removed under reduced pressure. The solid was redissolved in EtOAc (150 mL) and washed with H_2O (3×100 mL). The organic layer was dried over anhydrous Na_2SO_4 and the solvent removed under reduced pressure. The crude was purified by column chromatography (SiO_2, hexane/EtOAc 8:2) giving **14** as a white solid (374 mg, 55%).

^{1}H NMR (400 MHz, CD_2Cl_2): δ 7.42 (m, 6H), 7.29 (m, 6H), 7.21 (m, 3H), 4.20 (d, *J* = 2.3 Hz, 2H), 3.67 (m, 4H), 3.60 (m, 2H), 3.52 (m, 2H), 3.41 (t, *J* = 6.4 Hz, 2H), 2.43 (t, *J* = 2.7 Hz, 1H), 2.25 (t, *J* = 7.3 Hz, 2H), 1.66 (quint, *J* = 6.9 Hz, 2H). ^{13}C{^{1}H} NMR (101 MHz, CD_2Cl_2): δ 145.6 (C), 130.1 (CH), 128.4 (CH), 127.1 (CH), 80.4 (C), 74.7 (CH), 71.0 (CH_2), 70.9 (CH_2), 70.7 (CH_2), 70.3 (CH_2), 69.8 (CH_2), 67.0 (C), 58.8 (CH_2), 29.3 (CH_2), 29.2 (CH_2). IR (neat): ν 3296, 2864, 1594, 1488, 1443, 1099, 737, 698 cm^{-1}. HR-MS (ESI^+) m/z: $[M+Na]^+$ Calcd for $C_{29}H_{32}O_3NaS$: 483.1970; Found 483.1979.

Compound 16

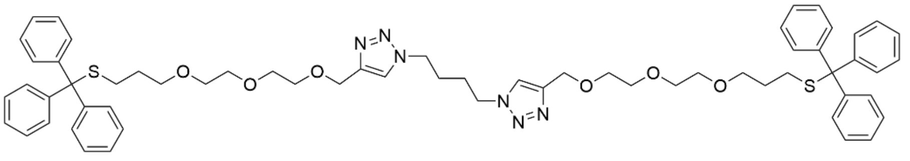

Safety note: *Low-molecular weight azides have been reported to be heat and shock sensitive and* ***explosive****. Therefore, safety precautions (e.g. protecting screen) should be taken with diazide* ***15*** *during the synthesis, workup and concentration steps. We did not have any problems working on this small scale, but scaling up the reaction should be avoided for safety reasons.*

Under Ar, diazide **15** (56 mg, 0.40 mmol) was dissolved in anhydrous CH_2Cl_2 (2 mL). Then, a solution of **14** (717 mg, 1.60 mmol) in anhydrous CH_2Cl_2 (2 mL), $Cu(CH_3CN)_4PF_6$ (30 mg, 0.080 mmol) and TBTA (42 mg, 0.08 mmol) were added and the mixture was stirred at r.t. for 24 h. The solvent was removed under reduced pressure and the crude was purified by column chromatography (SiO_2, CH_2Cl_2/MeOH 96:4) affording **16** as a white solid (391 mg, 94%).

^{1}H NMR (400 MHz, $CDCl_3$): δ 7.57 (br, 2H), 7.42 (m, 12H), 7.29 (m, 12H), 7.22 (m, 6H), 4.69 (br, 4H), 4.35 (br, 4H), 3.71 – 3.64 (m, 8H), 3.60 (m, 4H), 3.52 (m, 4H), 3.41 (t, *J* = 6.4 Hz, 4H), 2.25 (t, *J* = 7.2 Hz, 4H), 1.93 (s, 4H), 1.66 (quint, *J* = 6.8 Hz, 4H). ^{13}C{^{1}H} NMR (126 MHz, $CDCl_3$): δ 145.0 (C), 129.7 (CH), 127.9 (CH), 126.7 (CH), 70.64 (CH_2), 70.63 (CH_2), 70.2 (CH_2), 70.0 (CH_2), 69.9 (CH_2), 66.6 (C), 64.8 (CH_2), 49.4 (CH_2), 28.8 (CH_2), 27.2 (CH_2). IR (neat): ν 2864, 1488, 1443, 1093, 1047, 843, 742, 699 cm^{-1}. HR-MS (ESI^+) m/z: $[M+Na]^+$ Calcd for $C_{62}H_{72}N_6O_6NaS_2$: 1083.4852; Found 1083.4878.

Compound 1

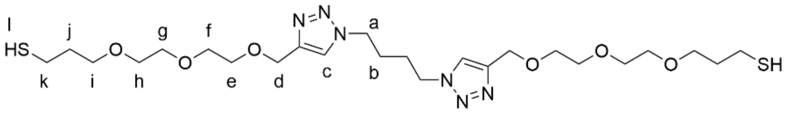

Under Ar, **16** (68 mg, 0.066 mmol) was dissolved in anhydrous CH_2Cl_2 (4 mL). Then, CF_3CO_2H (1 mL) and Et_3SiH (0.5 mL) were added, and the mixture stirred at r.t. for 5 h. The solvent was removed under reduced pressure and the crude was redissolved in toluene (5 mL) and evaporated again under vacuum (this process was repeated 3 times). The resulting crude was purified by column chromatography (SiO_2, CH_2Cl_2/MeOH 96:4) to obtain compound **1** as a white solid (37 mg, 98%).

^{1}H NMR (400 MHz, $CDCl_3$): δ 7.54 (s, 2H, H_c), 4.67 (s, 4H, H_d), 4.36 (br, 4H, H_a), 3.70 – 3.54 (m, 20H, H_e-H_i), 2.59 (m, 4H, H_k), 1.93 (br, 4H, H_b), 1.86 (m, 4H, H_j), 1.37 (t, J = 8.0 Hz, 2H, H_l). ^{13}C{^{1}H} NMR (101 MHz, $CDCl_3$): δ 145.6 (C), 122.6 (CH), 70.69 (CH_2), 70.67 (CH_2), 70.3 (CH_2), 70.0 (CH_2), 69.2 (CH_2), 64.8 (CH_2), 49.4 (CH_2), 33.8 (CH_2), 27.2 (CH_2), 21.5 (CH_2). IR (neat): ν 3462, 2920, 2867, 1461, 1441, 1089, 1049, 732 cm^{-1}. HR-MS (ESI$^+$) m/z: $[M+Na]^+$ Calcd for $C_{24}H_{44}N_6O_6NaS_2$: 599.2661; Found 599.2675.

Thread 5

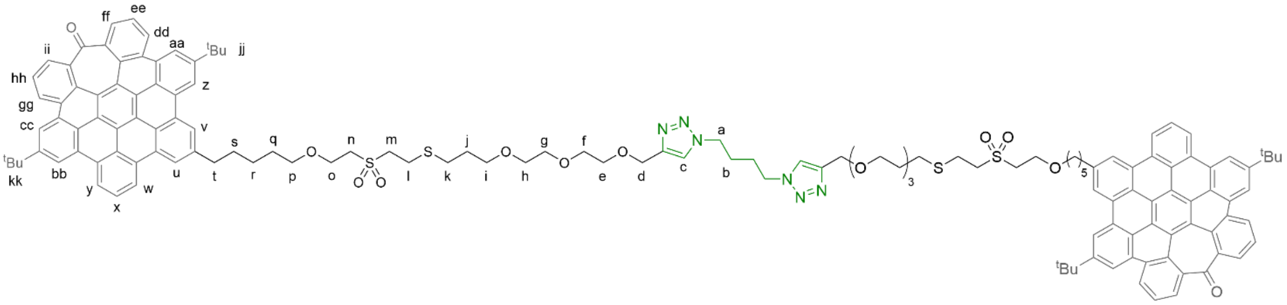

Under an Ar atmosphere, axle **1** (15 mg, 0.026 mmol), *hept*-HBC **3** (90 mg, 0.10 mmol) and PPh_3 (68 mg, 0.26 mmol) were dissolved in degassed $CHCl_3$ (1.5 mL). Then, 4 drops of degassed Et_3N were added and the mixture was stirred at r.t. for 24 h. The solvent was removed under reduced pressure and the crude was purified by column chromatography (SiO_2, CH_2Cl_2/EtOAc 98:2 to 90:10 to CH_2Cl_2/MeOH 95:5) and subsequently by size exclusion chromatography (Bio-Beads® SX−1, CH_2Cl_2) affording **5** as a yellow solid (30 mg, 50%).

^{1}H NMR (400 MHz, $CDCl_3$): δ ^{1}H NMR (400 MHz, $CDCl_3$) δ: 8.96 – 8.76 (m, 16H, H_w+H_y+H_z+H_{aa}+H_{bb}+H_{cc}+H_{dd}+H_{gg}), 8.68 (br, 2H, H_u/H_v) 8.57 (s, 2H, H_u/H_v), 7.97 (m, 2H, H_x), 7.90 – 7.82 (m, 8H, H_{ee}+H_{ff}+H_{hh}+H_{ii}), 7.31 (s, 2H, H_c), 4.52 (s, 4H, H_d), 4.13 (br, 4H, H_a), 3.81 (t, J = 5.3 Hz, 4H, H_o), 3.55 – 3.39 (m, 24H, H_e+H_f+H_g+H_h+H_i+H_p), 3.33 (m, 4H, H_m), 3.19 (t, J = 5.3 Hz, 4H, H_n), 3.05 (br, 4H, H_t), 2.88 (m, 4H, H_l), 2.56 (t, J = 7.2 Hz, 4H, H_k), 1.90 (m, 4H, H_s), 1.77 (m, 4H, H_j), 1.72 – 1.60 (m, 44H, H_b+H_q+H_{jj}+H_{kk}), 1.53 (m, 4H, H_r). ^{13}C{^{1}H} NMR (126 MHz, $CDCl_3$): δ 202.9 (C), 150.13 (C), 150.10 (C), 145.3 (C), 142.61 (C), 142.58 (C), 141.8 (C), 131.51 (C), 131.46 (C), 130.6 (C), 130.5 (C), 130.2 (C), 130.0 (C), 129.8 (2×C), 128.40 (C), 128.36 (C), 127.64 (CH), 127.60 (C), 127.58 (CH), 127.4 (CH), 126.53 (CH), 126.51 (CH), 124.8 (C), 124.7 (C), 124.5 (C), 124.04 (CH), 124.00 (CH), 123.4 (C), 123.31 (C), 123.26 (C), 123.0 (C), 122.9 (C), 122.6 (CH), 122.4 (CH), 122.1 (CH), 121.8 (CH), 121.7 (CH), 121.3 (C), 120.90 (CH), 120.87 (CH), 120.8 (C), 118.3 (CH), 118.2 (CH), 71.7 (CH_2), 70.6 (2×CH_2), 70.2 (CH_2), 69.8 (CH_2), 69.4 (CH_2), 64.7 (CH_2), 64.5 (CH_2), 55.2 (CH_2), 54.0 (CH_2), 49.2 (CH_2), 37.0 (CH_2), 35.8 (C), 35.7 (C), 32.0 (CH_3), 31.9 (CH_3+CH_2^*), 29.6 (CH_2), 29.5 (CH_2), 28.9 (CH_2), 27.1 (CH_2), 26.3 (CH_2), 24.1 (CH_2). IR (neat): ν 2952, 2924, 2855, 1676, 1319, 1258, 1111, 761, 734 cm^{-1}. HR-MS (MALDI$^+$) m/z: $[M+Na]^+$ Calcd for $C_{144}H_{144}N_6NaO_{14}S_4$: 2331.9516; Found 2331.9478.

* Signal located from the HSQC experiment.

Rotaxane 4

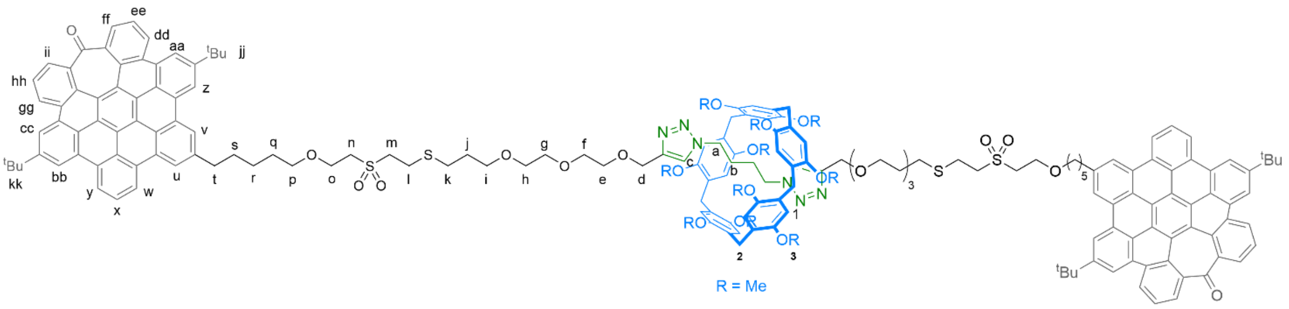

Under Ar, axle **1** (6 mg, 0.010 mmol) and per-*O*-methyl-pillar[5]arene (**2**) (37 mg, 0.049 mmol) were dissolved in degassed $CHCl_3$ (1.5 mL) and stirred at r.t. for 5 h. Then, this solution was transferred under Ar to a separate flask containing **3** (26 mg, 0.030 mmol) and PPh_3 (26 mg, 0.099 mmol), previously purged with Ar. Finally, 4 drops of degassed Et_3N were added to the mixture, which was further stirred at r.t. under an Ar atmosphere for 24 h. The solvent was removed under reduced pressure and the crude was purified by column chromatography (SiO_2, CH_2Cl_2/EtOAc 98:2 to 90:10 to CH_2Cl_2/MeOH 95:5) and then by size exclusion chromatography (Bio-Beads® SX−1, CH_2Cl_2) affording rotaxane **4** as a yellow solid (12 mg, 40%).

^{1}H NMR (400 MHz, $CDCl_3$): δ 9.02 (m, 6H, $H_w/H_y+H_{gg}+H_{dd}$), 8.89 – 8.83 (m, 12H, $H_u/H_v+H_w/H_y+H_z+H_{aa}+H_{bb}+H_{cc}$), 8.66 (s, 2H, H_u/H_v), 8.09 (t, *J* = 7.8 Hz, 2H, H_x), 7.90 (m, 8H, $H_{ee}+H_{ff}+H_{hh}+H_{ii}$), 7.19 (s, 2H, H_c), 6.81 (s, 10H, H_1), 4.63 (s, 4H, H_d), 3.83 (t, *J* = 5.3 Hz, 4H, H_o), 3.69 (s, 10H, H_2), 3.63 – 3.44 (m, 54H, $H_3+H_e+H_f+H_g+H_h+H_i+H_p$), 3.33 (m, 4H, H_m), 3.20 – 3.14 (m, 8H, H_n+H_t), 2.89 (m, 4H, H_l), 2.58 (t, *J* = 7.2 Hz, 4H, H_k), 1.97 (m, 8H, H_a+H_s), 1.81 (quint, *J* = 6.6 Hz, 4H, H_j), 1.67 (m, 44H, $H_q+H_r+H_{jj}+H_{kk}$), −1.24 (br, 4H, H_b). ^{13}C{^{1}H} NMR (126 MHz, $CDCl_3$): δ 202.9 (C), 150.4 (C), 150.2 (C), 150.1 (C), 144.1 (C), 142.69 (C), 142.66 (C), 141.9 (C), 131.6 (C), 131.5 (C), 130.70 (C), 130.65 (C), 130.3 (C), 130.1 (C), 129.83 (C), 129.79 (C), 128.7 (C), 128.5 (C), 128.4 (C), 127.70 (C), 127.67 (CH), 127.6 (CH), 127.4 (CH), 126.59 (CH), 126.55 (CH), 124.93 (C), 124.87 (C), 124.6 (C), 124.1 (CH), 124.0 (CH), 123.5 (C), 123.42 (C), 123.35 (C), 123.1 (C), 123.0 (C), 122.5 (CH), 122.2 (CH), 121.9 (2×CH), 121.8 (CH), 121.3 (C), 121.0 (CH), 120.9 (CH), 118.30 (CH), 118.25 (CH), 113.7 (CH), 71.7 (CH_2), 70.71 (CH_2), 70.70 (CH_2), 70.4 (CH_2), 70.0 (CH_2), 69.5 (CH_2), 64.8 (CH_2), 64.5 (CH_2), 55.8 (CH_3), 55.2 (CH_2), 54.1 (CH_2), 48.3 (CH_2), 37.1 (CH_2), 35.8 (C), 35.7 (C), 32.1 (CH_2), 32.0 (CH_3), 31.9 (CH_3), 29.6 (CH_2), 29.5 (CH_2), 29.1 (CH_2), 29.0 (CH_2), 26.3 (CH_2), 24.2 (CH_2), 24.1 (CH_2). IR (neat): ν 2957, 2926, 2855, 1611, 1318, 1258, 1213, 1045, 733 cm^{-1}. HR-MS (MALDI$^+$) m/z: $[M+Na]^+$ Calcd for $C_{189}H_{194}N_6NaO_{24}S_4$: 3082.2920; Found 3082.2948.

2. NMR spectra

2.1. Additional NMR stack plots

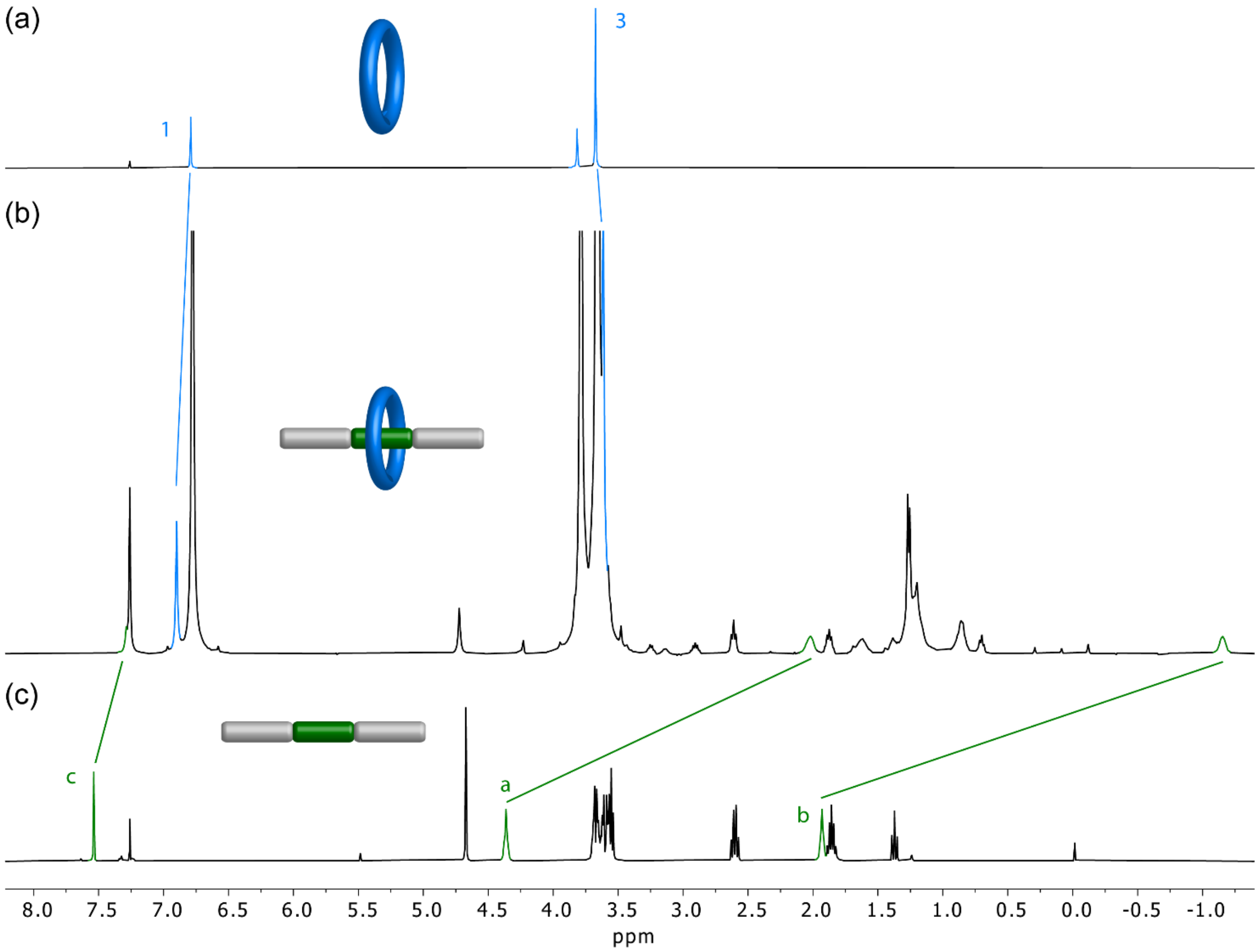

Figure S1. ^{1}H NMR (400 MHz, $CDCl_3$) spectra of: (a) Pillar[5]arene **2**; (b) pseudorotaxane assembled from thread precursor **1** and an excess of macrocycle **2**; (c) compound **1**. The assignment and color coding corresponds to that shown in Scheme 1 in the main text. In spectrum (b) only the signals corresponding to the complexed macrocycle are shown in blue.

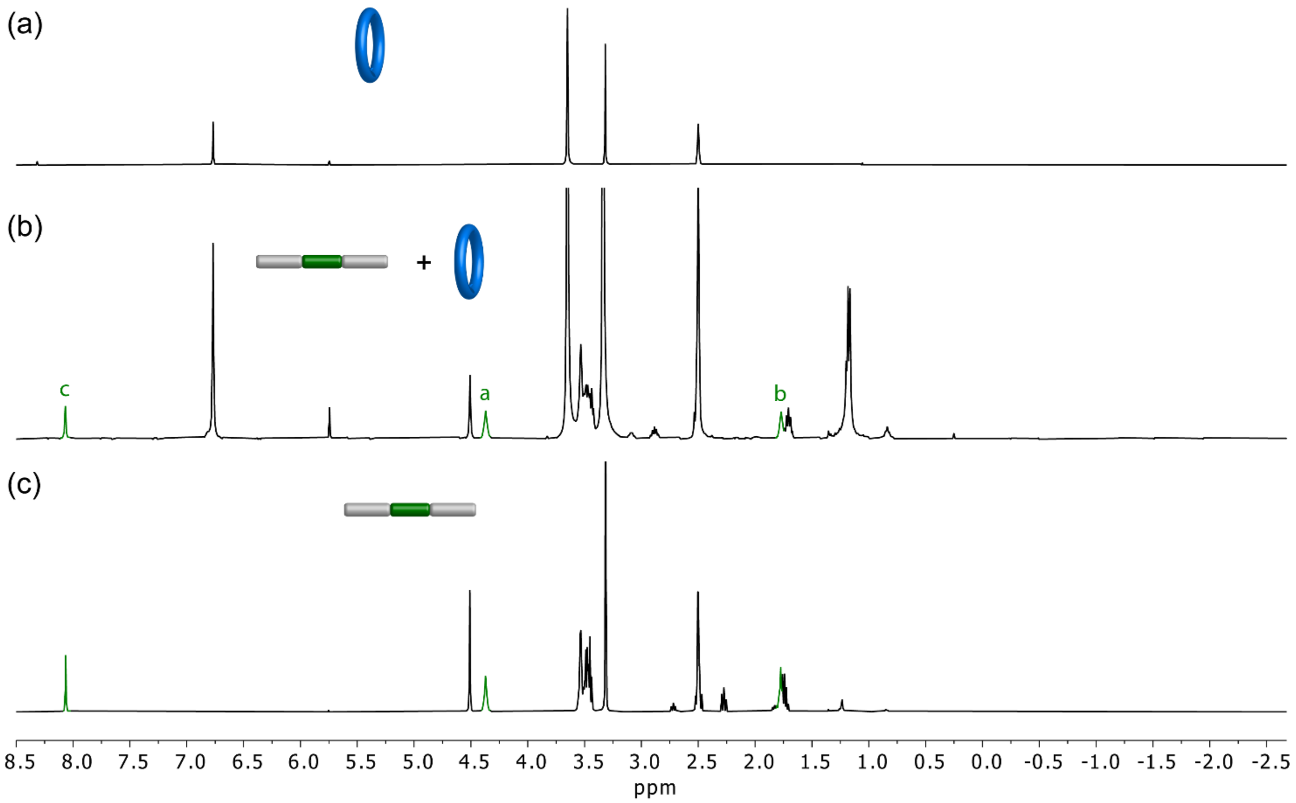

Figure S2. ^{1}H NMR (400 MHz, DMSO-d_6) spectra of: (a) Pillar[5]arene **2**; (b) mixture of axle **1** and an excess of macrocycle **2**; (c) compound **1**. Spectrum (b) shows that the chemical shift of the signals of both **1** and the macrocycle remain unaltered when both compounds are mixed in DMSO-d_6. This lack of shifting of the signals demonstrates that there are no interactions between the recognition motif and the macrocycle in that solvent and, therefore, no pseudorotaxane is formed.

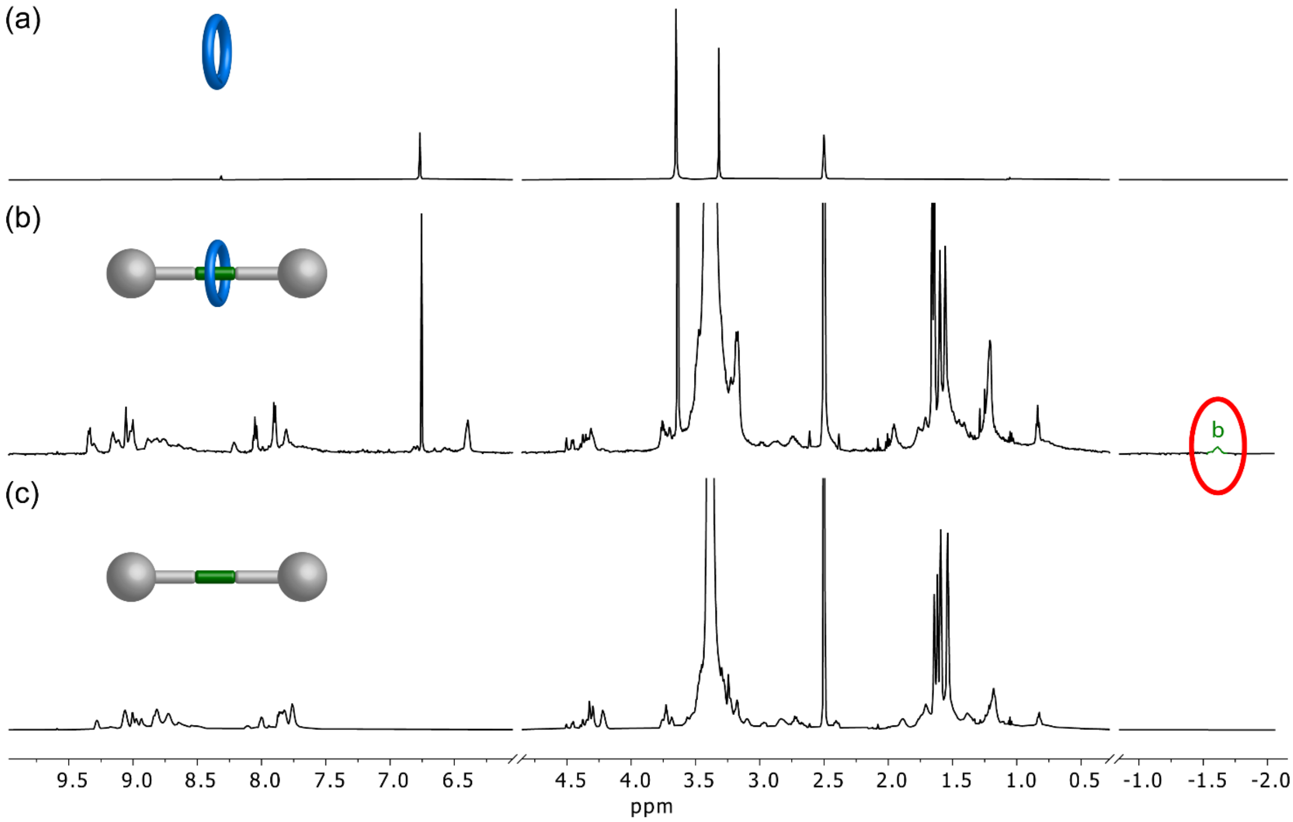

Figure S3. ^{1}H NMR (DMSO-d_6) spectra of: (a) Pillar[5]arene **2** (400 MHz); (b) rotaxane **4** (600 MHz); (c) thread **5** (600 MHz). A signal at $\delta = -1.61$ ppm can be seen in spectrum (b), showing that there are strong interactions between the macrocycle and the 4-di(1,2,3-triazol-1-yl)-butane recognition motif on the thread in this case, supporting the interlocked nature of the compound **4**.

2.2. NMR spectra of new compounds

Compound 8

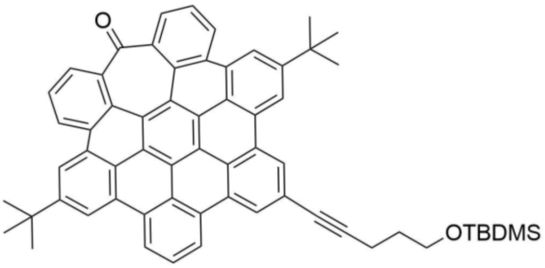

8

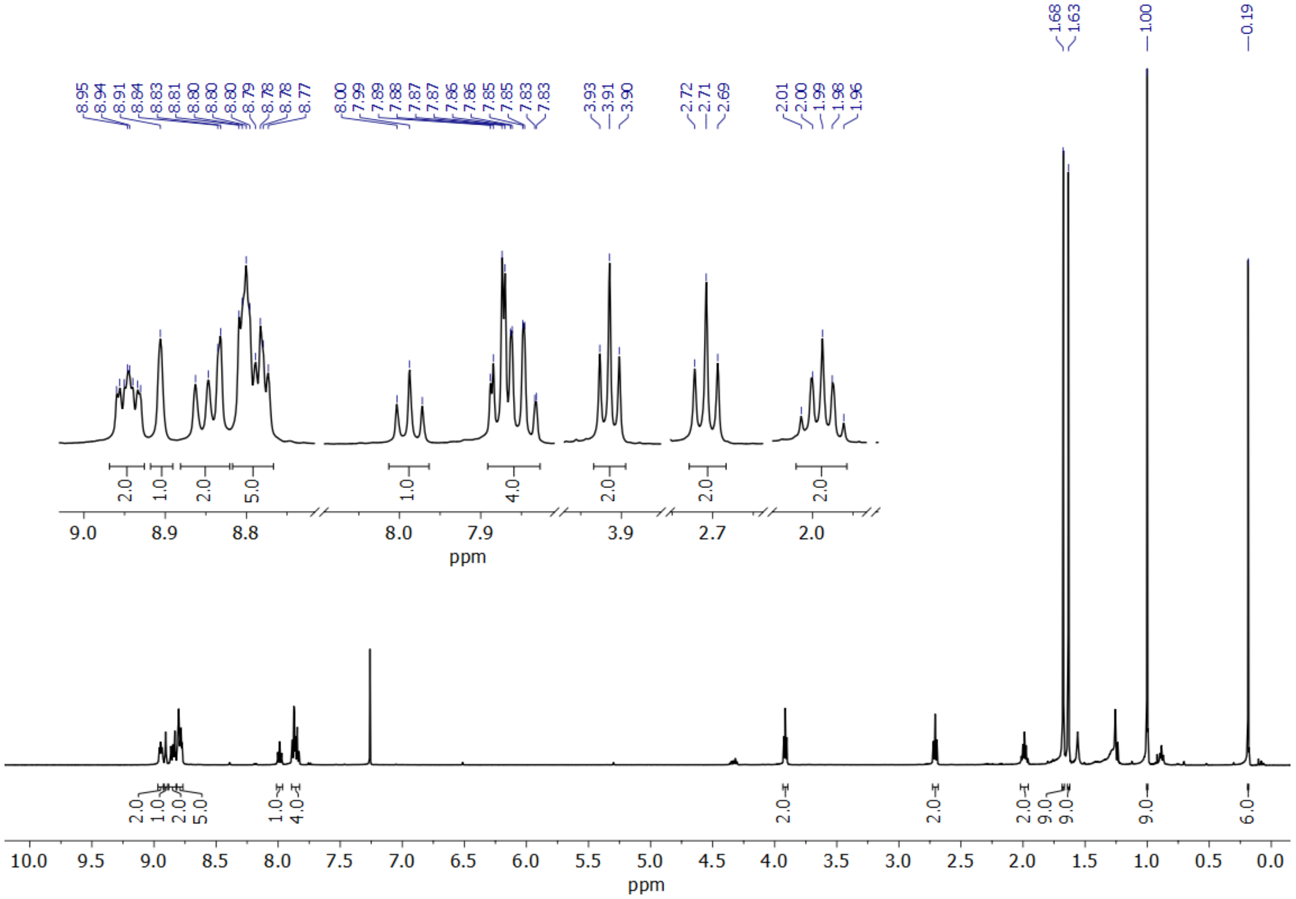

Figure S3. ^{1}H NMR (500 MHz, $CDCl_3$) spectrum of compound **8**.

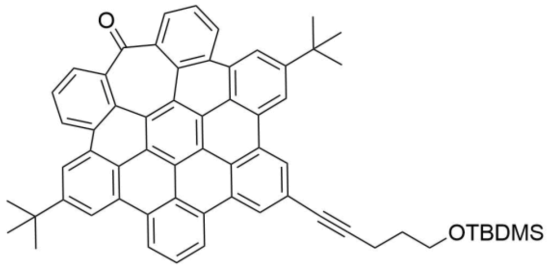

8

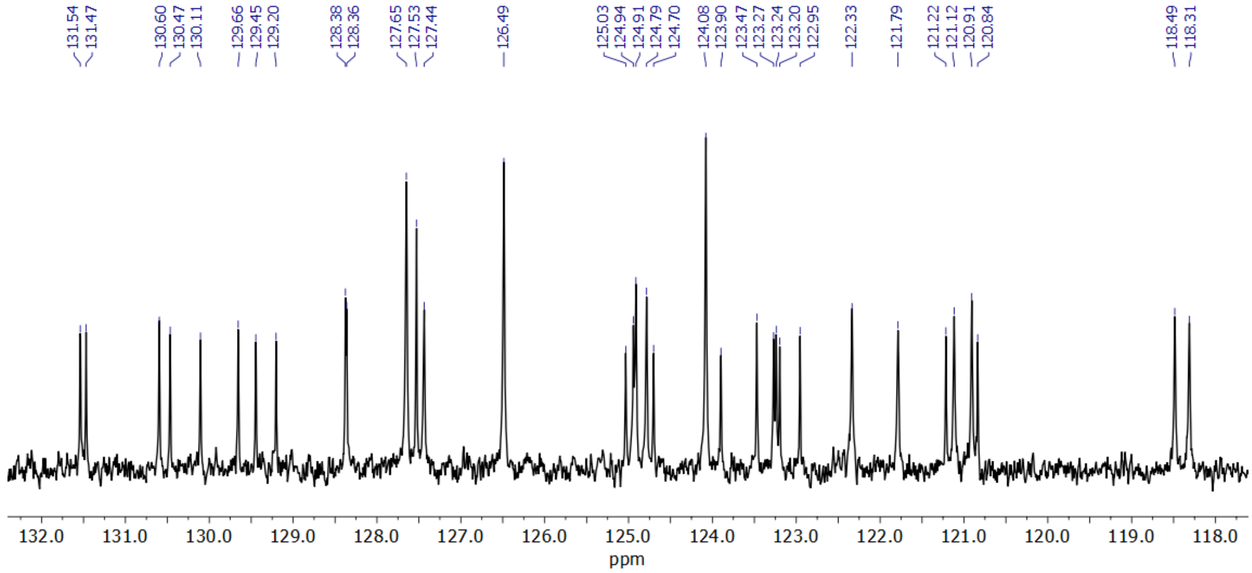

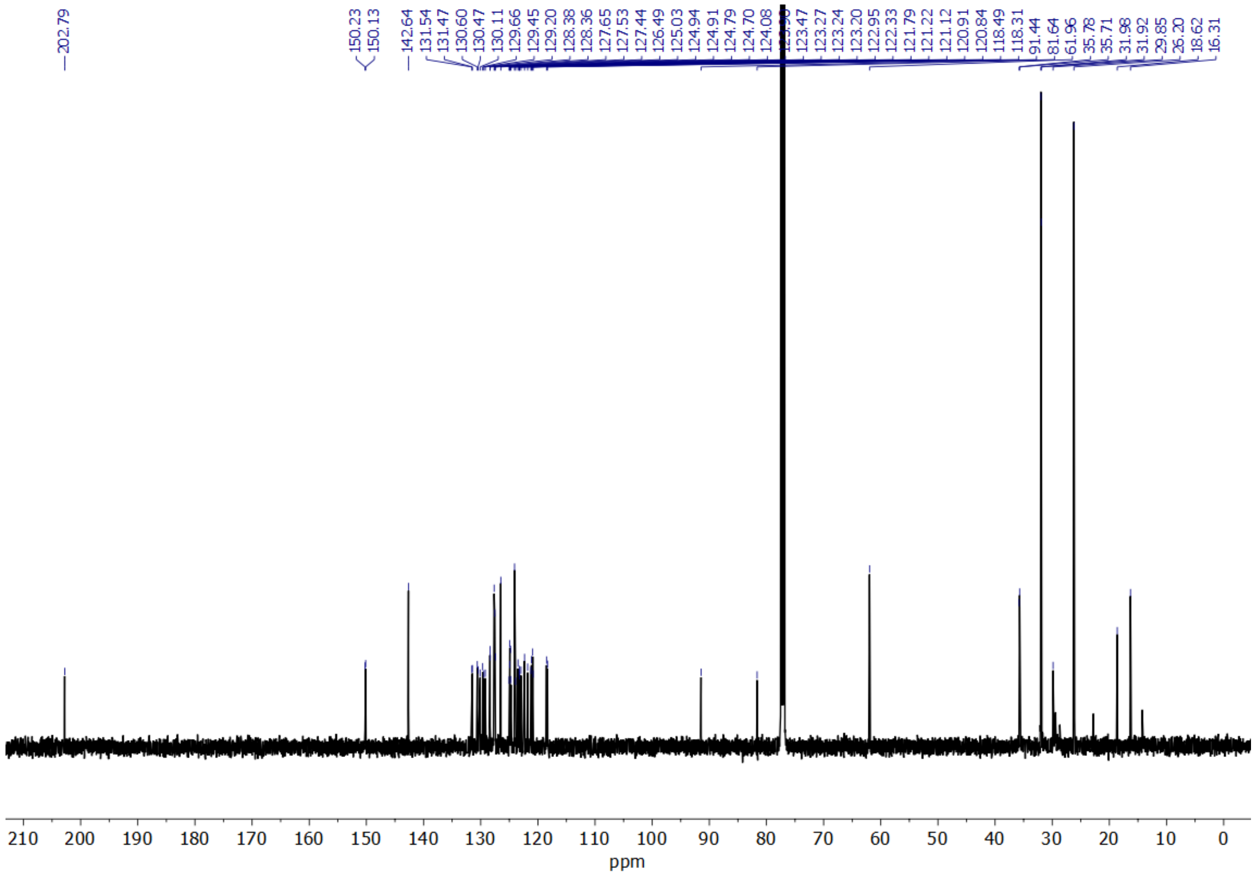

Figure S4. $^{13}C\{^{1}H\}$ NMR (126 MHz, $CDCl_3$) spectrum of compound **8**.

Compound 10

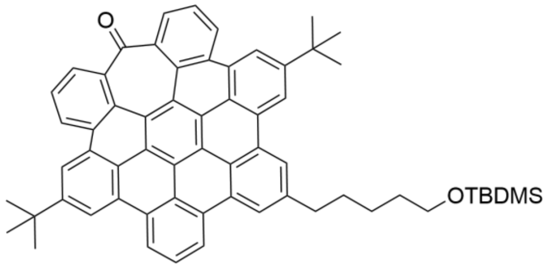

10

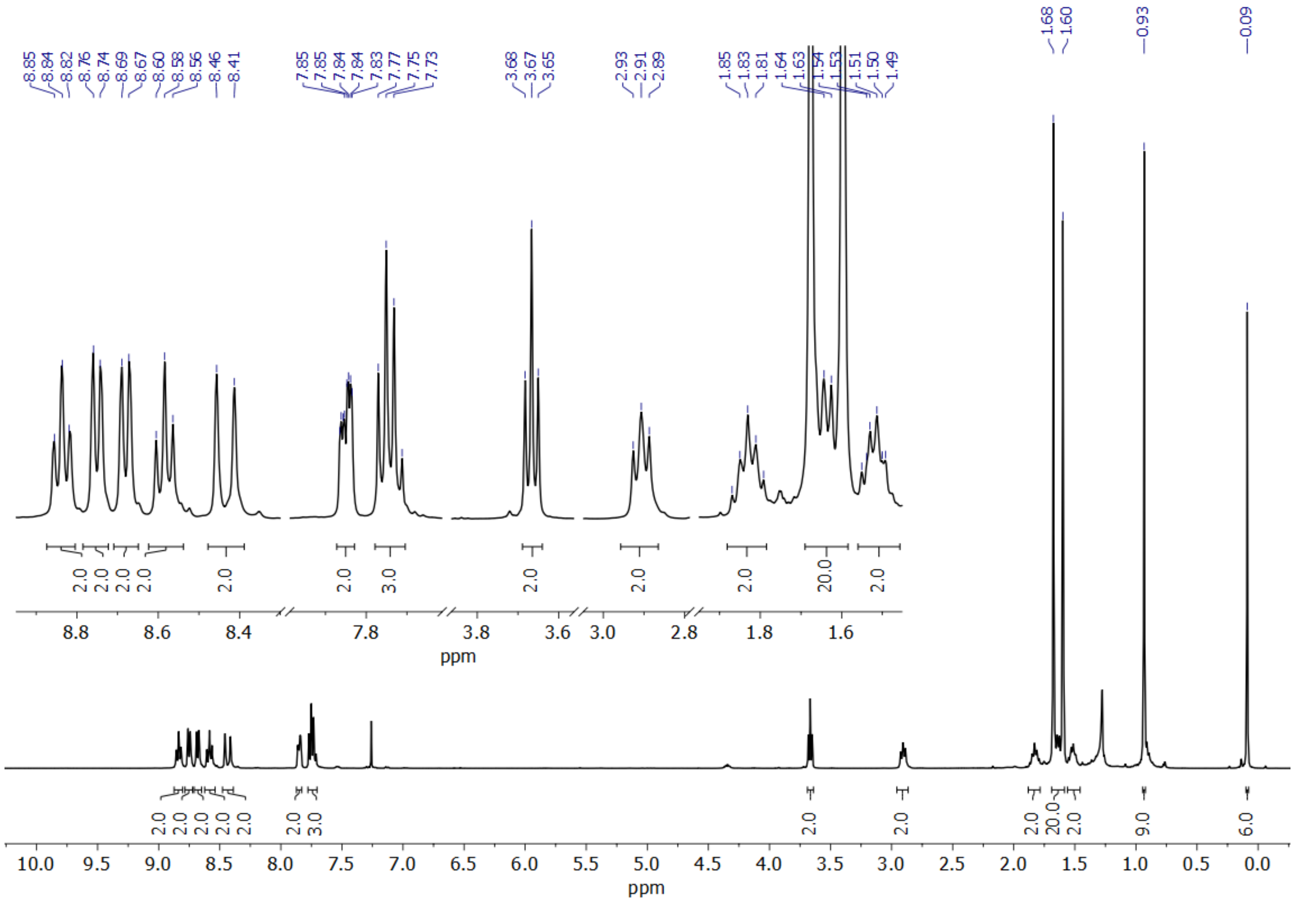

Figure S5. ^{1}H NMR (400 MHz, $CDCl_3$) spectrum of compound **10**.

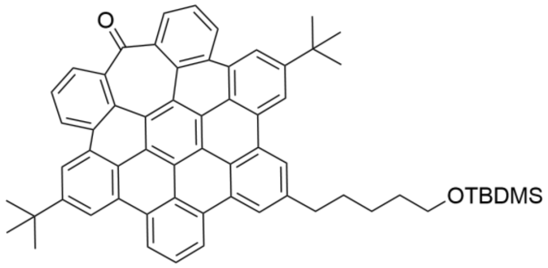

10

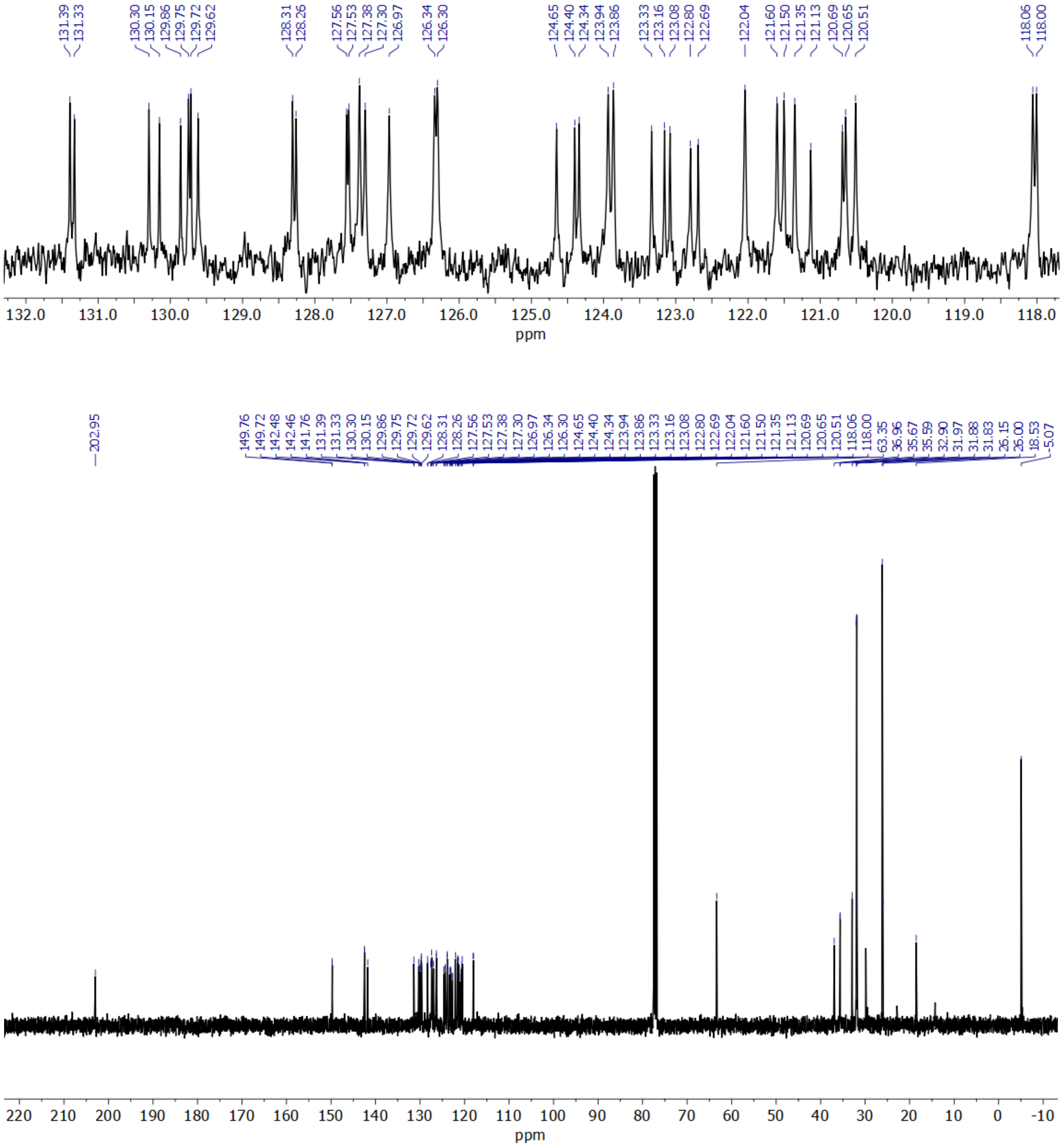

Figure S6. $^{13}C\{^{1}H\}$ NMR (101 MHz, $CDCl_3$) spectrum of compound **10**.

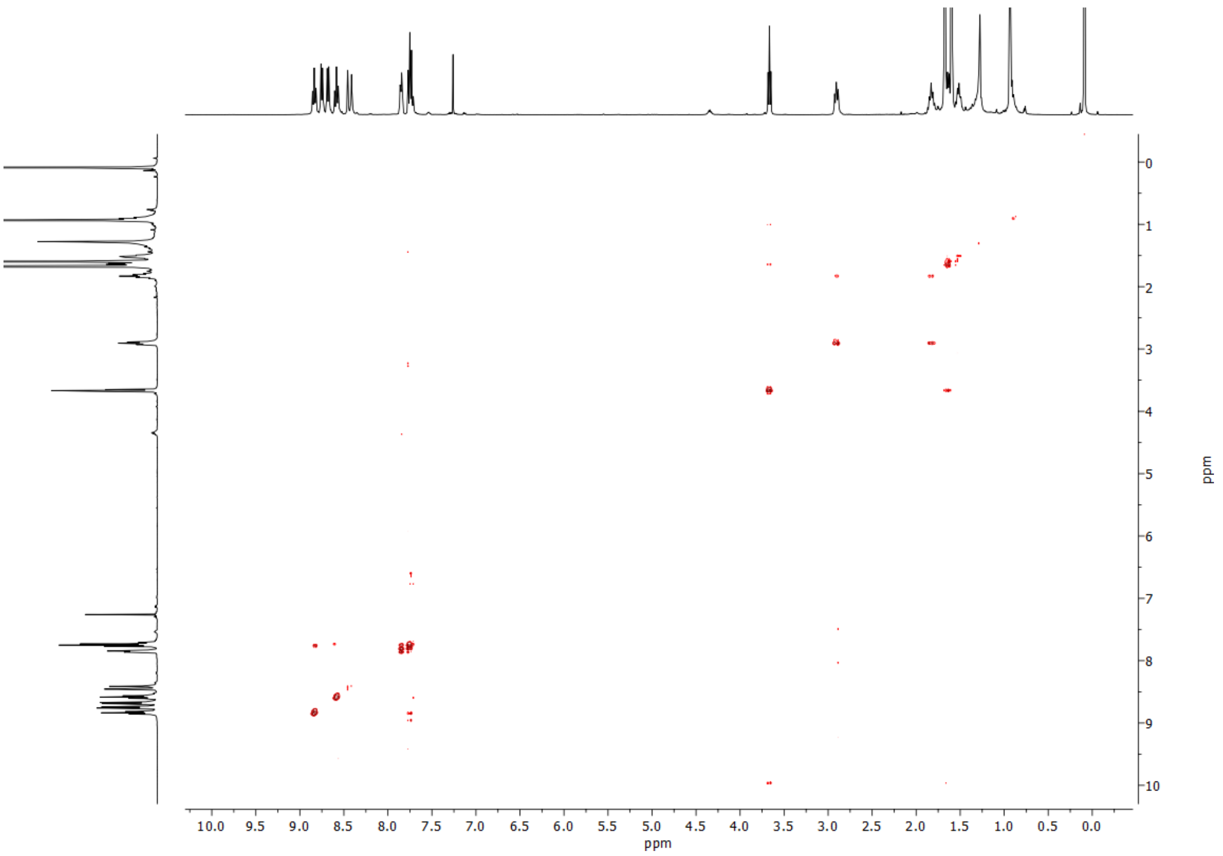

Figure S7. COSY NMR (400 MHz, $CDCl_3$) spectrum of compound **10**.

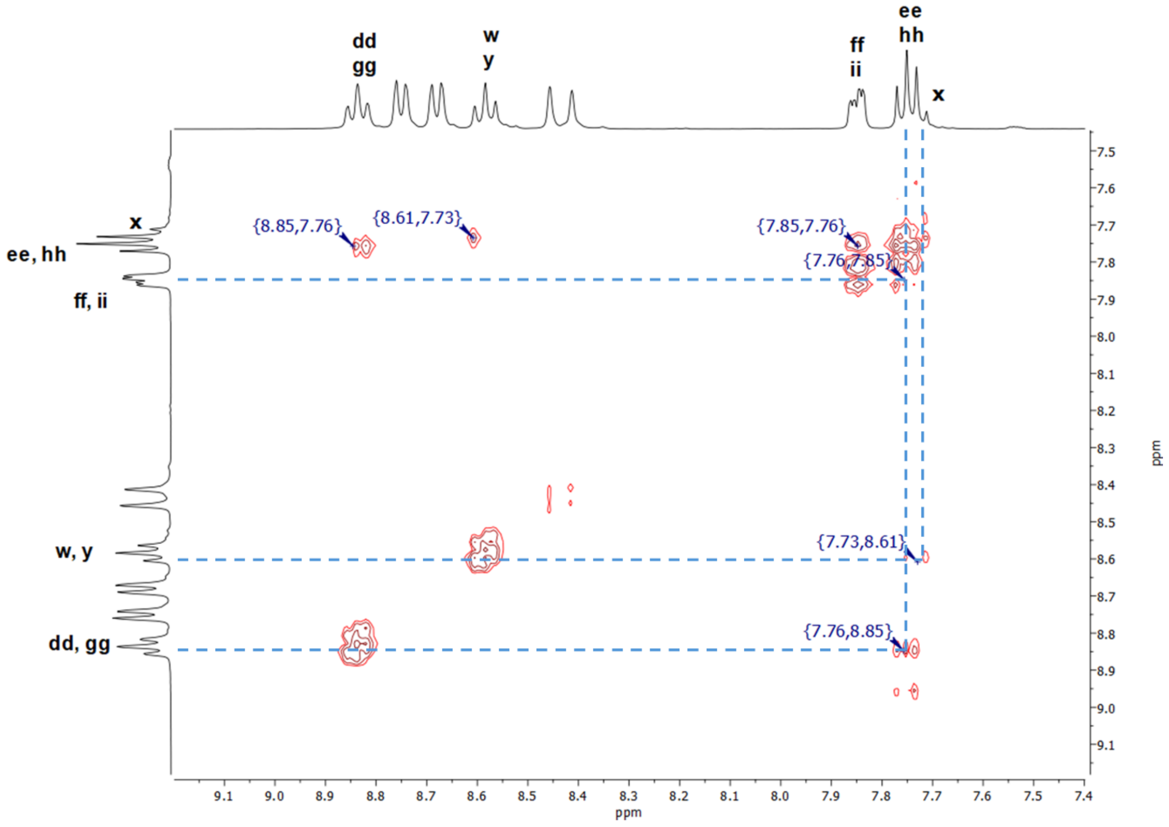

Figure S8. Partial COSY NMR (400 MHz, $CDCl_3$) spectrum of compound **10**.

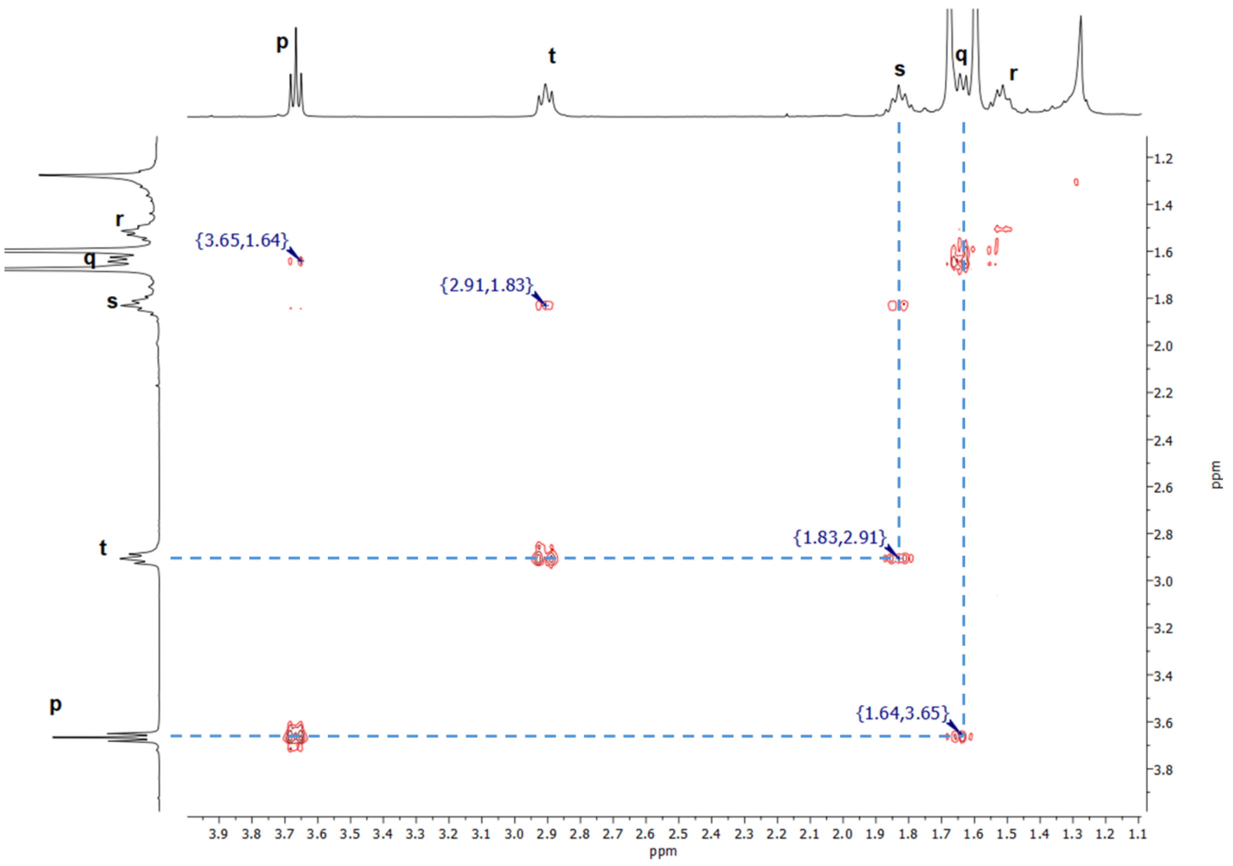

Figure S9. Partial COSY NMR (400 MHz, $CDCl_3$) spectrum of compound **10**.

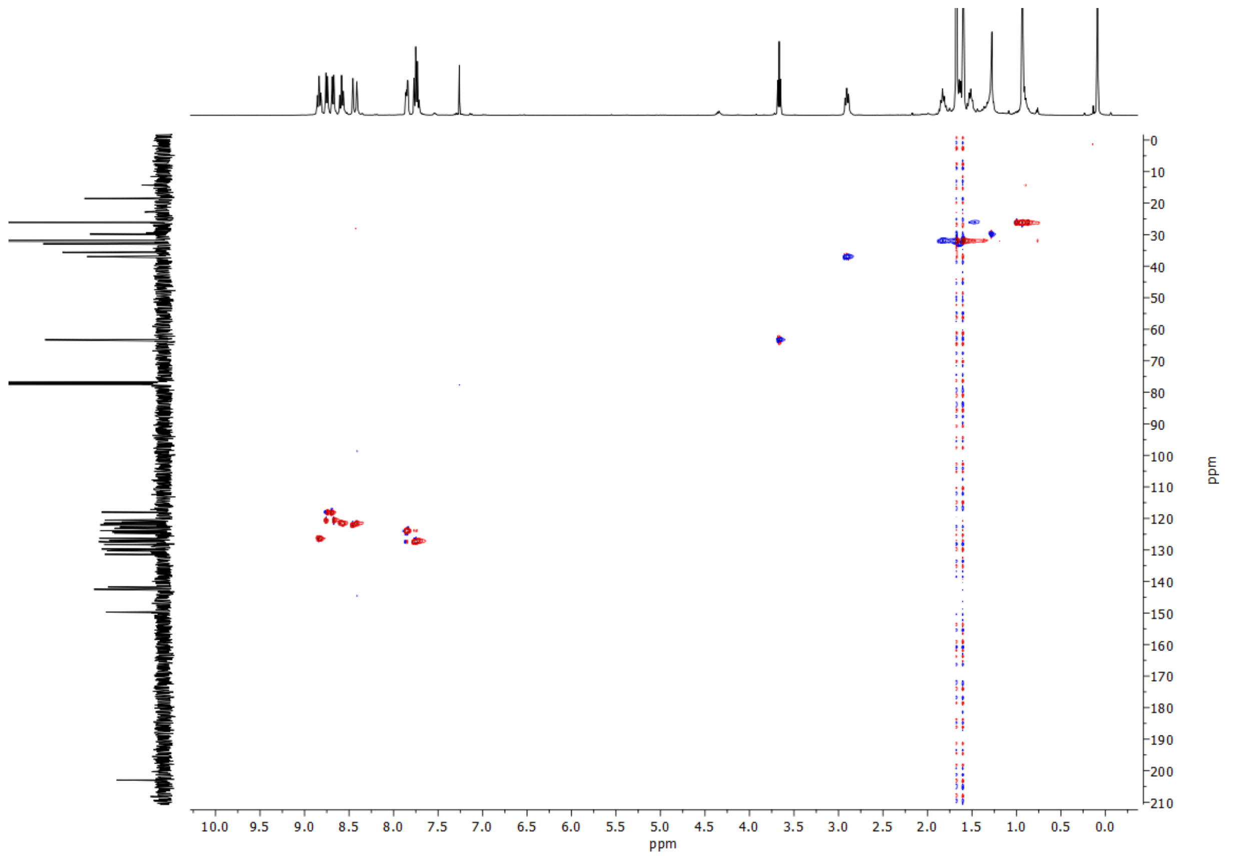

Figure S10. HSQC NMR (400 and 101 MHz, $CDCl_3$) spectrum of compound **10**.

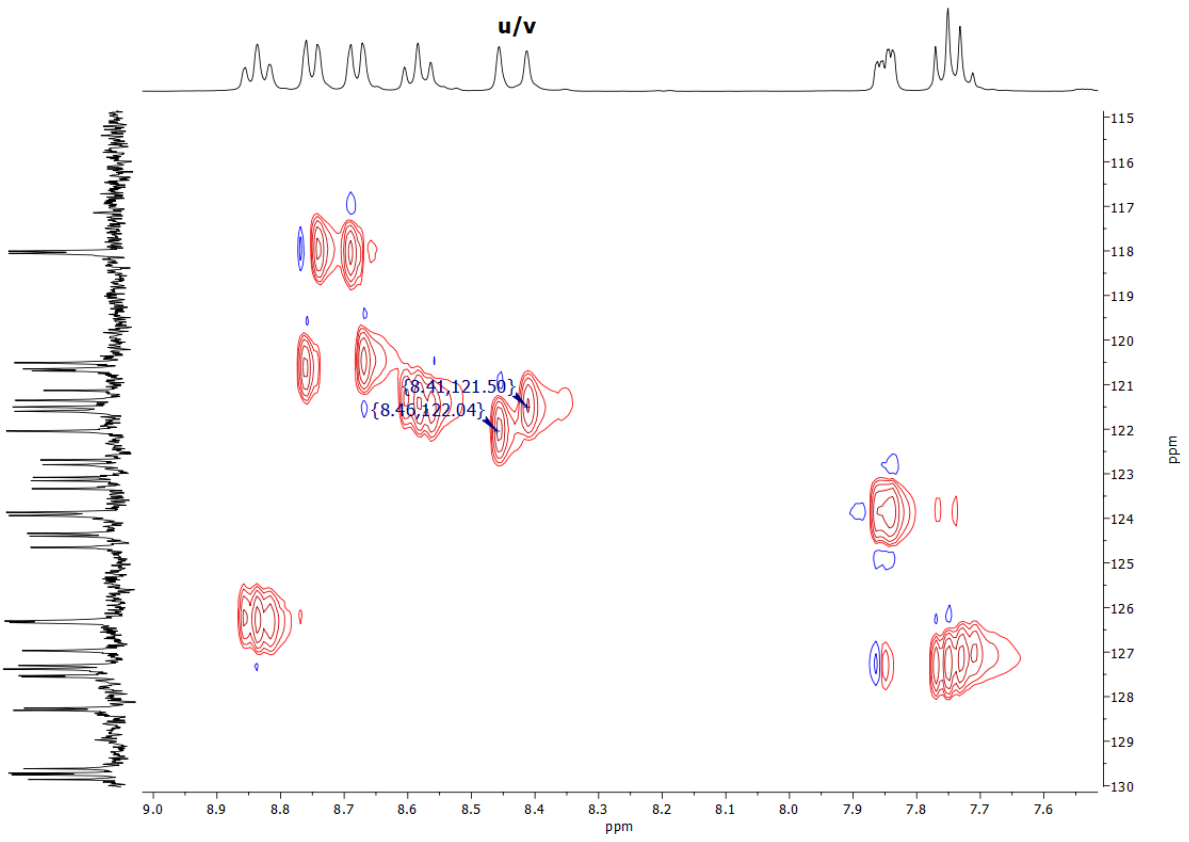

Figure S11. Partial HSQC NMR (400 and 101 MHz, $CDCl_3$) spectrum of compound **10**.

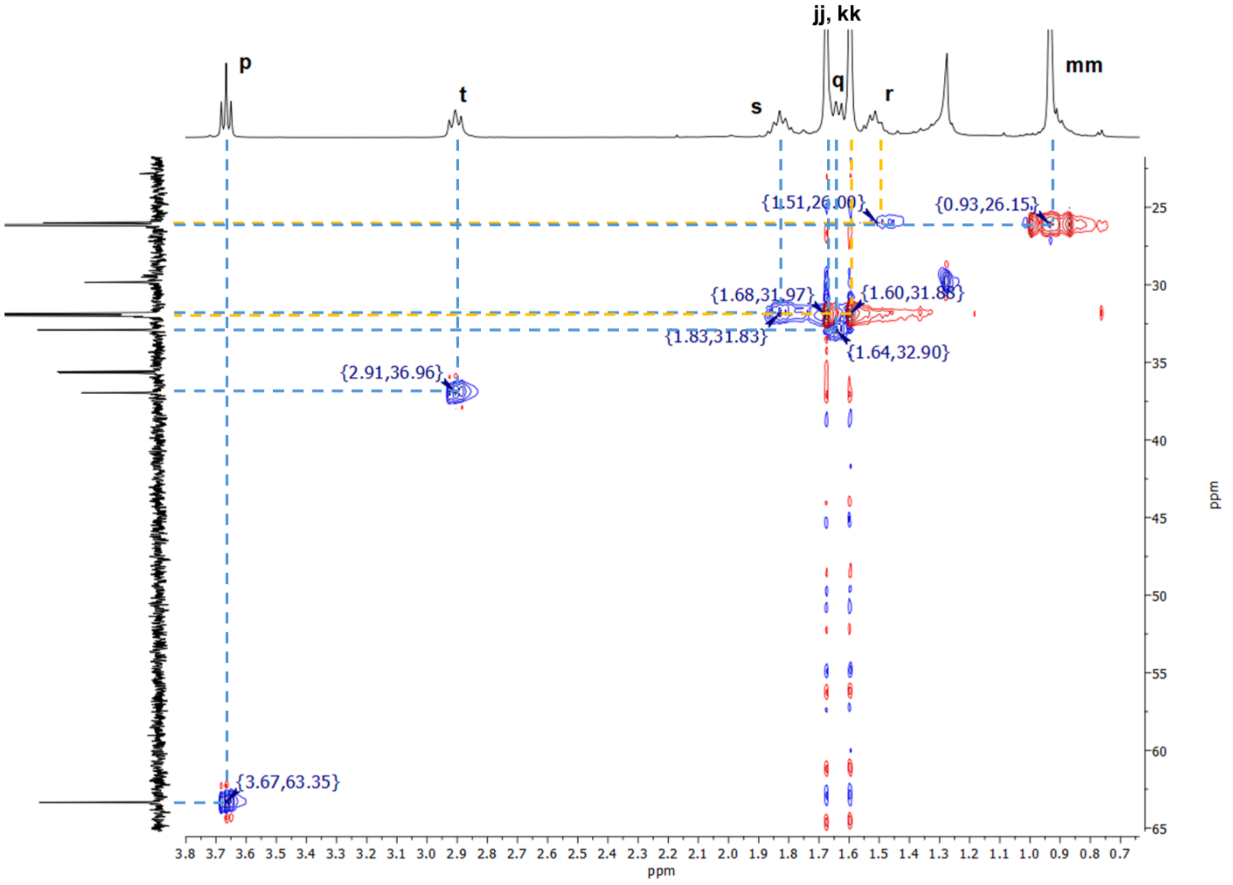

Figure S12. Partial HSQC NMR (400 and 101 MHz, $CDCl_3$) spectrum of compound **10**.

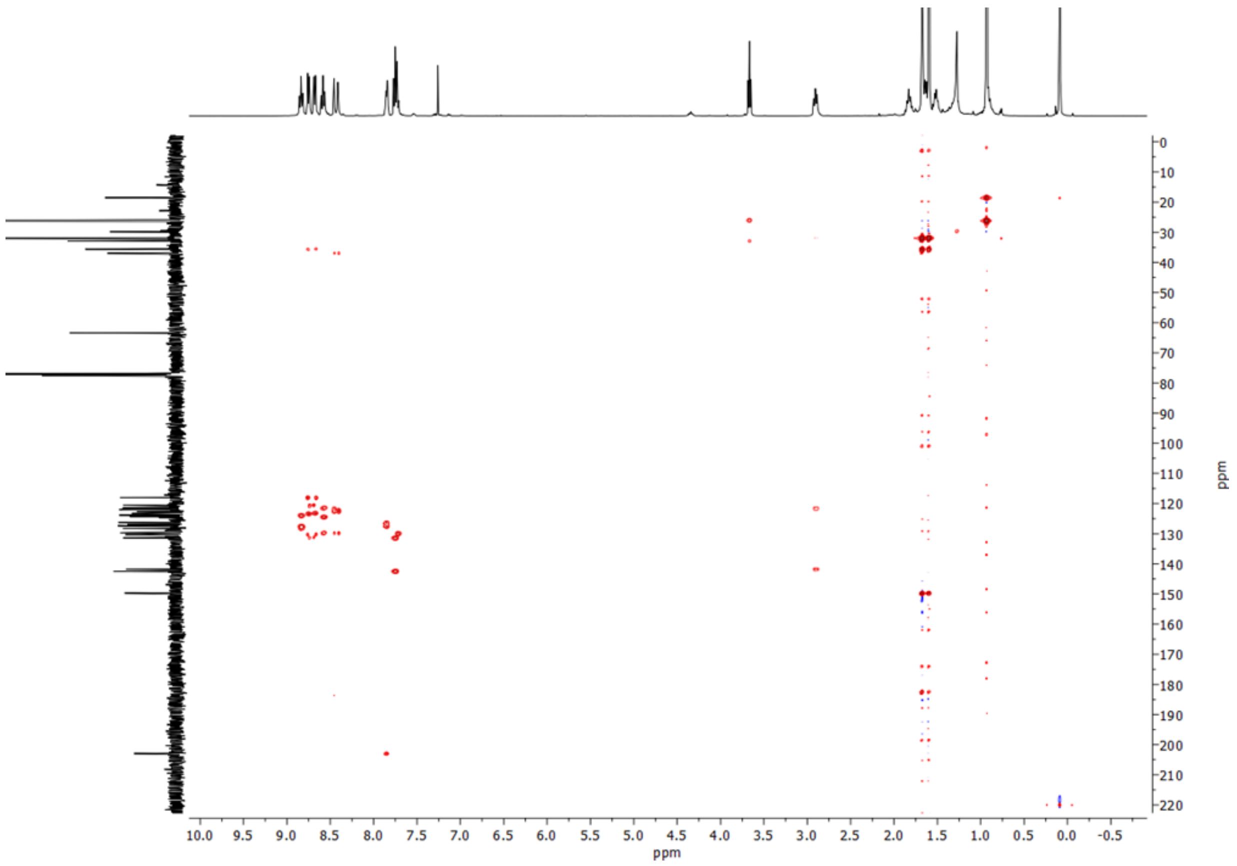

Figure S13. HMBC NMR (400 and 101 MHz, $CDCl_3$) spectrum of compound **10**.

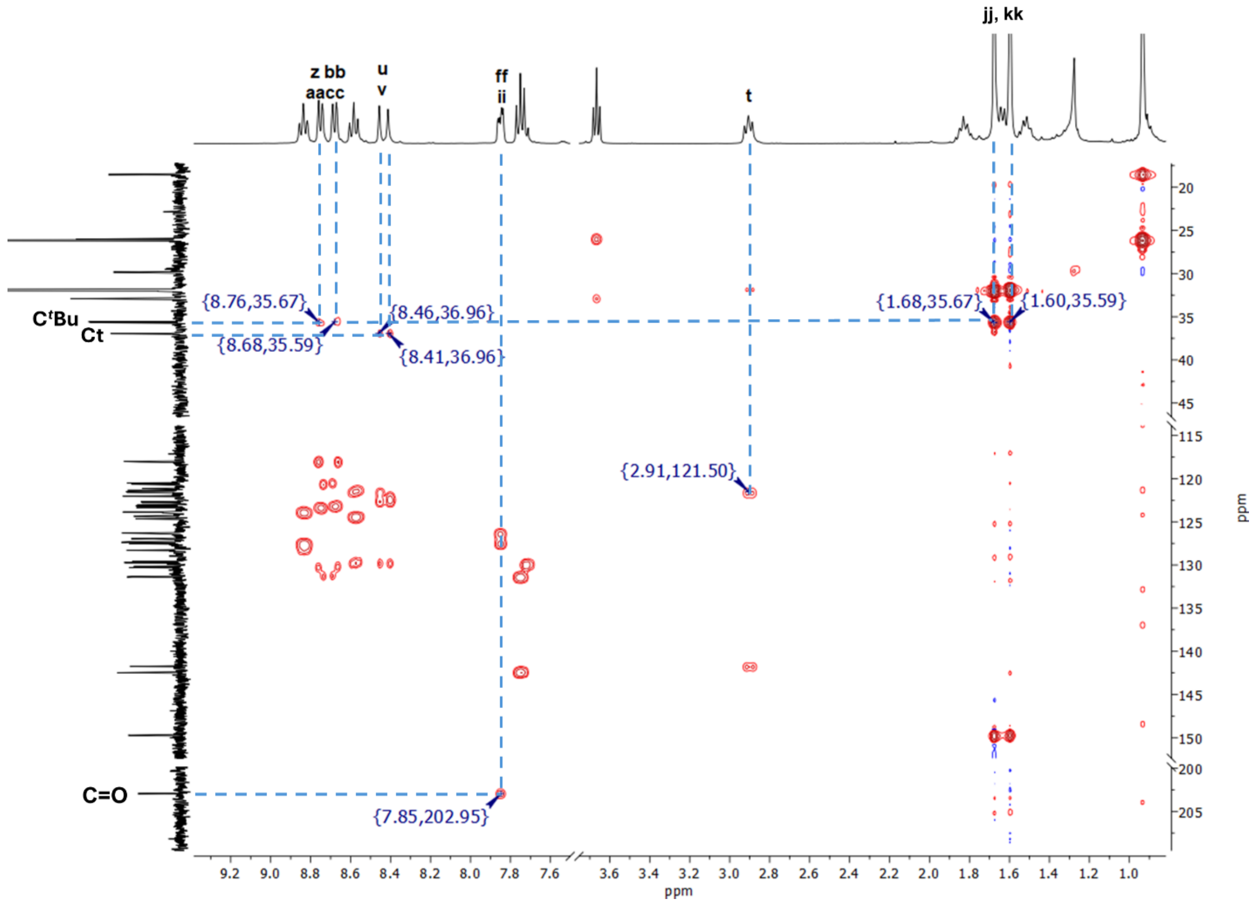

Figure S14. Partial HMBC NMR (400 and 101 MHz, $CDCl_3$) spectrum of compound **10**.

Compound 11

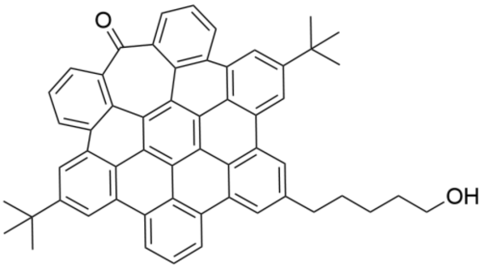

11

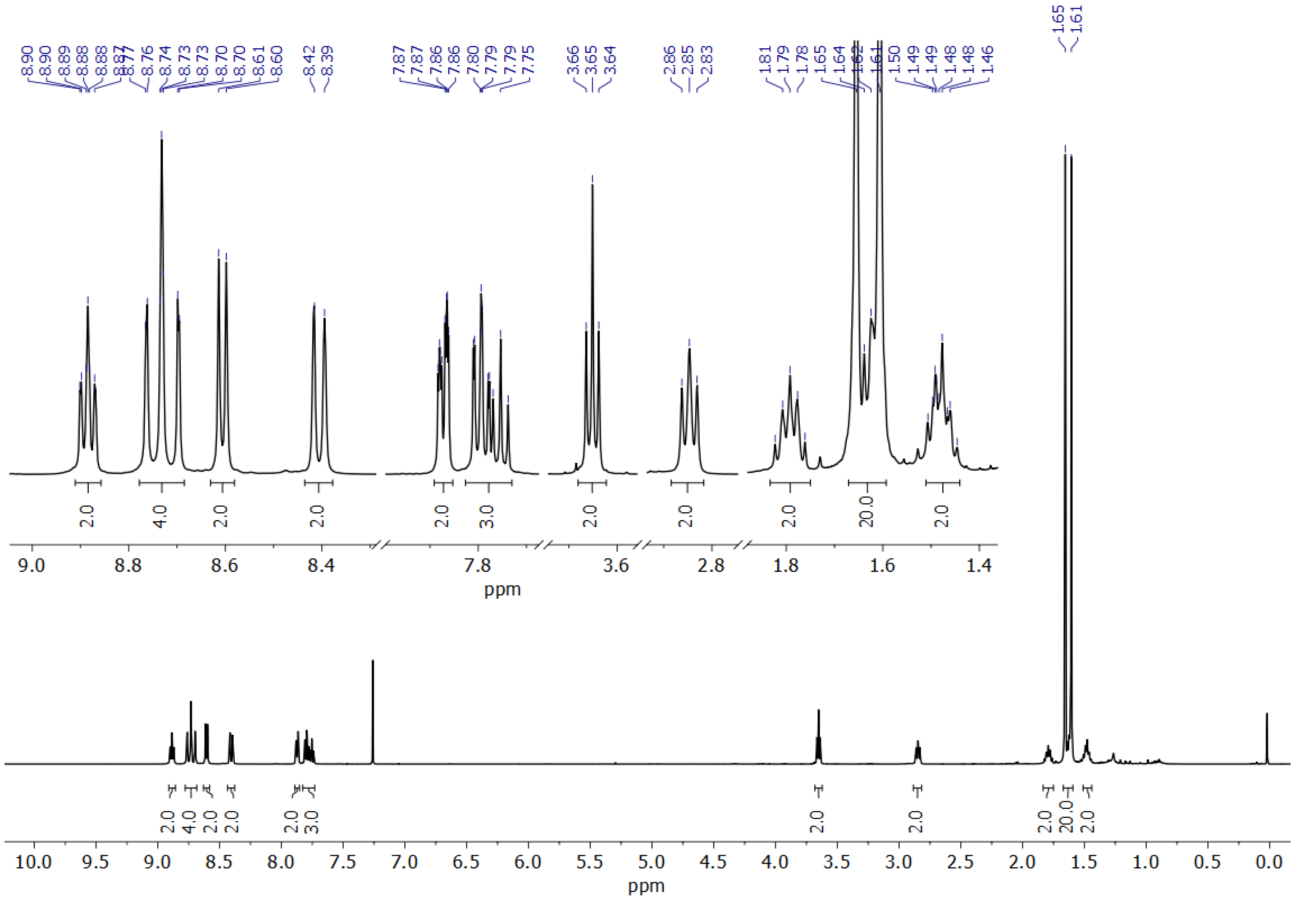

Figure S15. ^{1}H NMR (500 MHz, $CDCl_3$) spectrum of compound **11**.

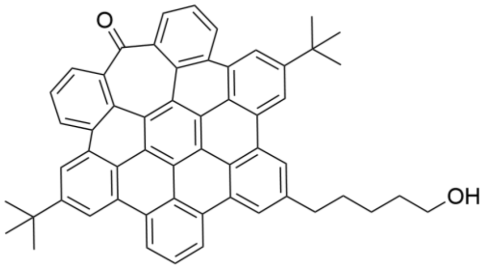

11

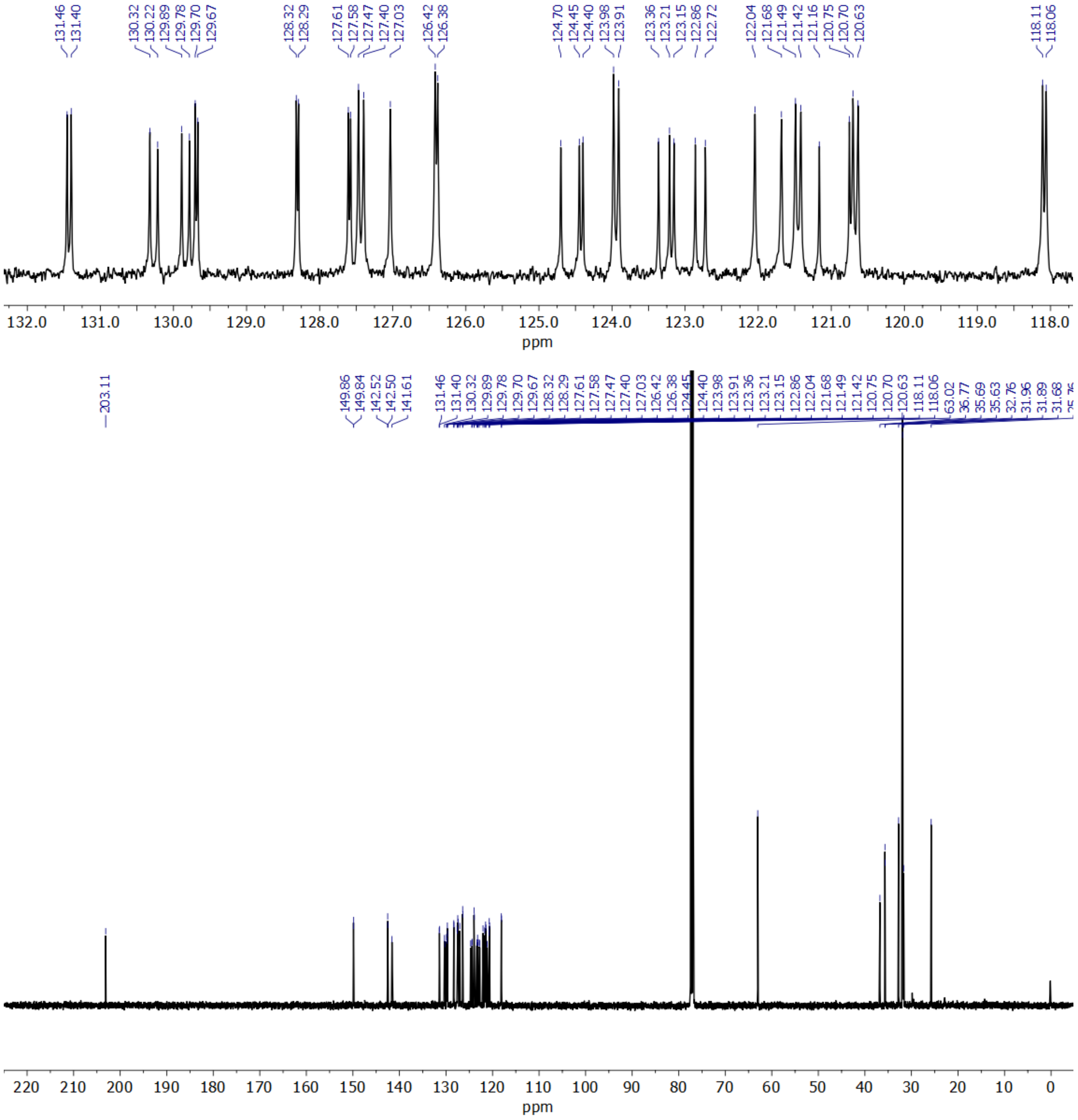

Figure S16. $^{13}C\{^{1}H\}$ NMR (126 MHz, $CDCl_3$) spectrum of compound **11**.

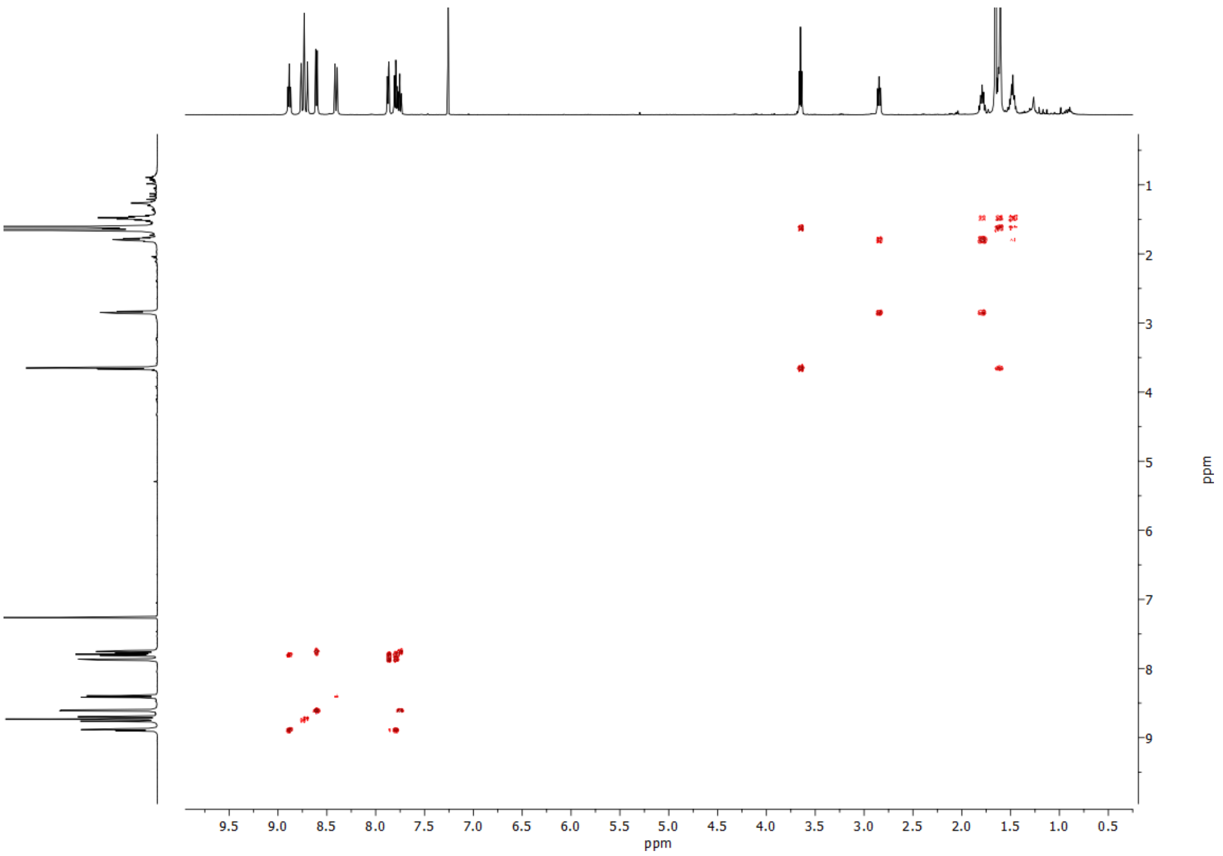

Figure S17. COSY NMR (500 MHz, $CDCl_3$) spectrum of compound **11**.

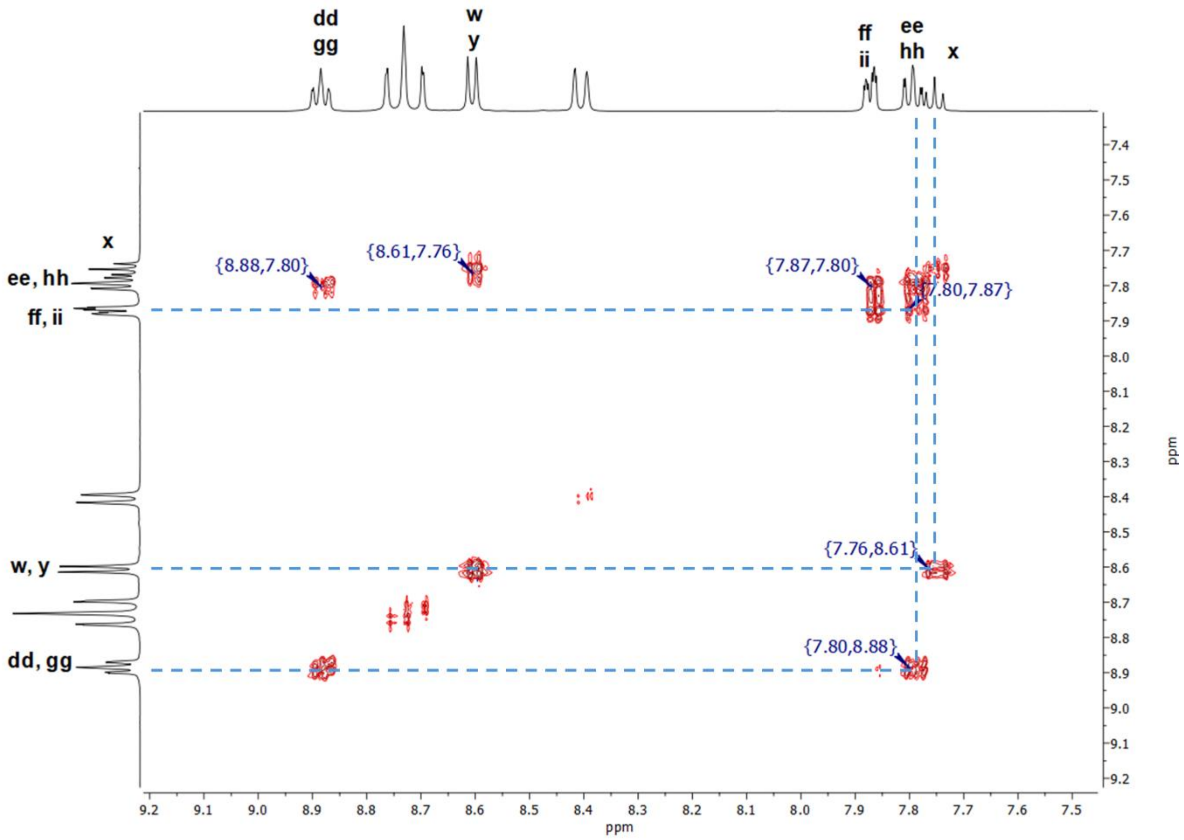

Figure S18. Partial COSY NMR (500 MHz, $CDCl_3$) spectrum of compound **11**.

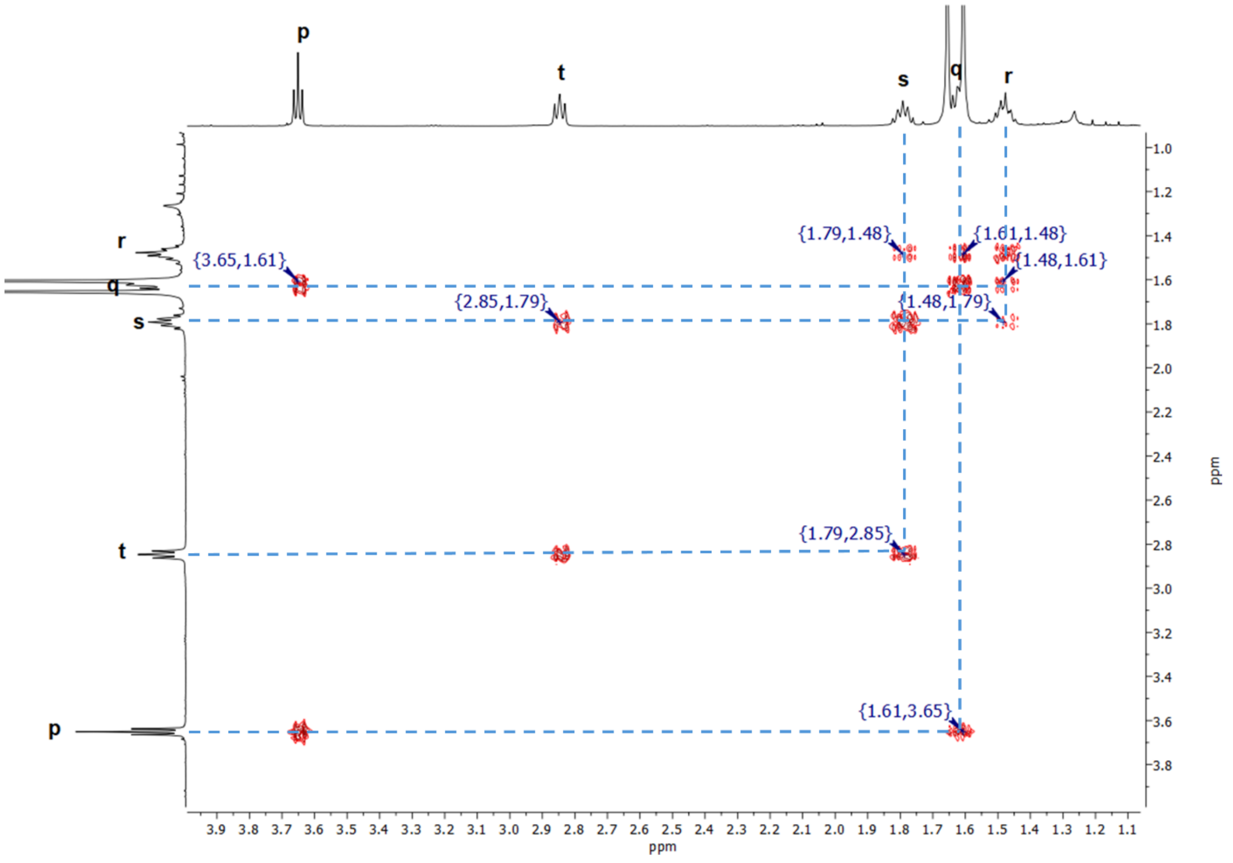

Figure S19. Partial COSY NMR (500 MHz, $CDCl_3$) spectrum of compound **11**.

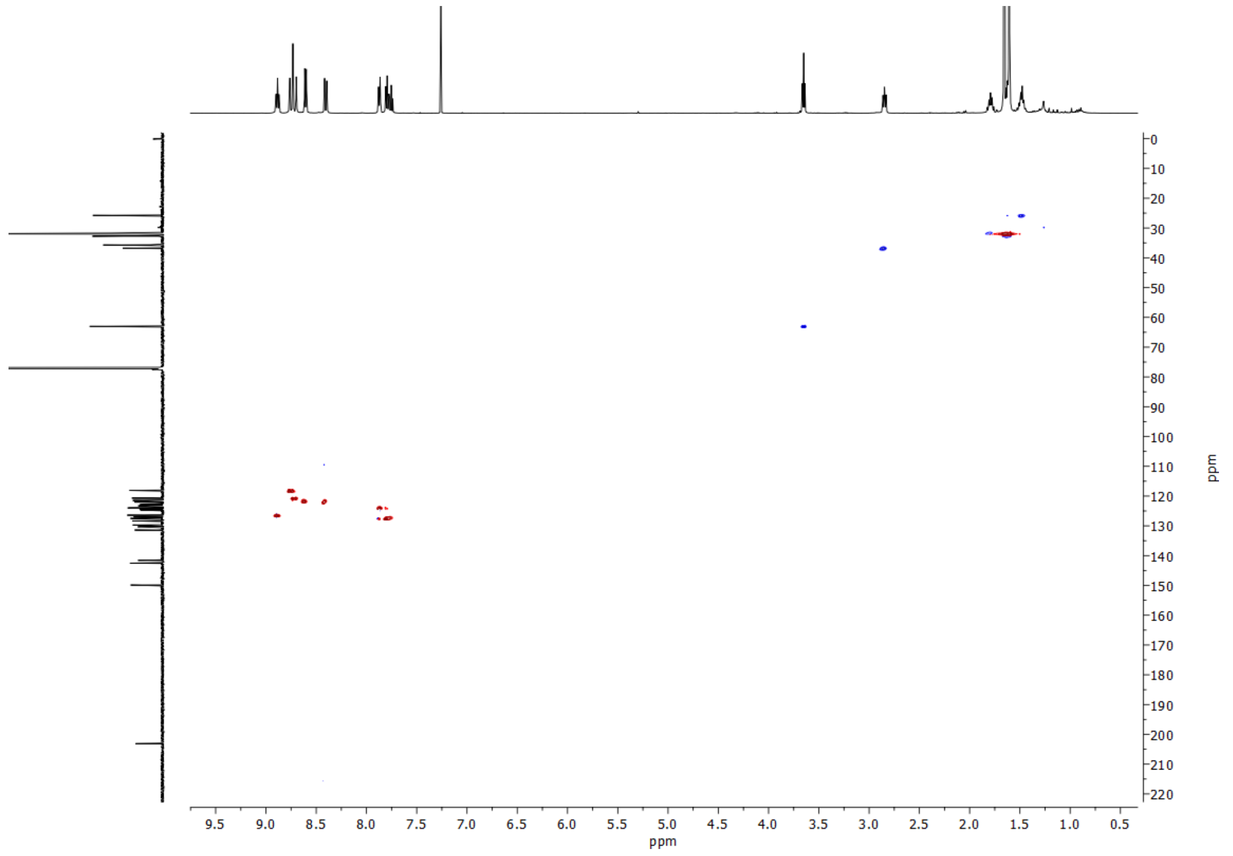

Figure S20. HSQC NMR (500 and 126 MHz, $CDCl_3$) spectrum of compound **11**.

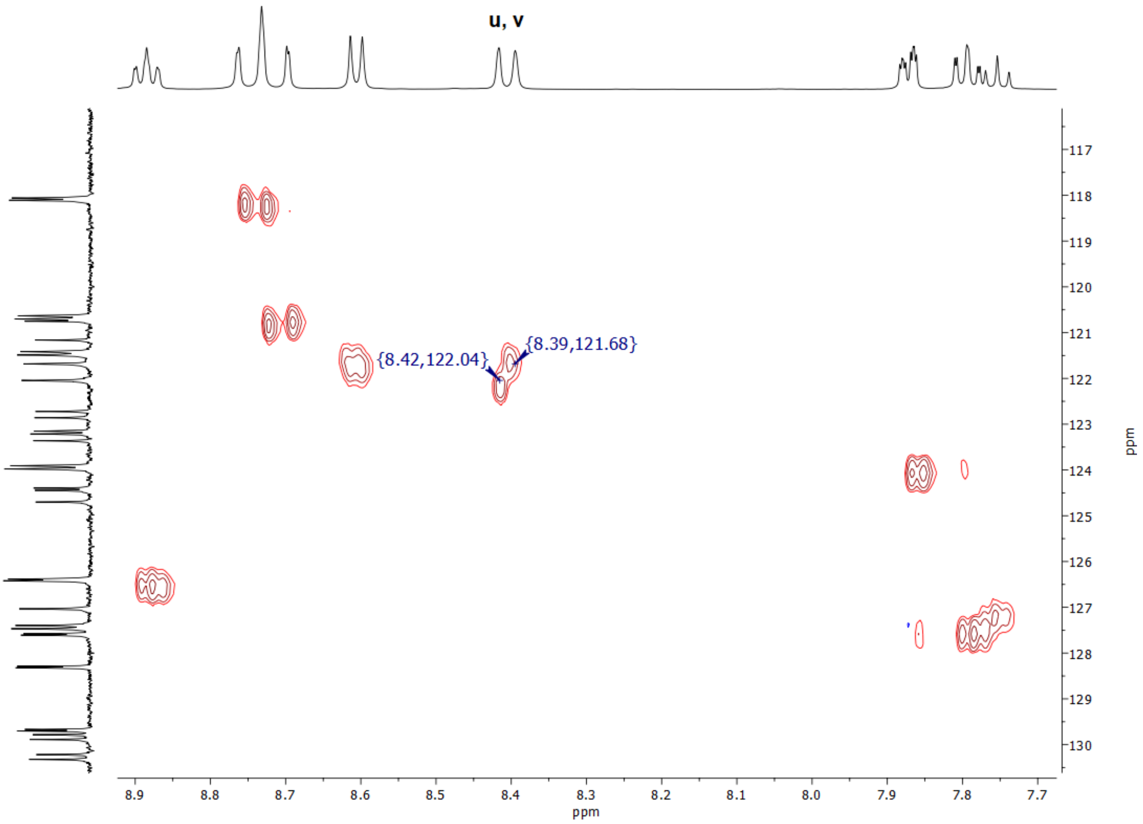

Figure S21. Partial HSQC NMR (500 and 126 MHz, $CDCl_3$) spectrum of compound **11**.

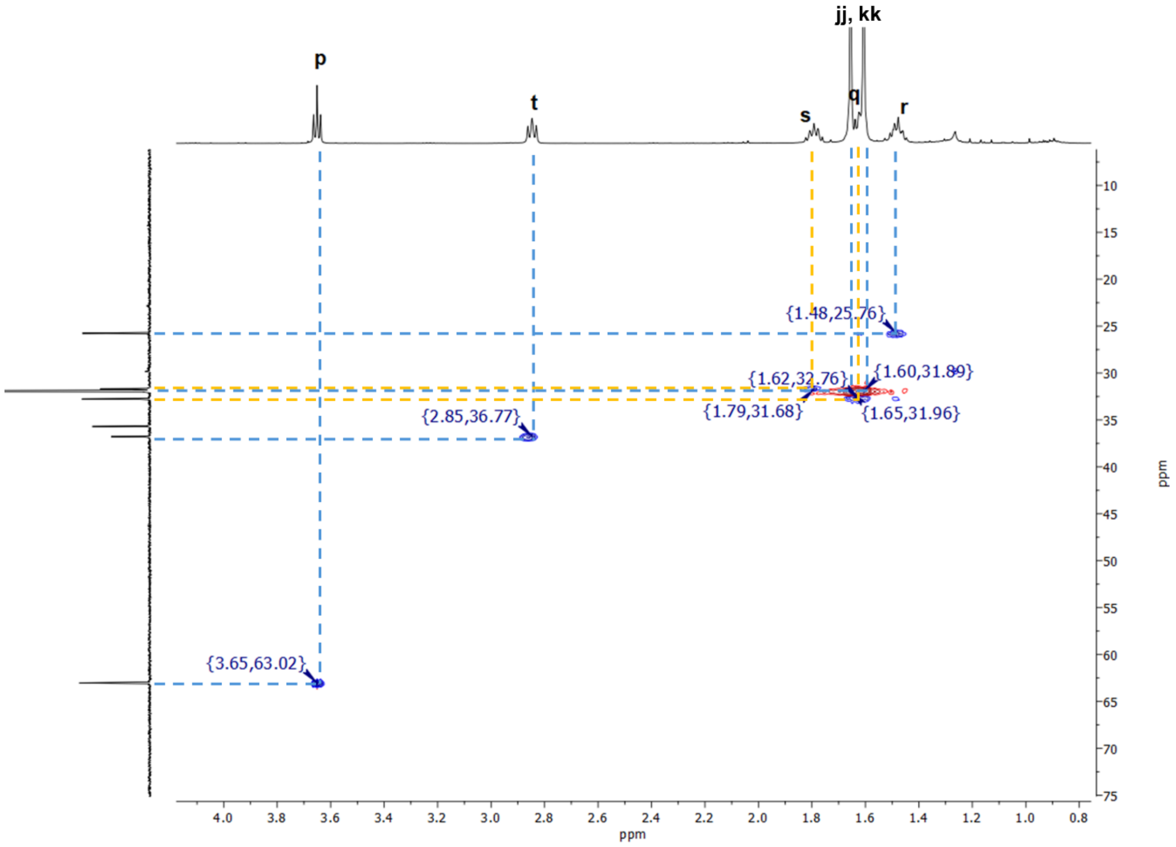

Figure S22. Partial HSQC NMR (500 and 126 MHz, $CDCl_3$) spectrum of compound **11**.

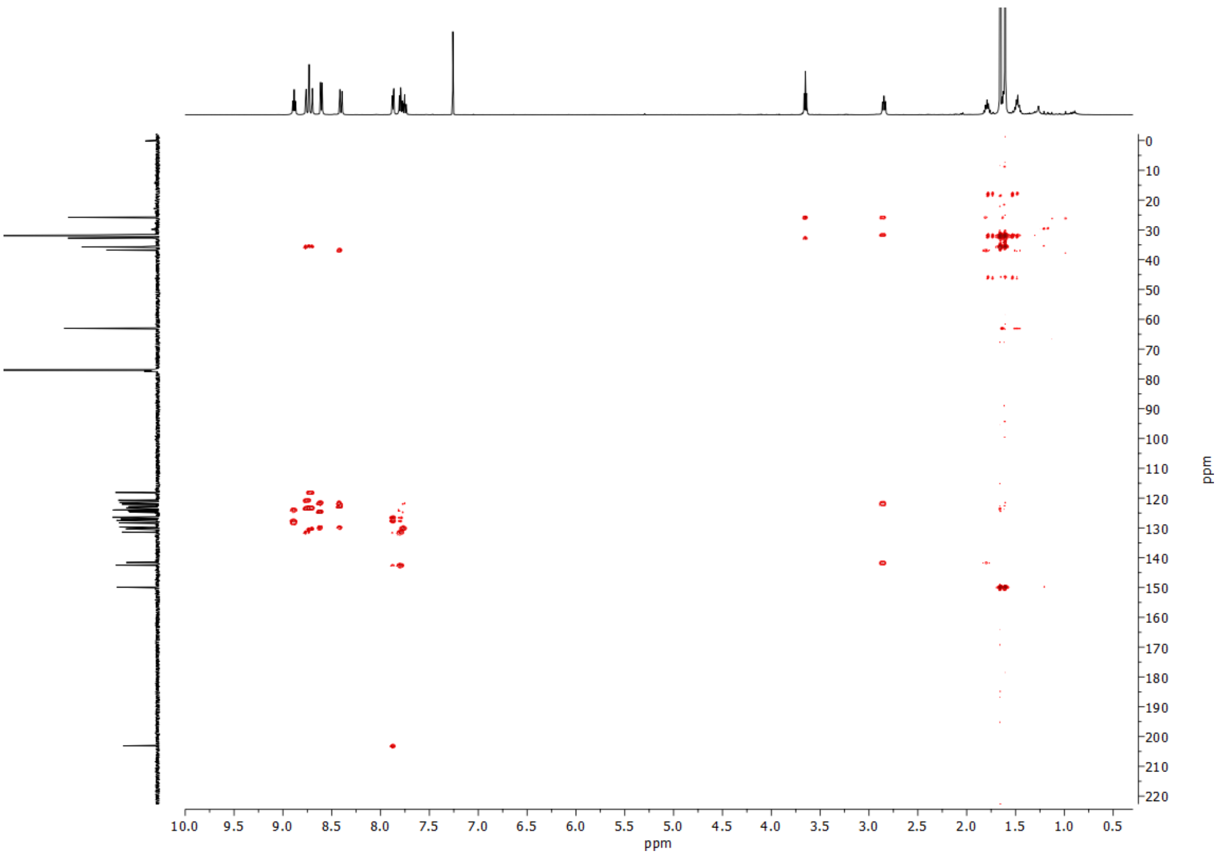

Figure S23. HMBC NMR (500 and 126 MHz, $CDCl_3$) spectrum of compound **11**.

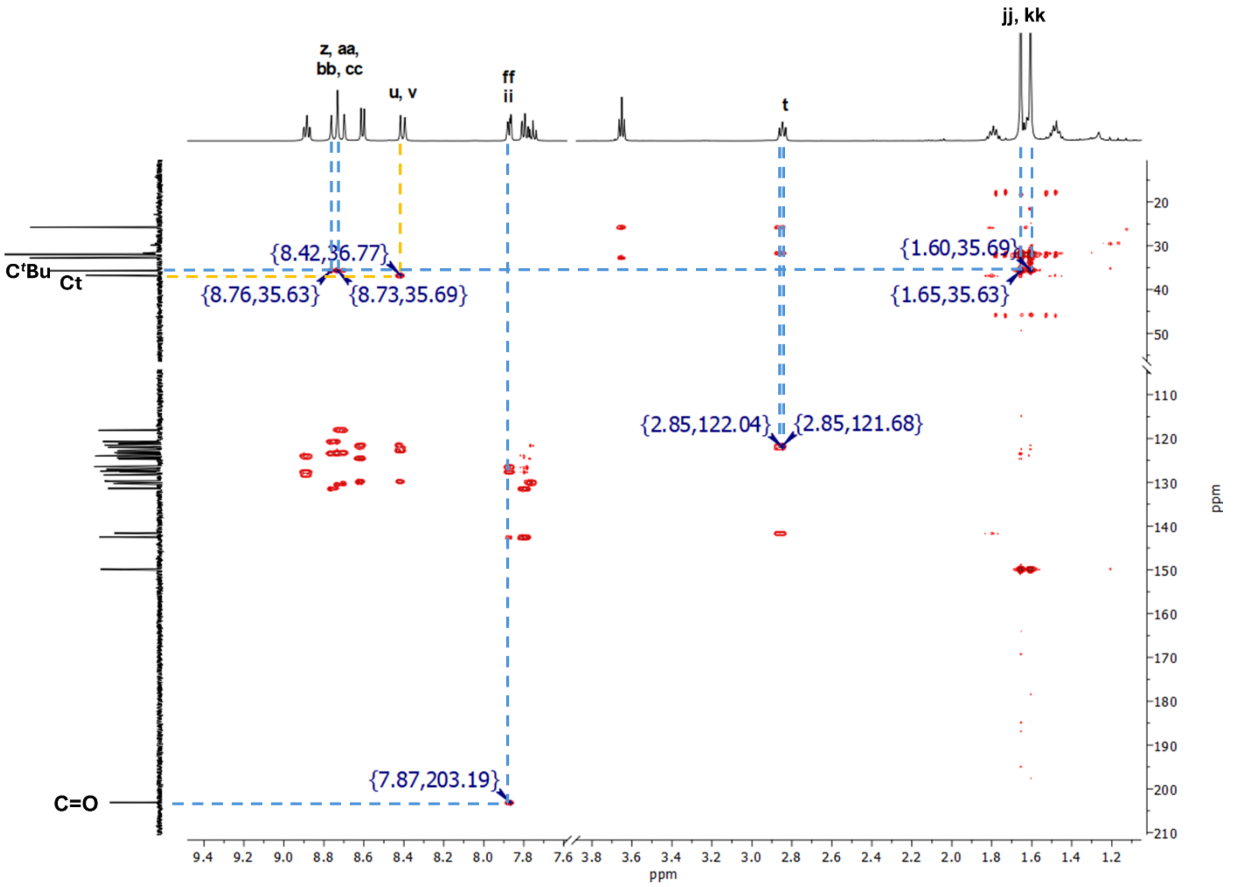

Figure S24. Partial HMBC NMR (500 and 126 MHz, $CDCl_3$) spectrum of compound **11**.

Compound 3

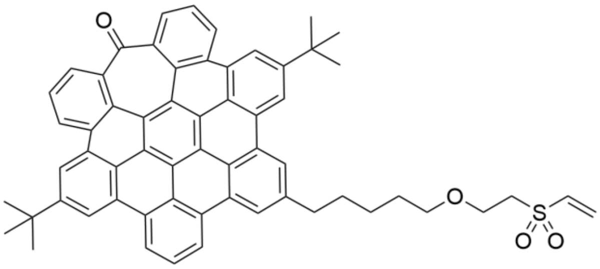

3

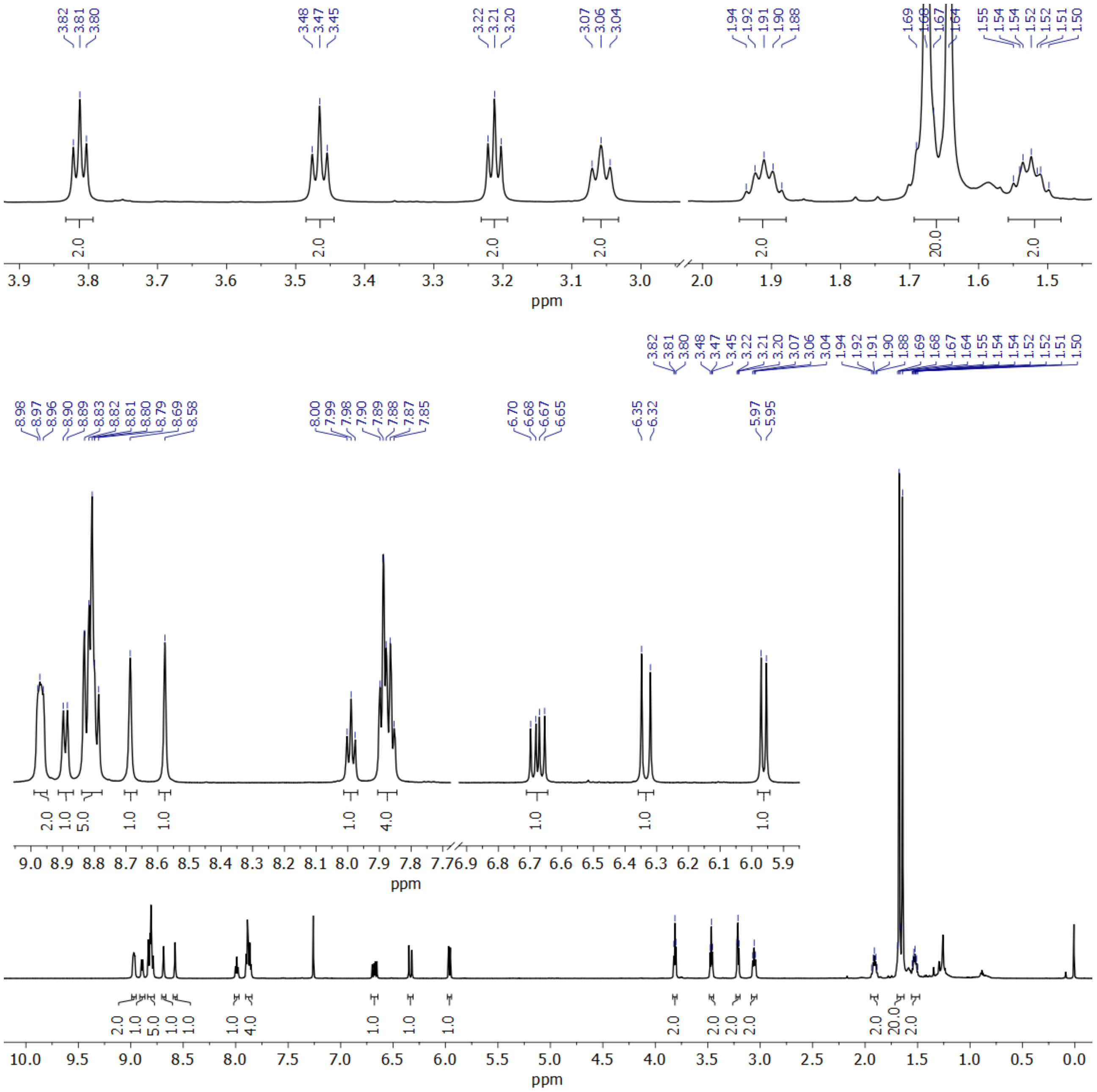

Figure S25. ^{1}H NMR (600 MHz, $CDCl_3$) spectrum of compound **3**.

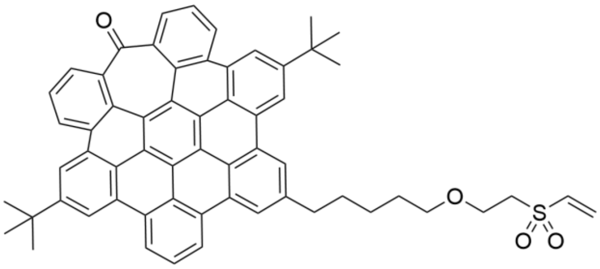

3

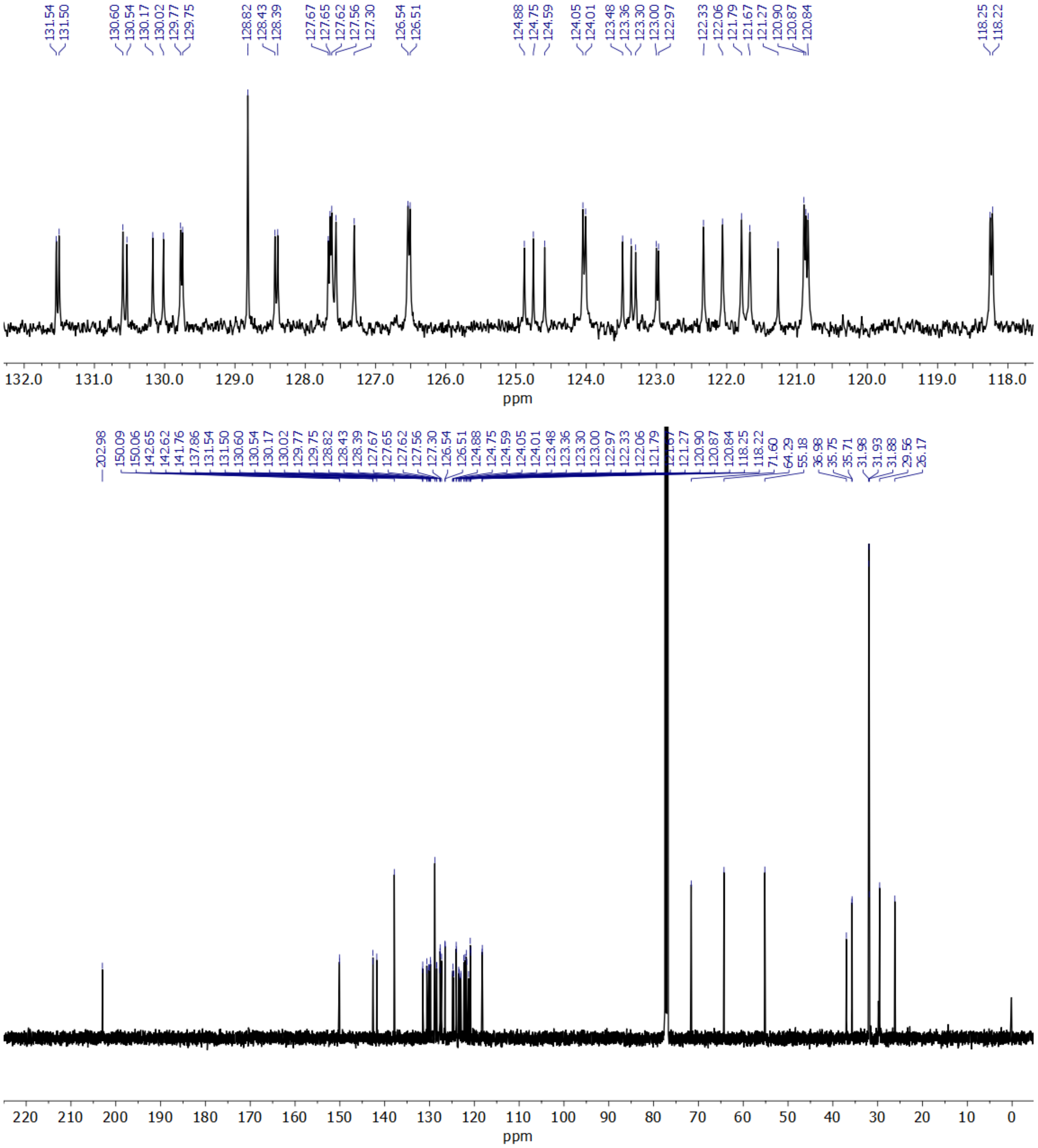

Figure S26. $^{13}C\{^{1}H\}$ NMR (126 MHz, $CDCl_3$) spectrum of compound **3**.

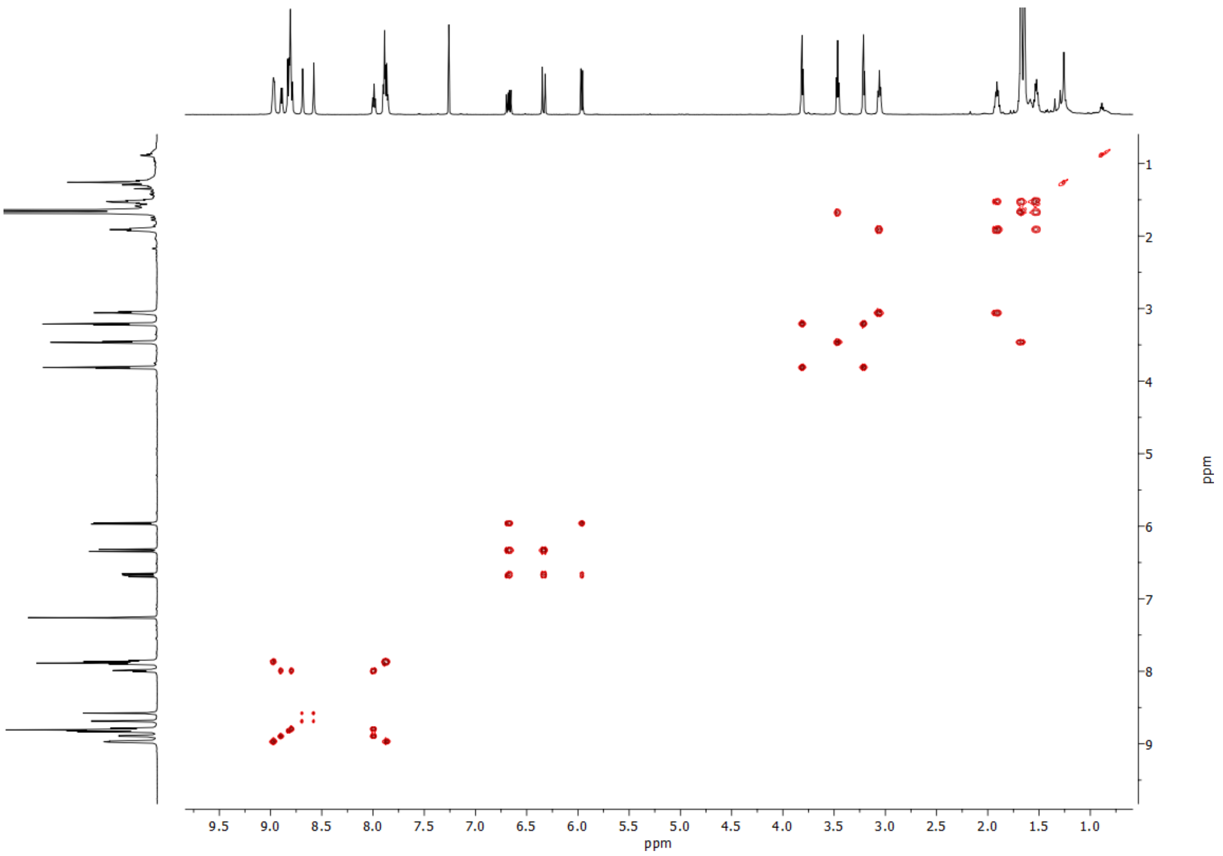

Figure S27. COSY NMR (600 MHz, $CDCl_3$) spectrum of compound **3**.

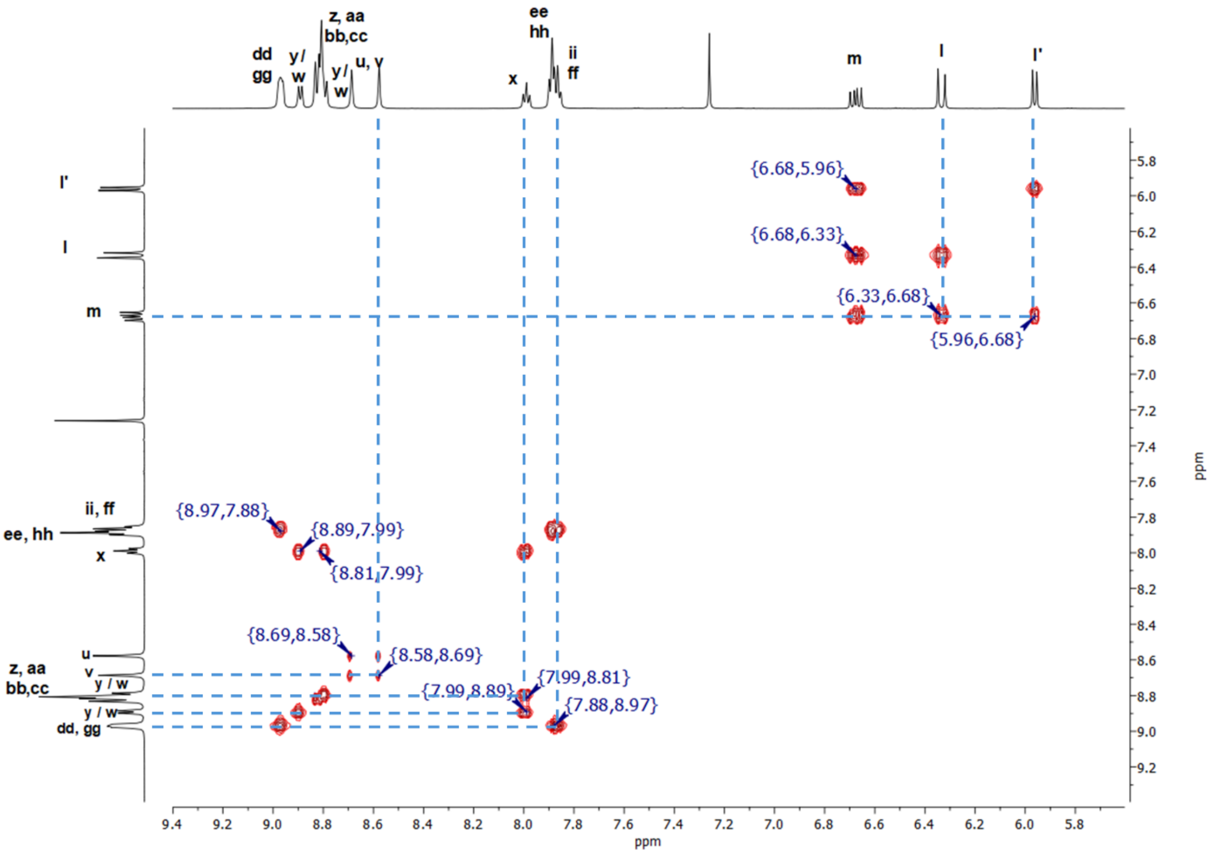

Figure S28. Partial COSY NMR (600 MHz, $CDCl_3$) spectrum of compound **3**.

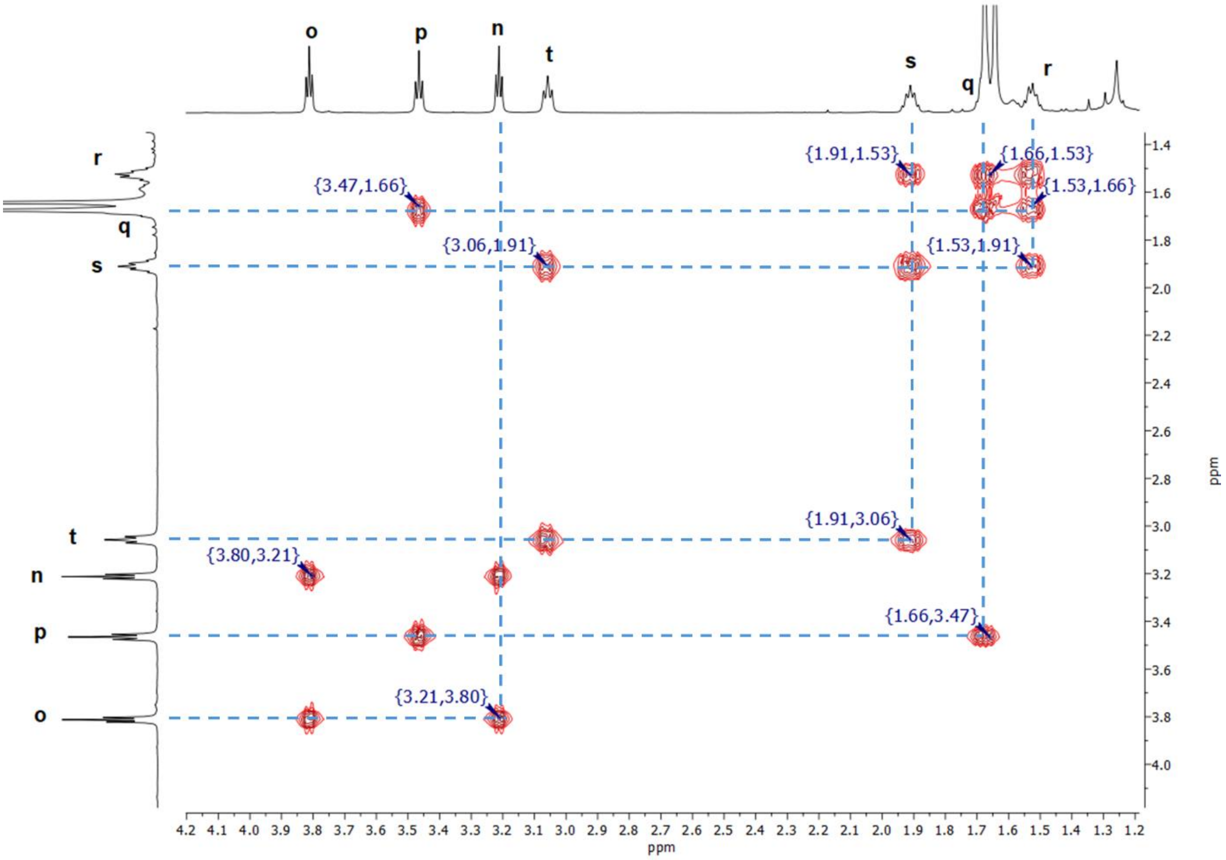

Figure S29. Partial COSY NMR (600 MHz, $CDCl_3$) spectrum of compound **3**.

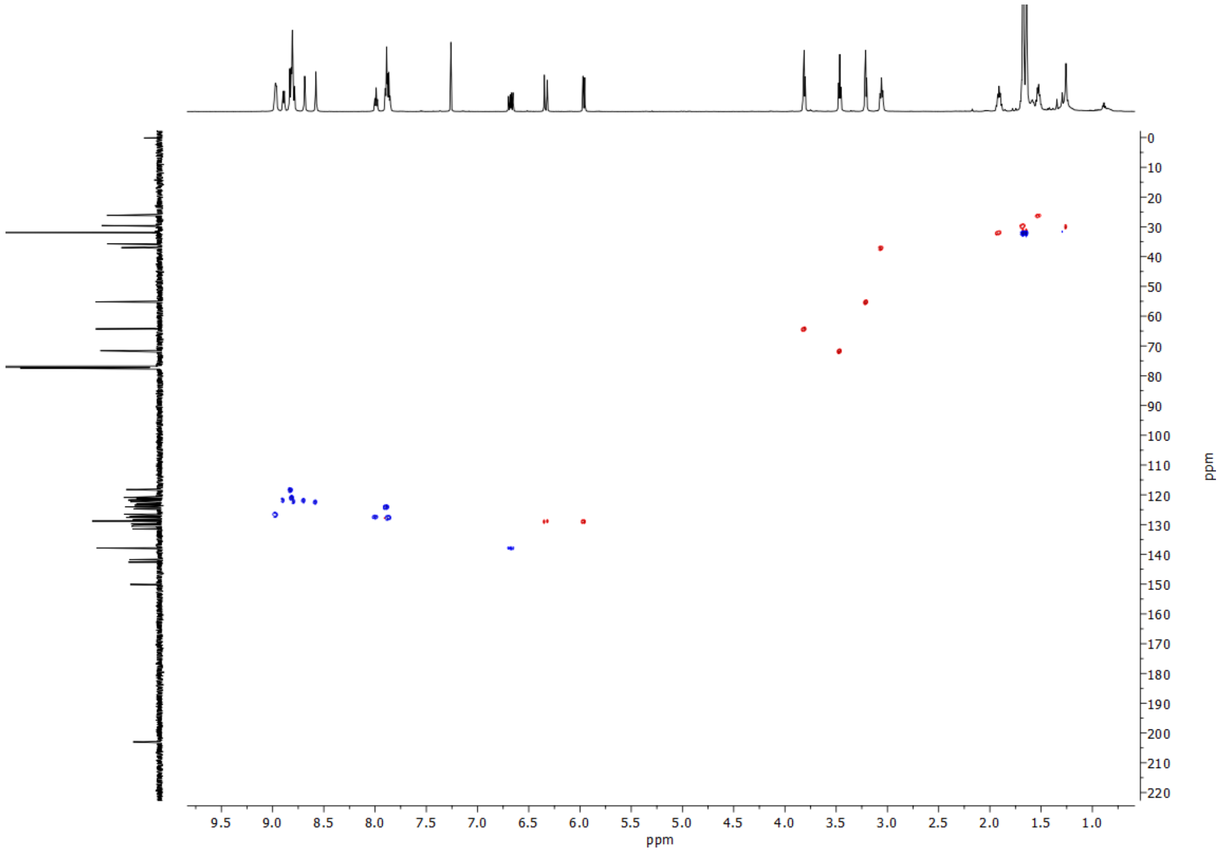

Figure S30. HSQC NMR (600 and 151 MHz, $CDCl_3$) spectrum of compound **3**.

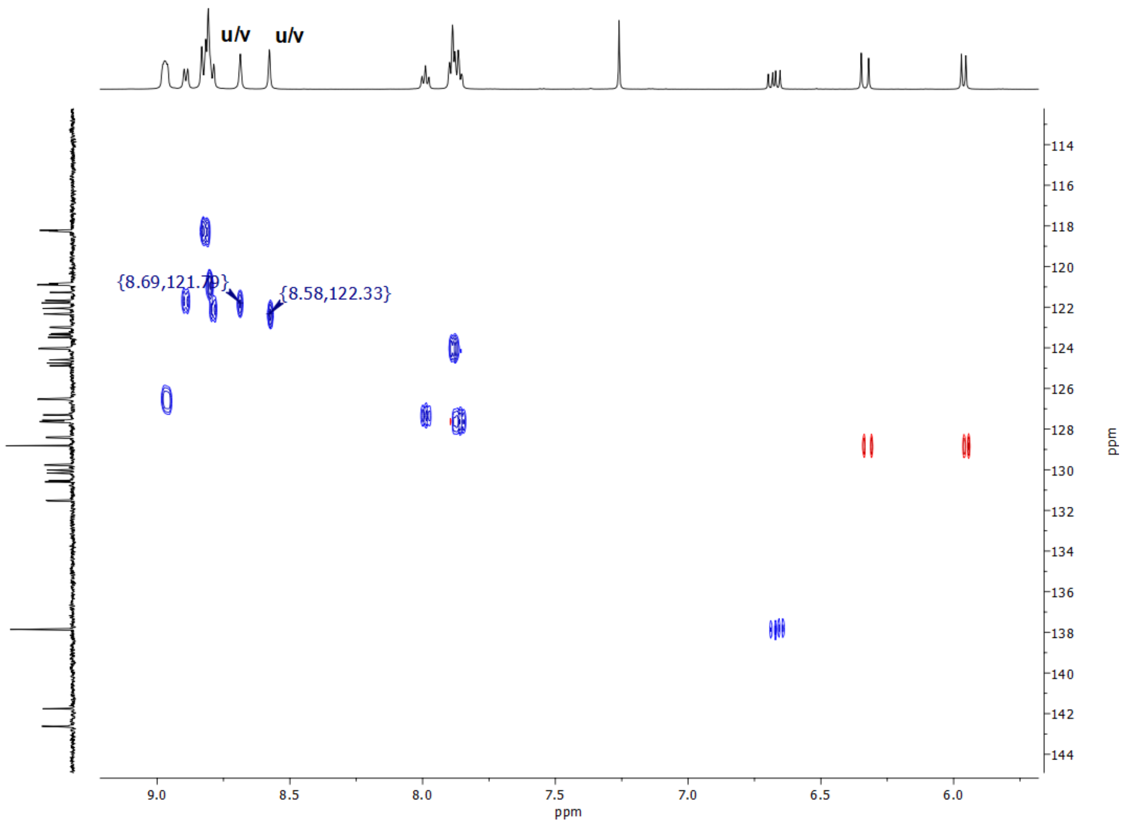

Figure S31. Partial HSQC NMR (600 and 151 MHz, $CDCl_3$) spectrum of compound **3**.

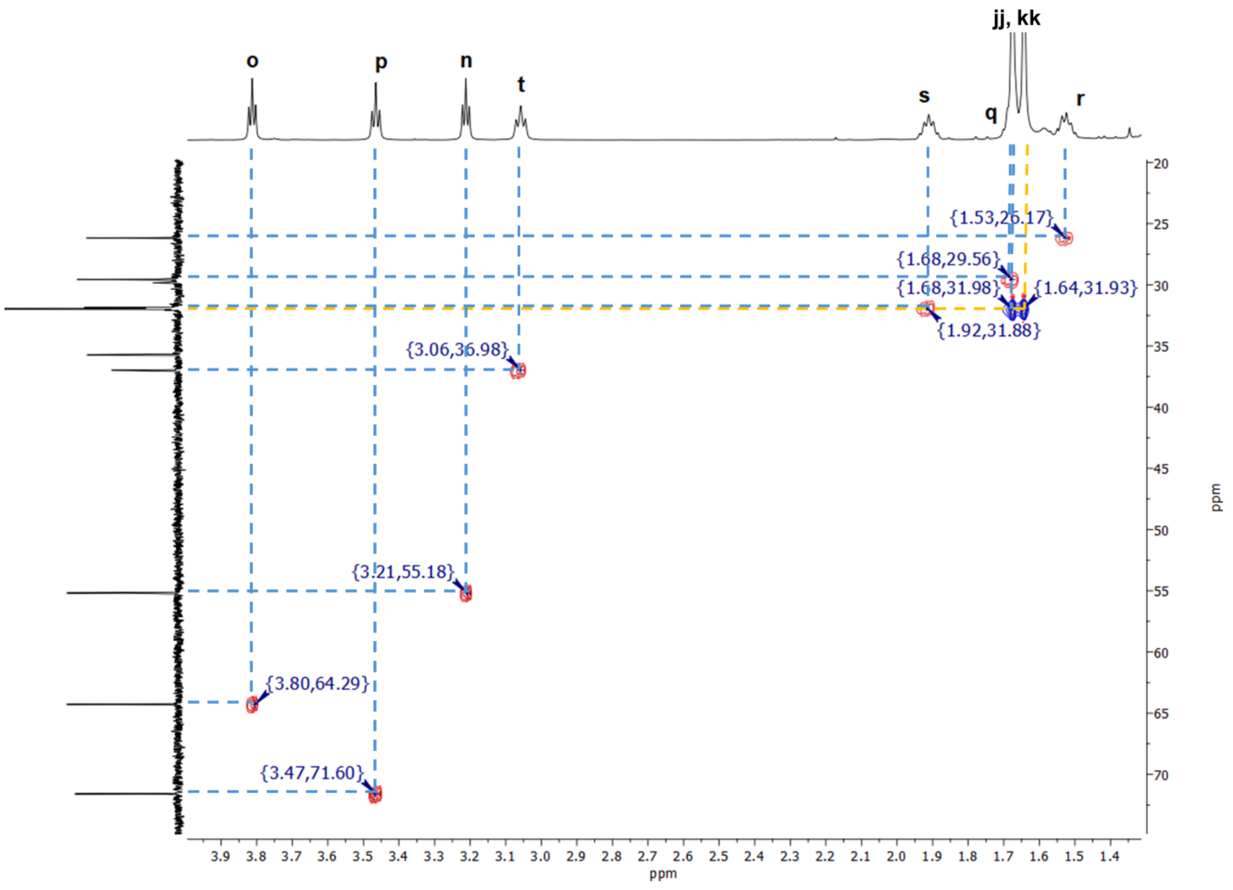

Figure S32. Partial HSQC NMR (600 and 151 MHz, $CDCl_3$) spectrum of compound **3**.

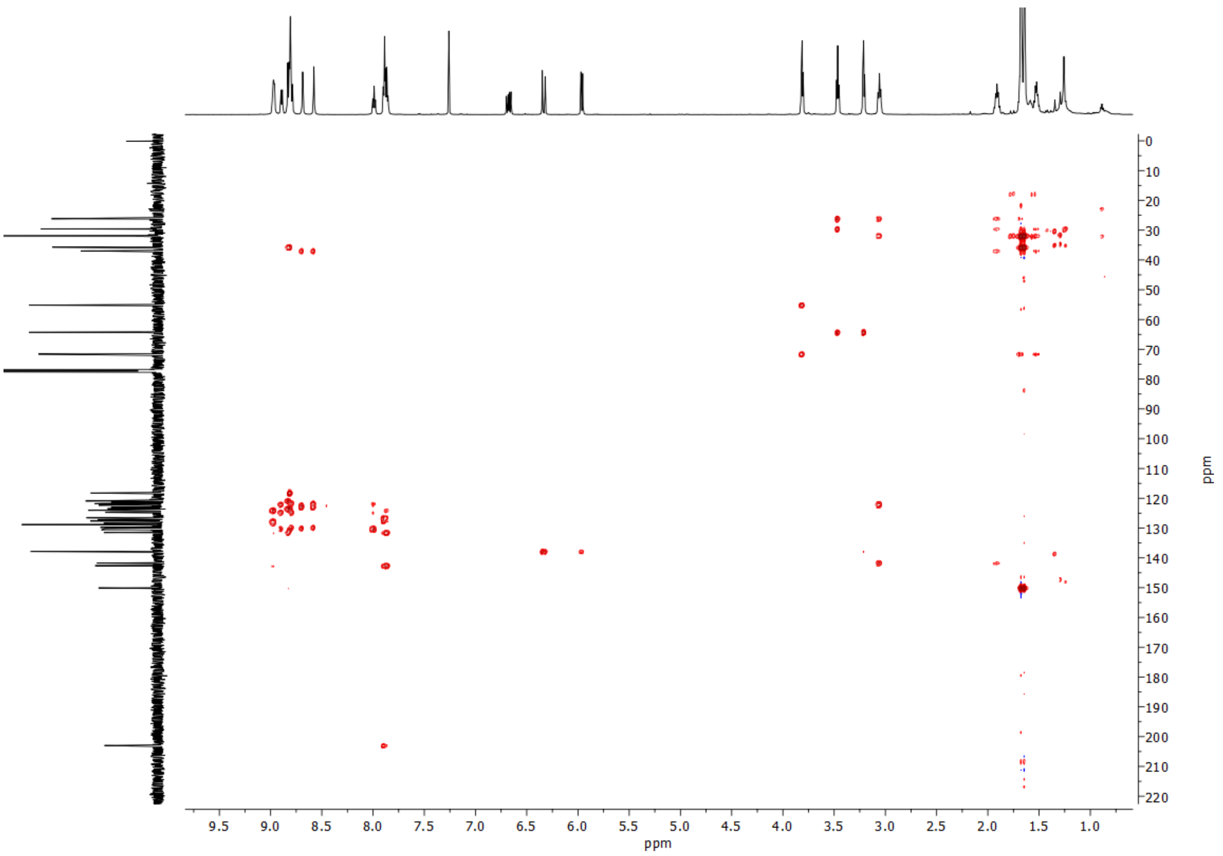

Figure S33. HMBC NMR (600 and 151 MHz, $CDCl_3$) spectrum of compound **3**.

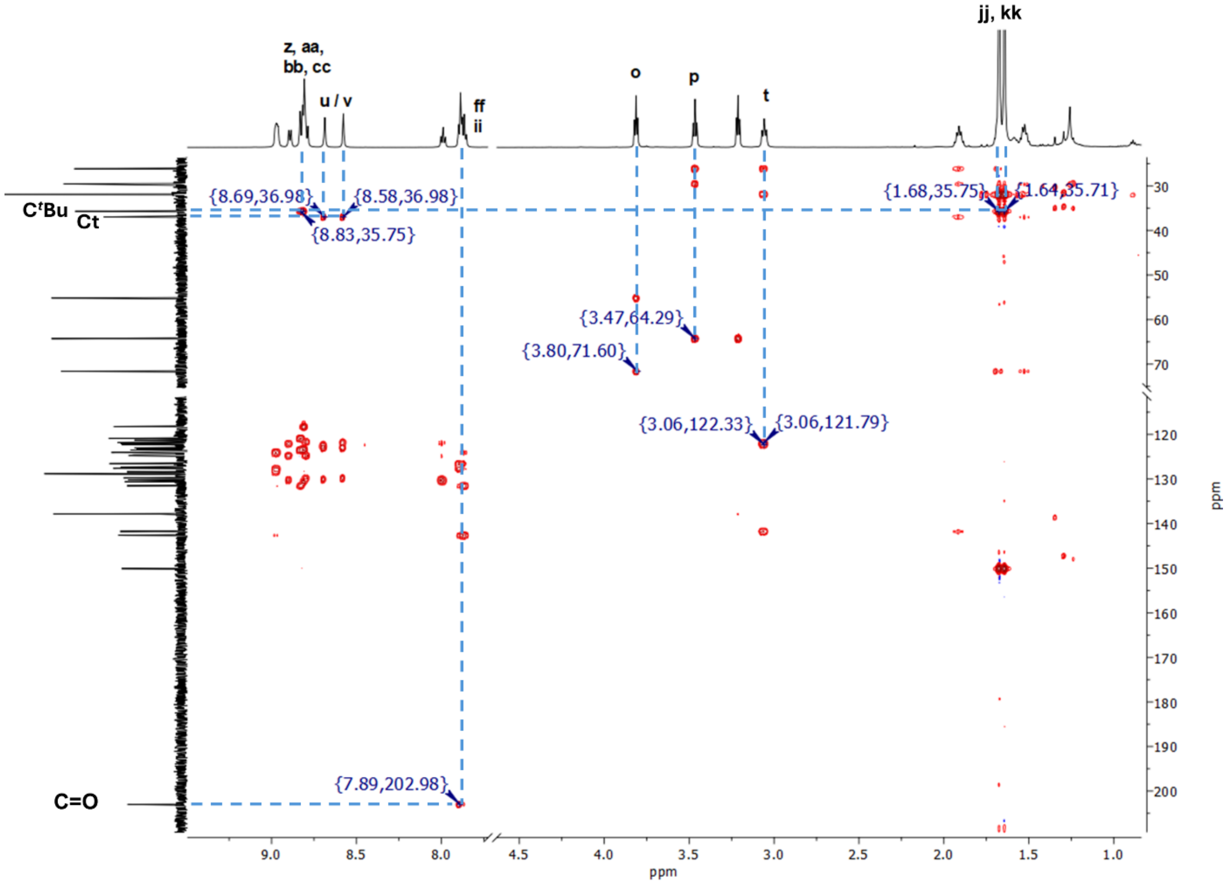

Figure S34. Partial HMBC NMR (600 and 151 MHz, $CDCl_3$) spectrum of compound **3**.

Compound 14

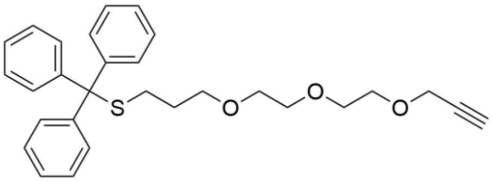

14

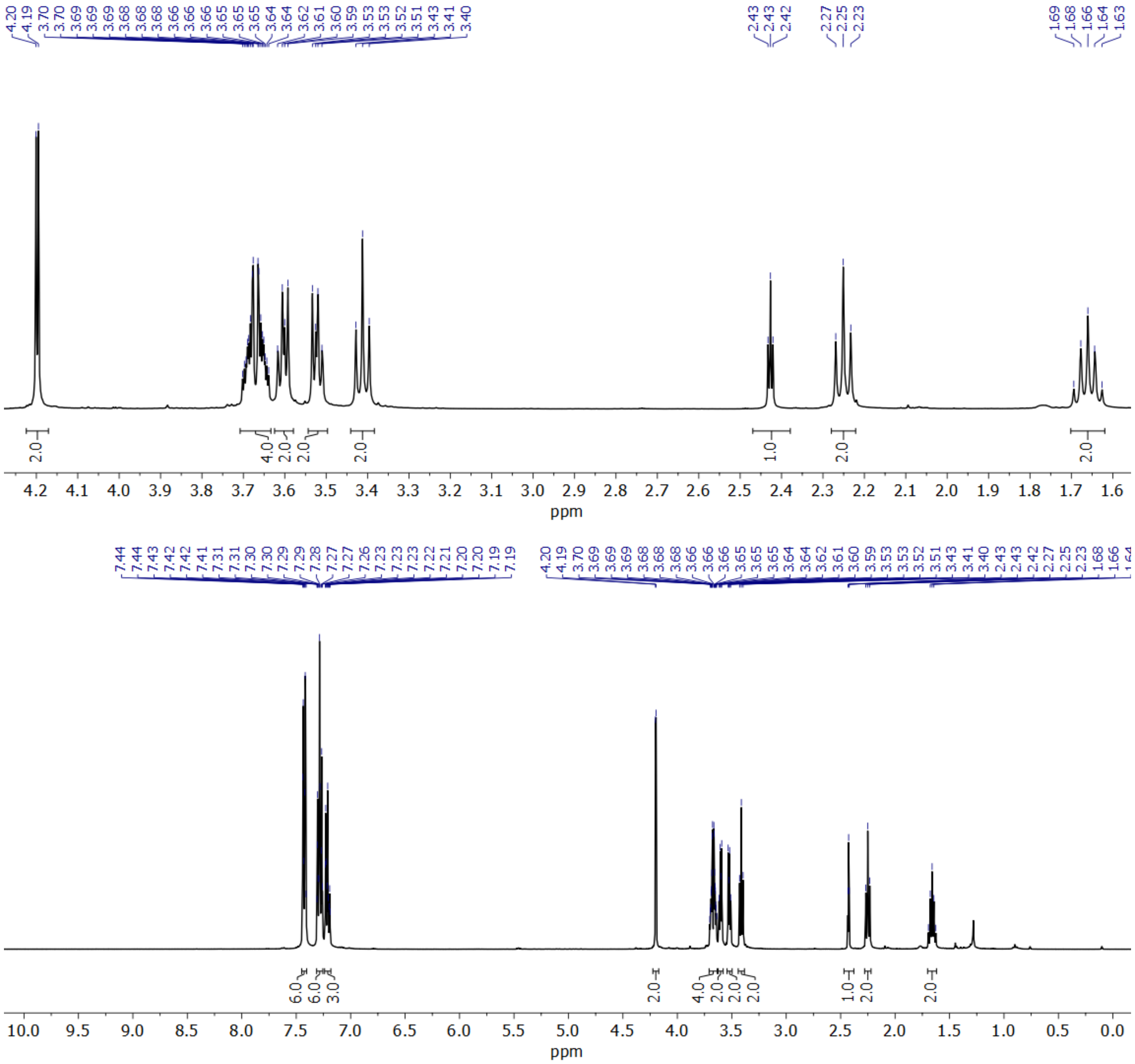

Figure S35. ^{1}H NMR (400 MHz, CD_2Cl_2) spectrum of compound **14**.

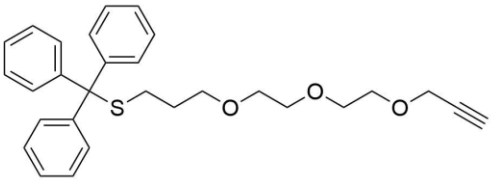

14

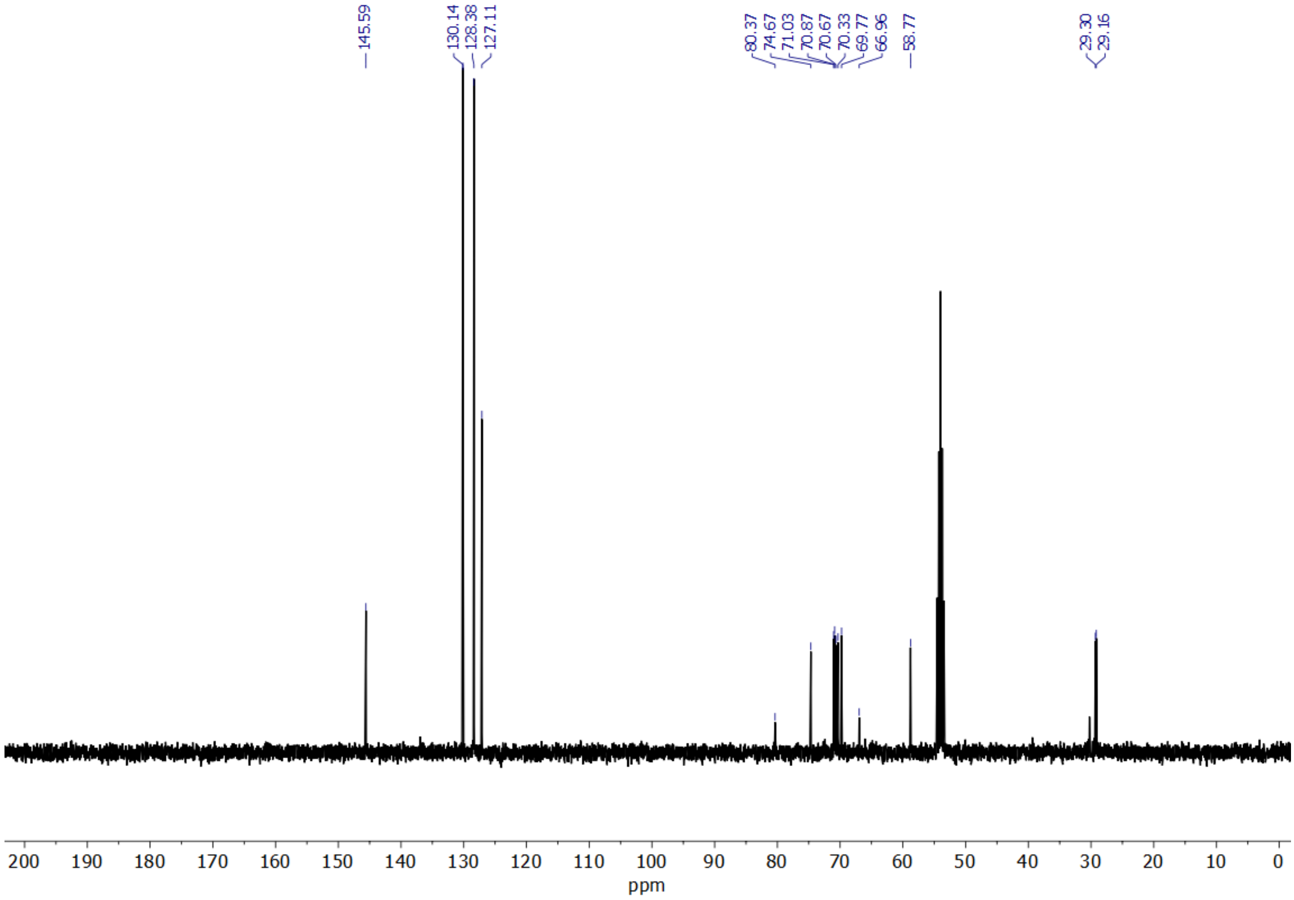

Figure S36. $^{13}C\{^{1}H\}$ NMR (101 MHz, CD_2Cl_2) spectrum of compound **14**.

Compound 16

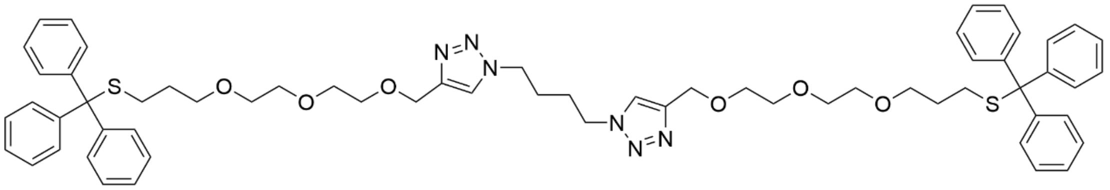

16

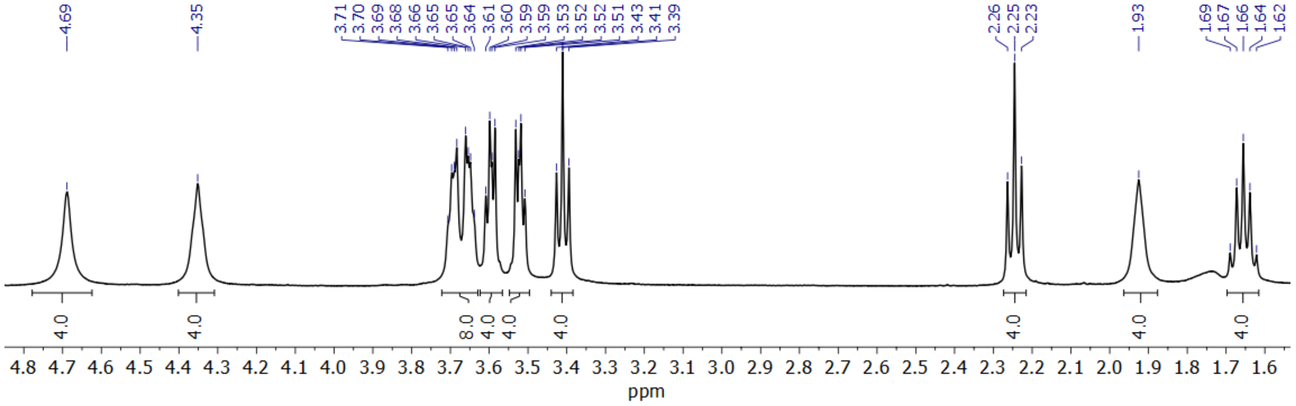

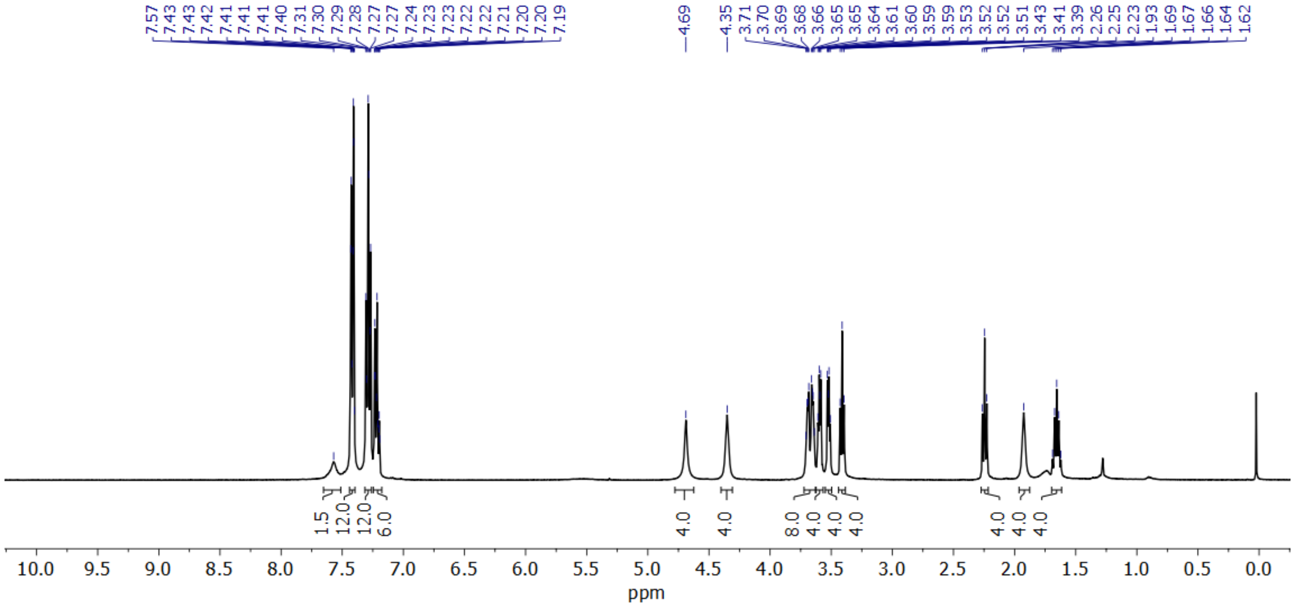

Figure S37. ^{1}H NMR (400 MHz, $CDCl_3$) spectrum of compound **16**.

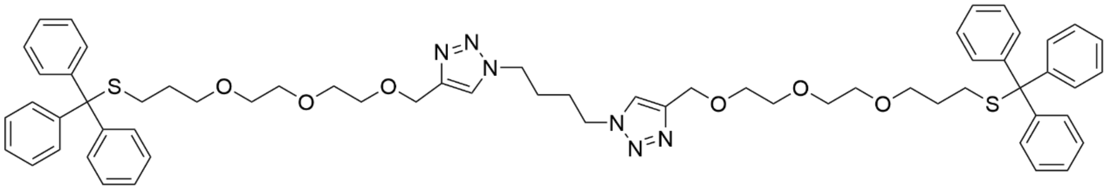

16

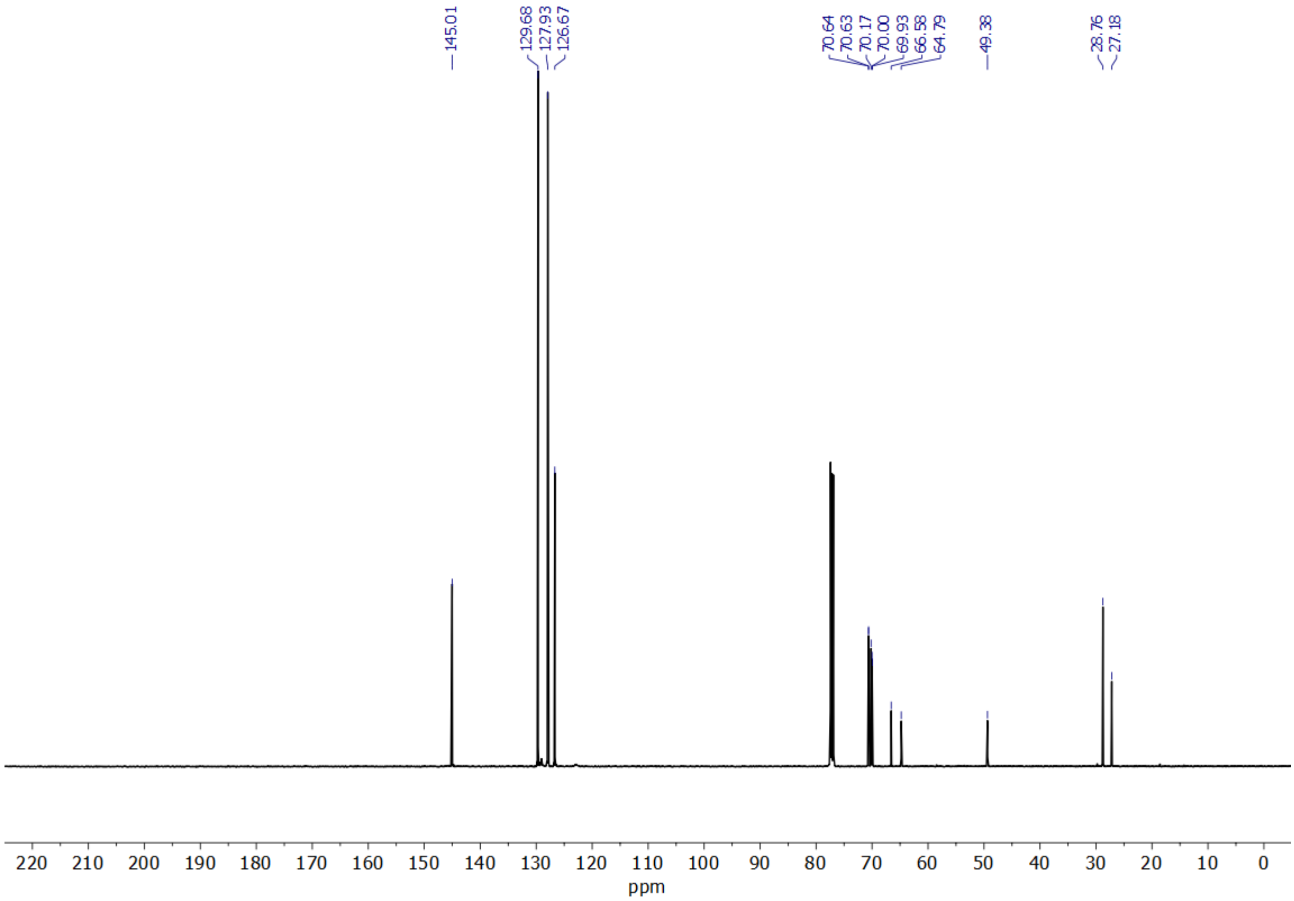

Figure S38. $^{13}C\{^1H\}$ NMR (126 MHz, $CDCl_3$) spectrum of compound **16**.

Compound 1

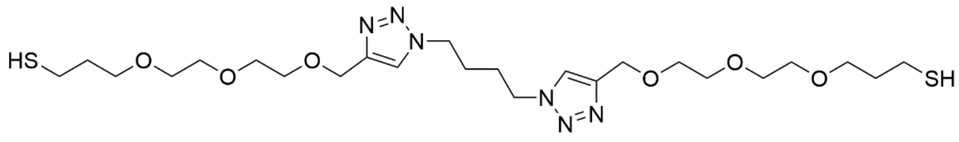

1

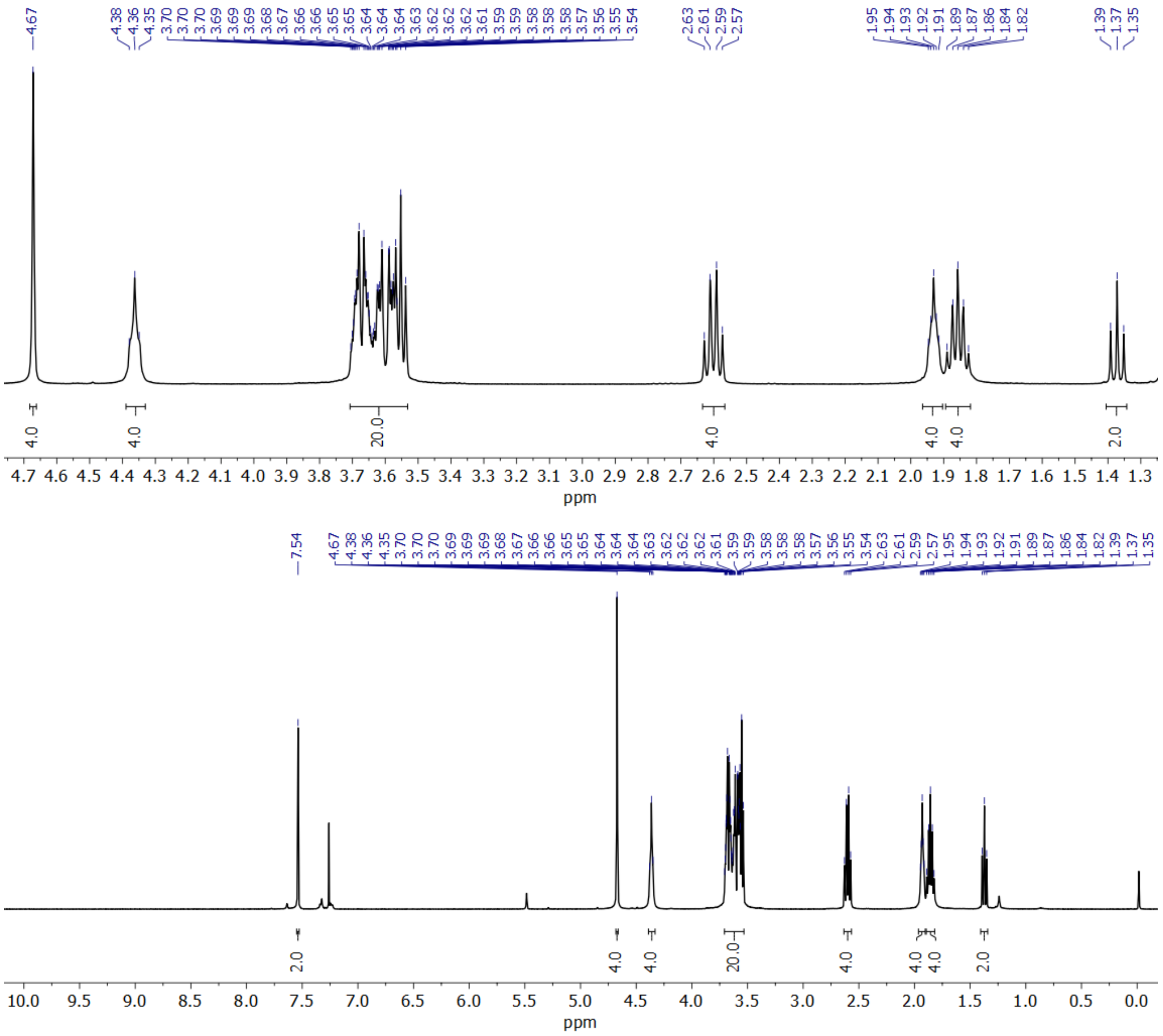

Figure S39. ^1H NMR (400 MHz, $CDCl_3$) spectrum of compound **1**.

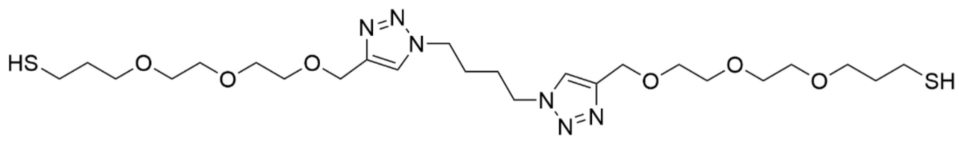

1

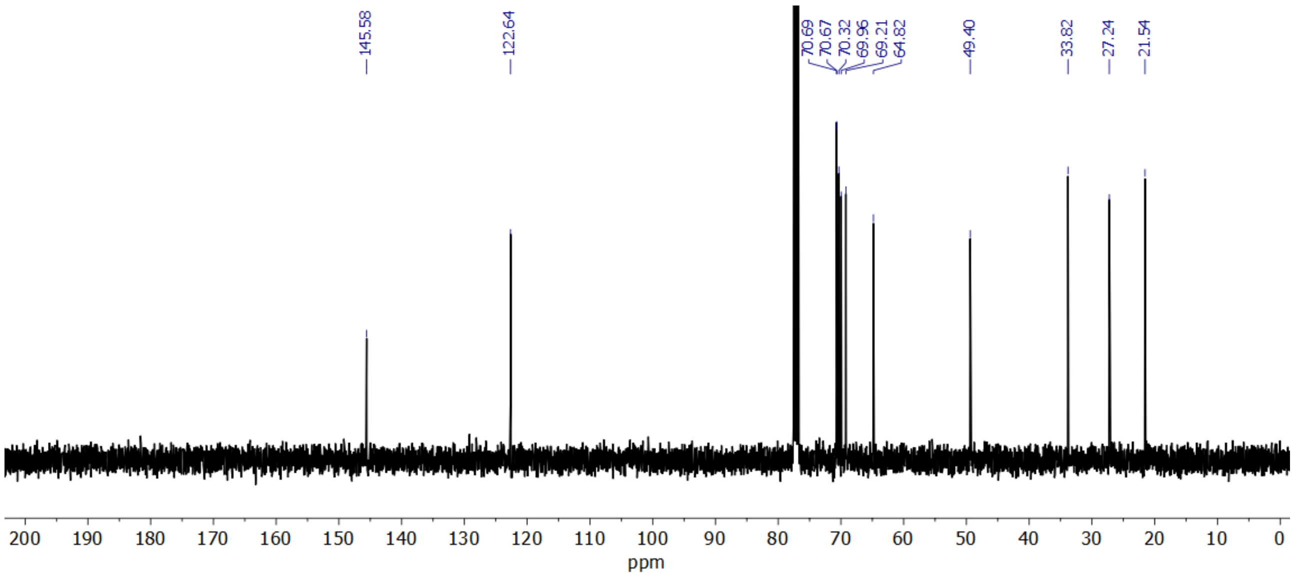

Figure S40. $^{13}C\{^{1}H\}$ NMR (101 MHz, $CDCl_3$) spectrum of compound **1**.

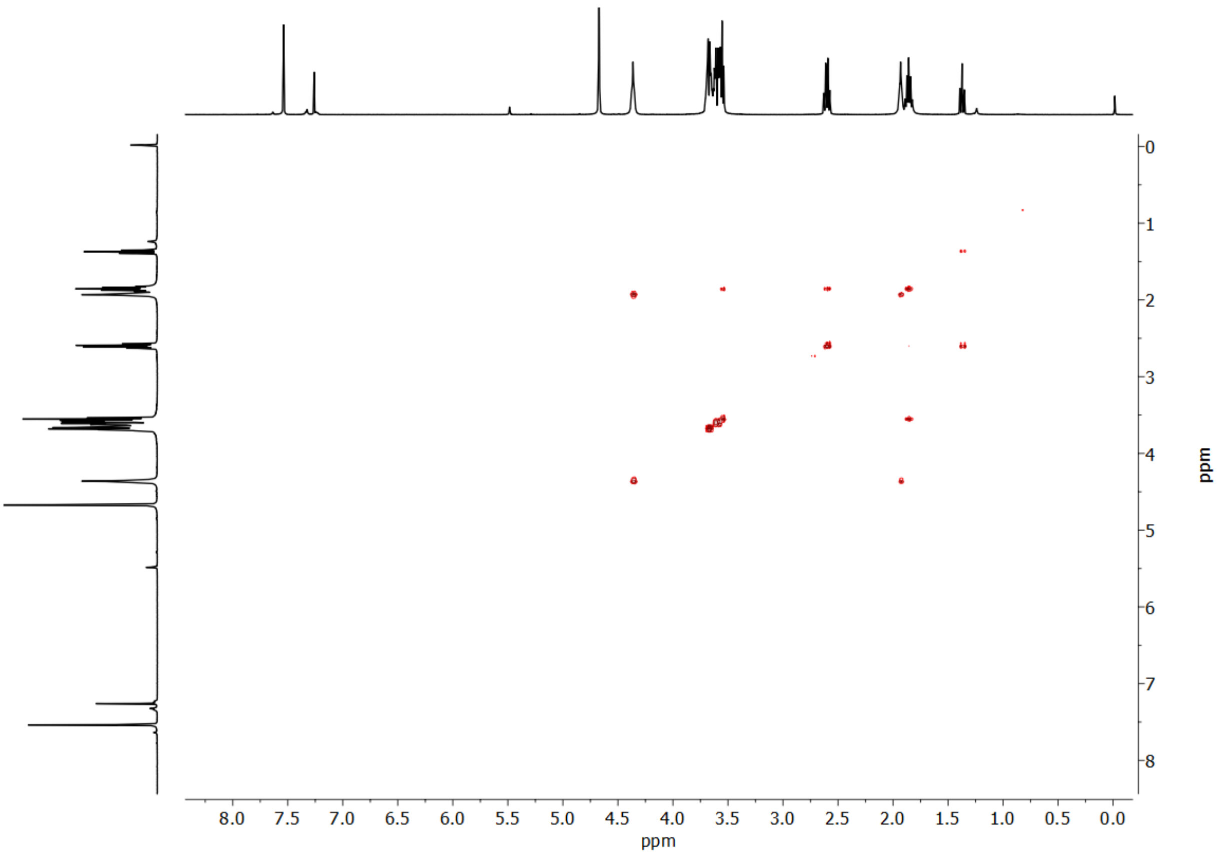

Figure S41. COSY NMR (500 MHz, $CDCl_3$) spectrum of compound **1**.

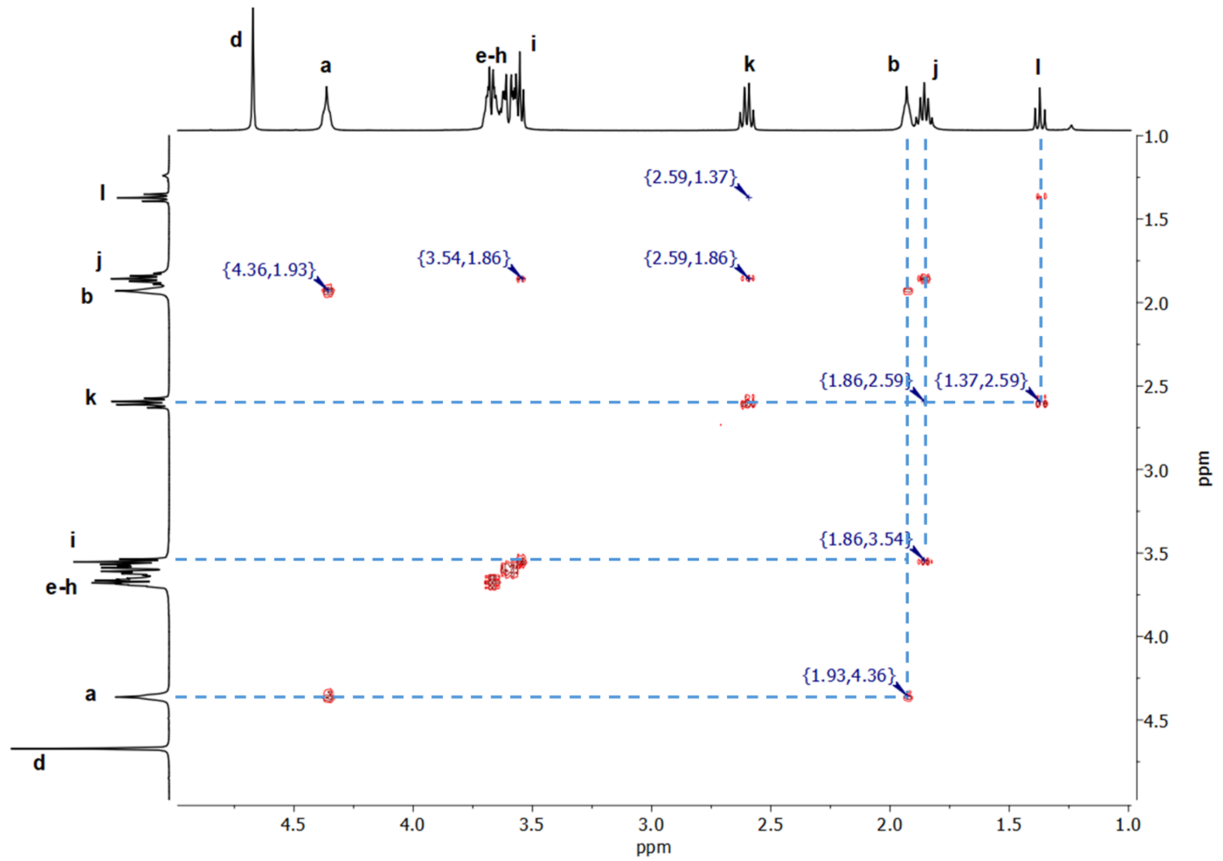

Figure S42. Partial COSY NMR (500 MHz, $CDCl_3$) spectrum of compound **1**.

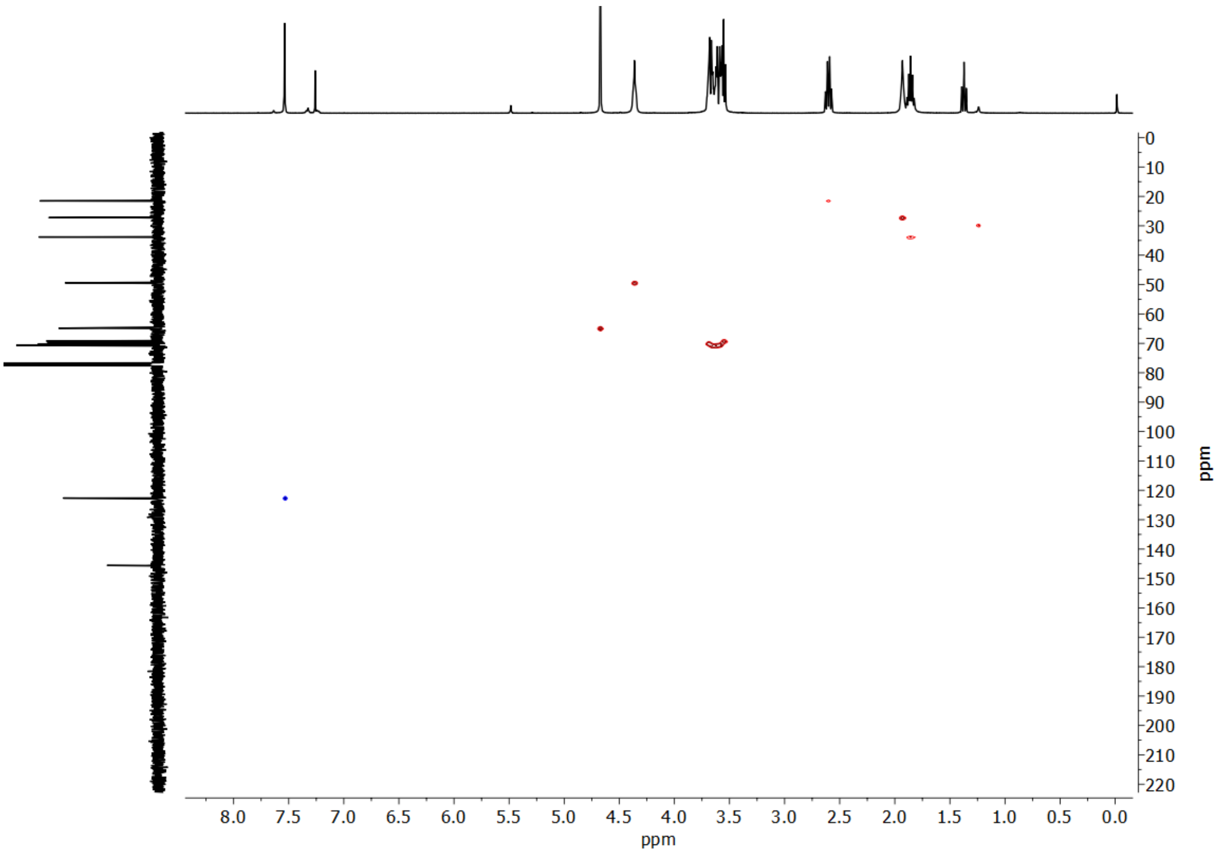

Figure S43. HSQC NMR (500 and 126 MHz, $CDCl_3$) spectrum of compound **1**.

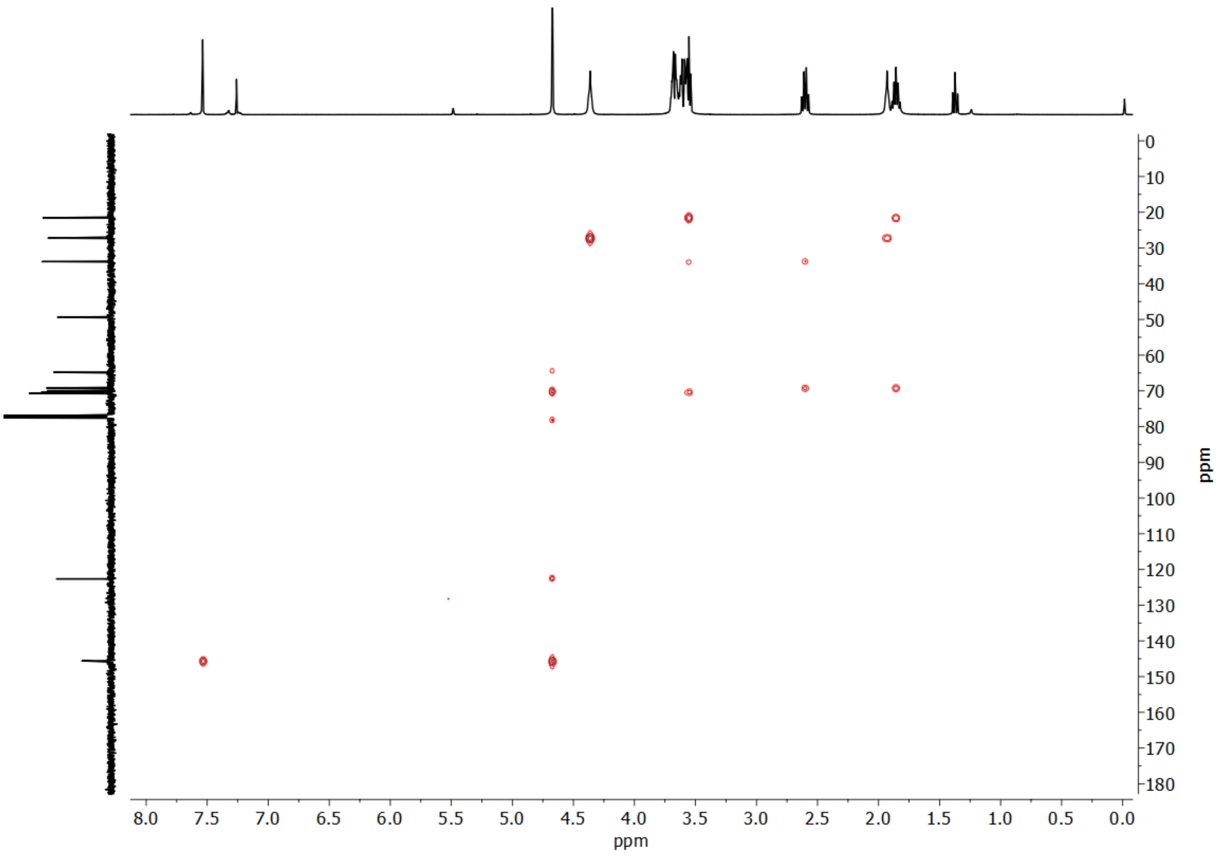

Figure S44. HMBC NMR (500 and 126 MHz, $CDCl_3$) spectrum of compound **1**.

Thread 5

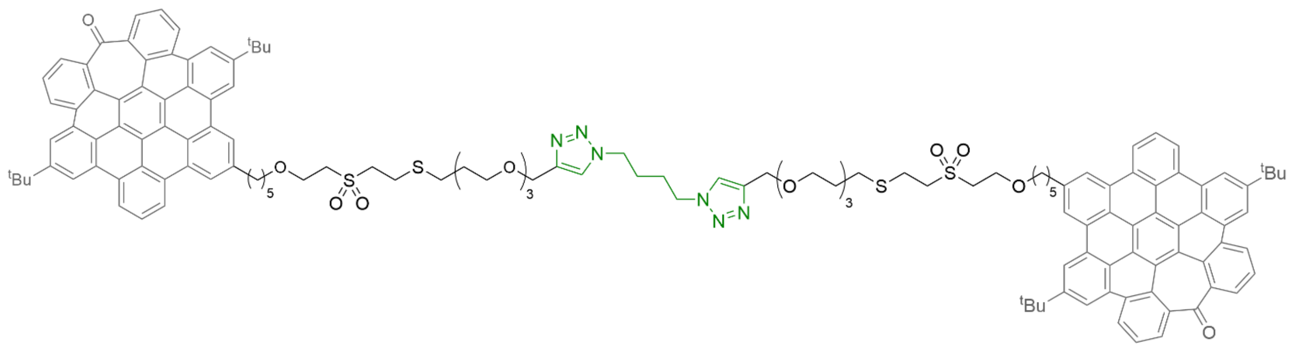

5

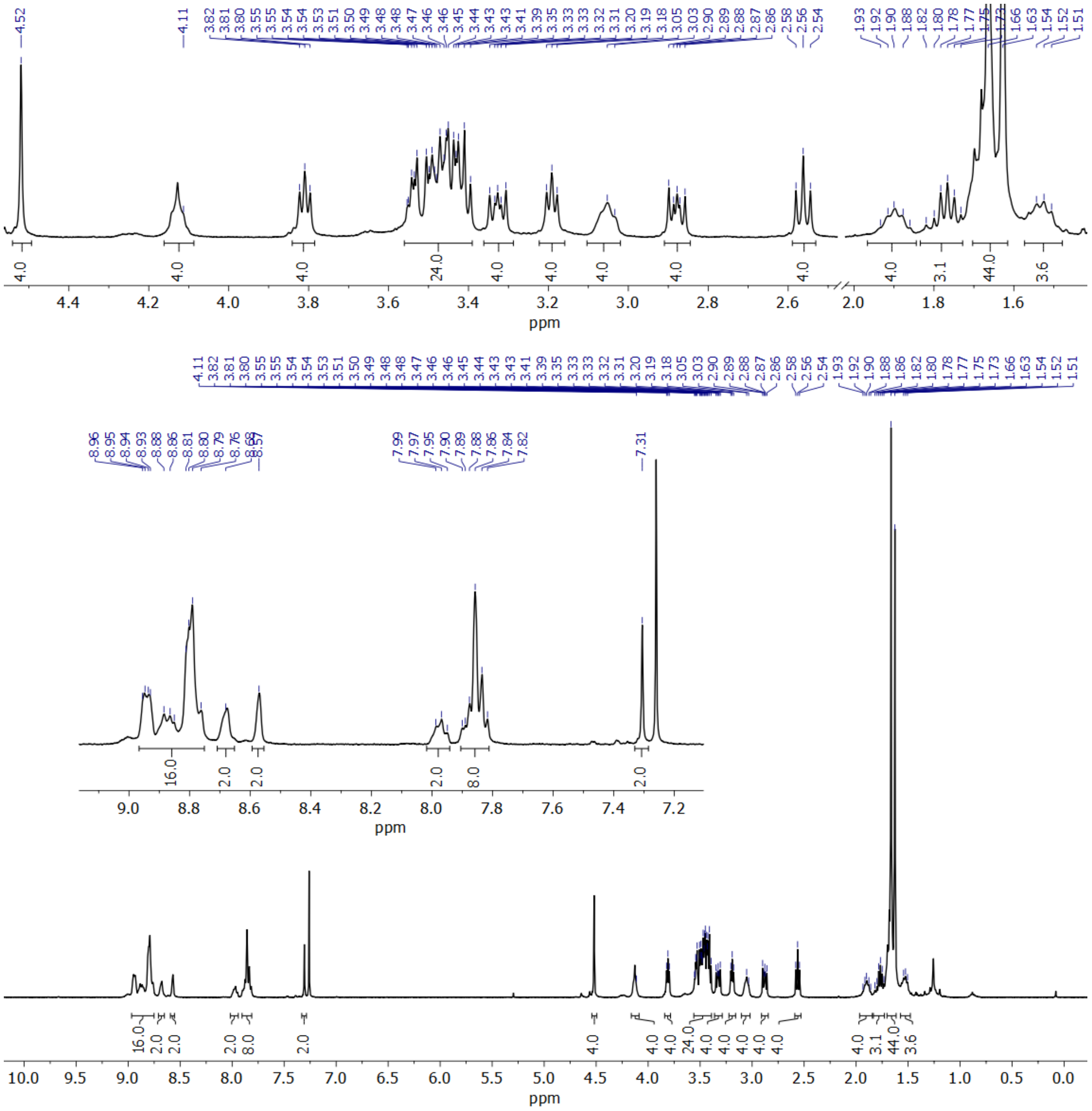

Figure S45. ^{1}H NMR (400 MHz, $CDCl_3$) spectrum of compound **5**.

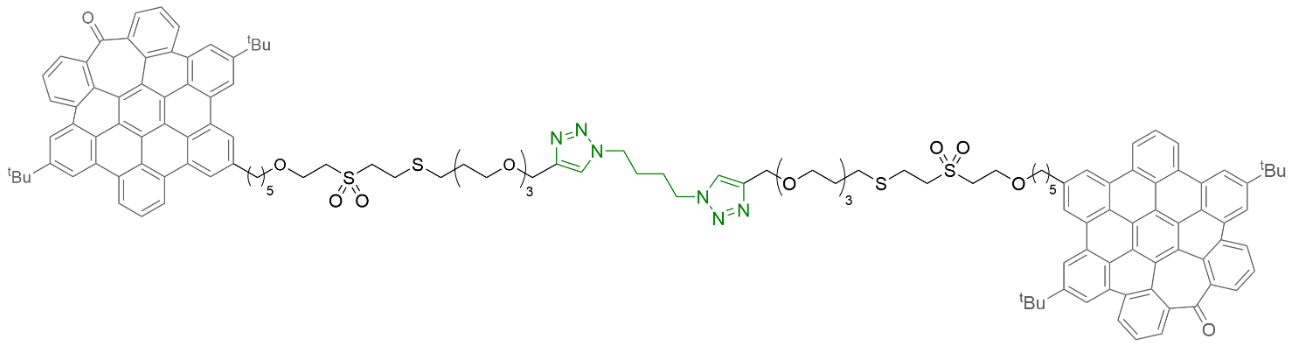

5

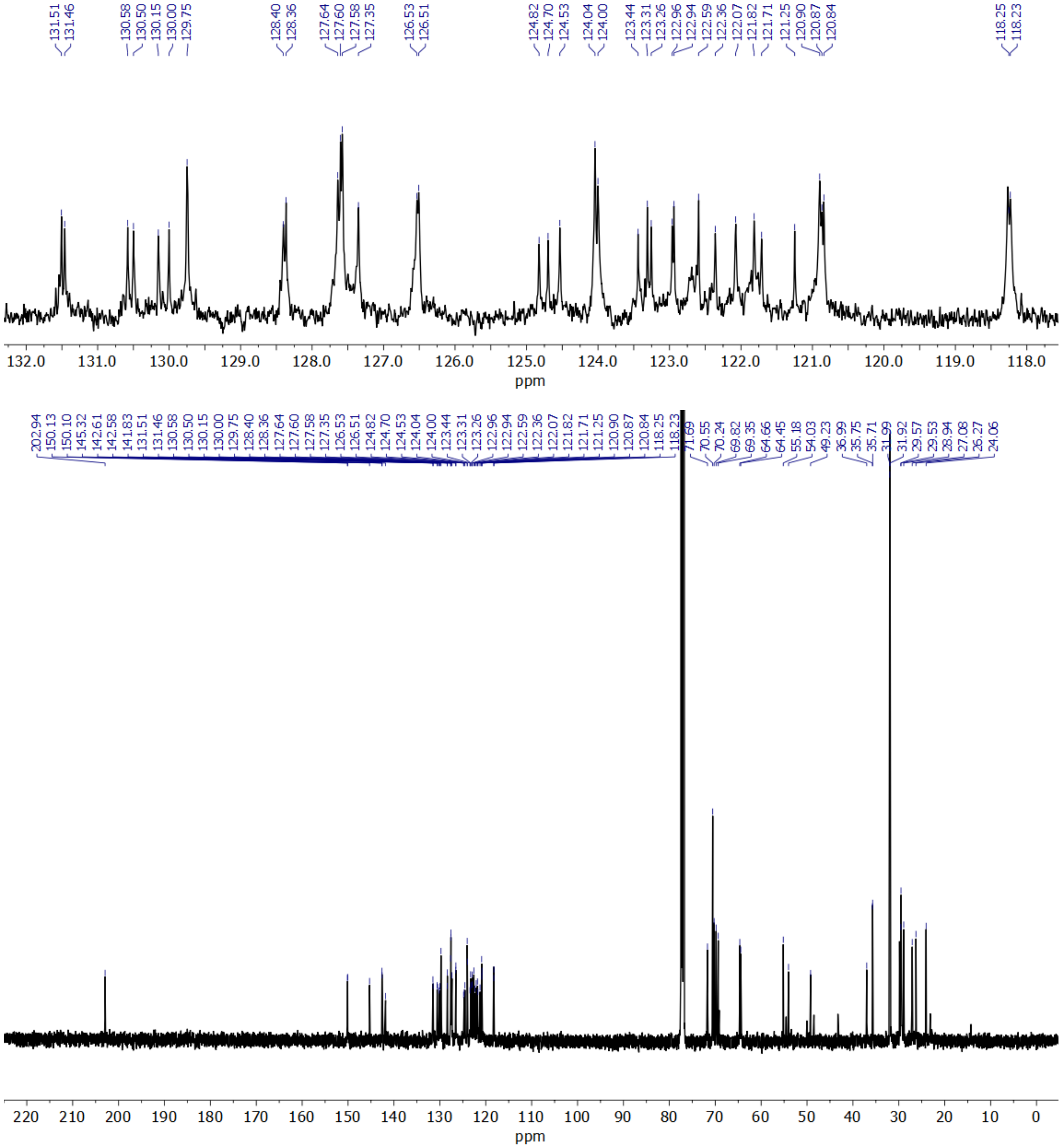

Figure S46. $^{13}C\{^{1}H\}$ NMR (126 MHz, $CDCl_3$) spectrum of compound **5**.

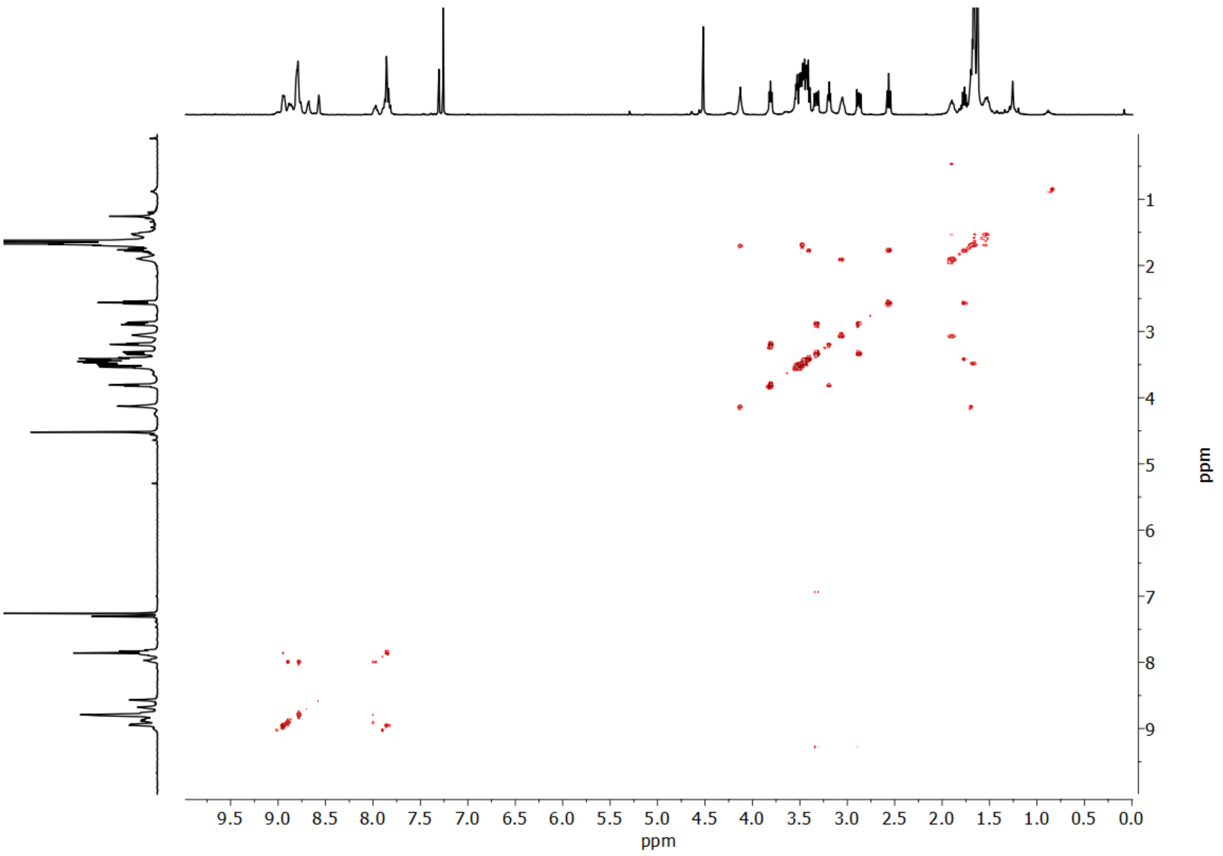

Figure S47. COSY NMR (500 MHz, $CDCl_3$) spectrum of compound **5**.

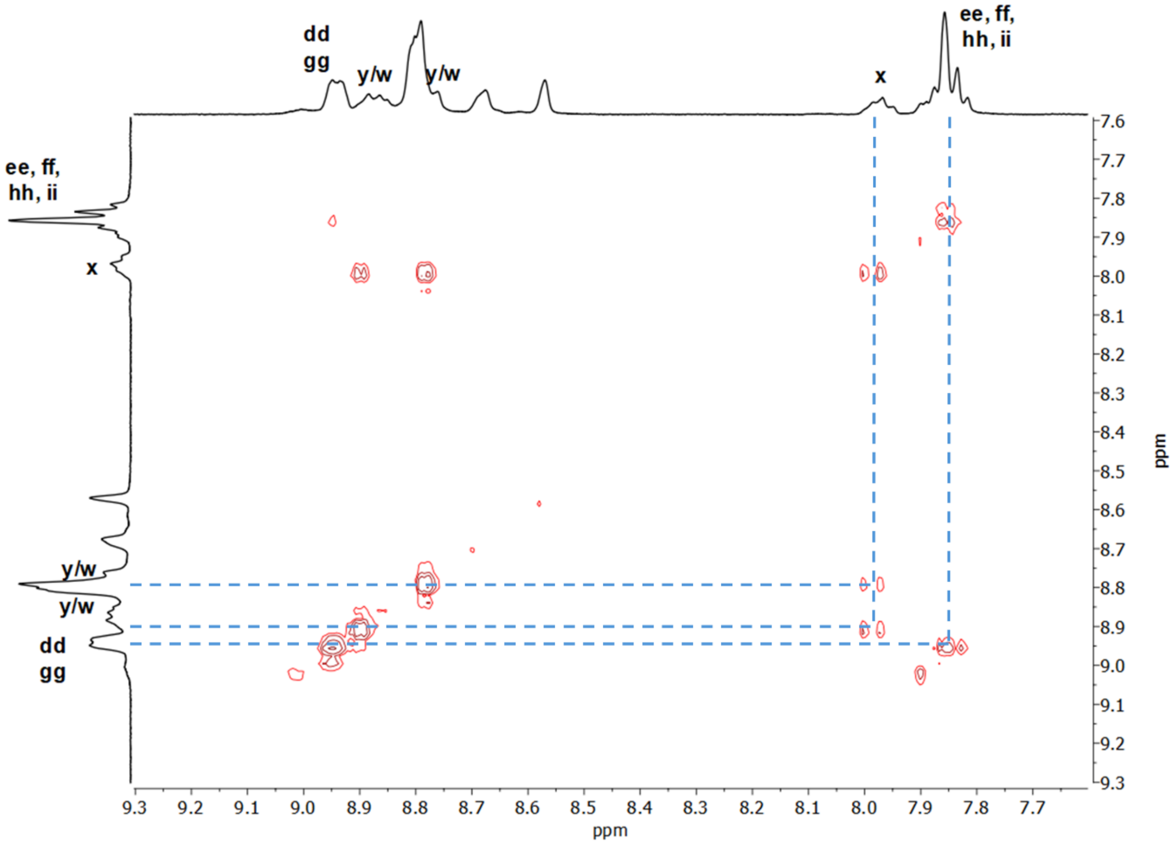

Figure S48. Partial COSY NMR (500 MHz, $CDCl_3$) spectrum of compound **5**.

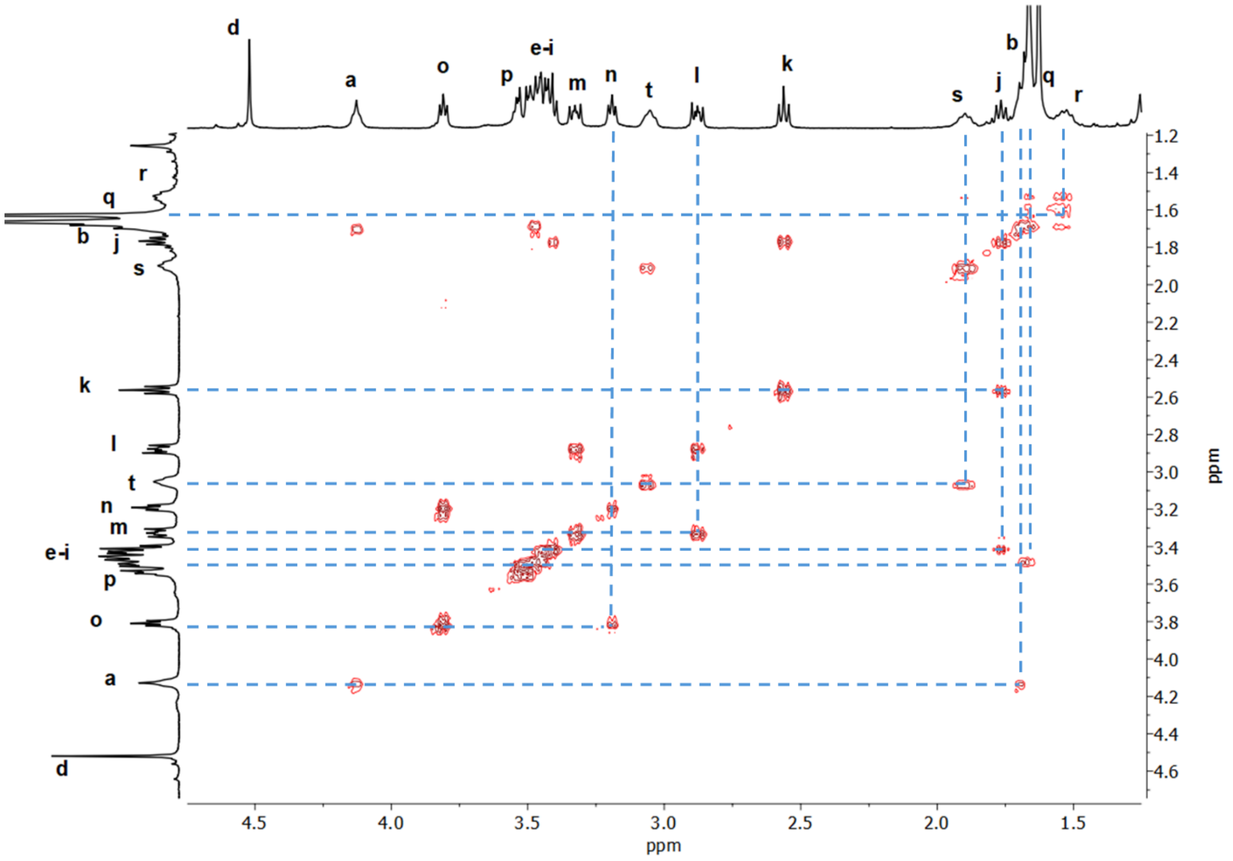

Figure S49. Partial COSY NMR (500 MHz, $CDCl_3$) spectrum of compound **5**.

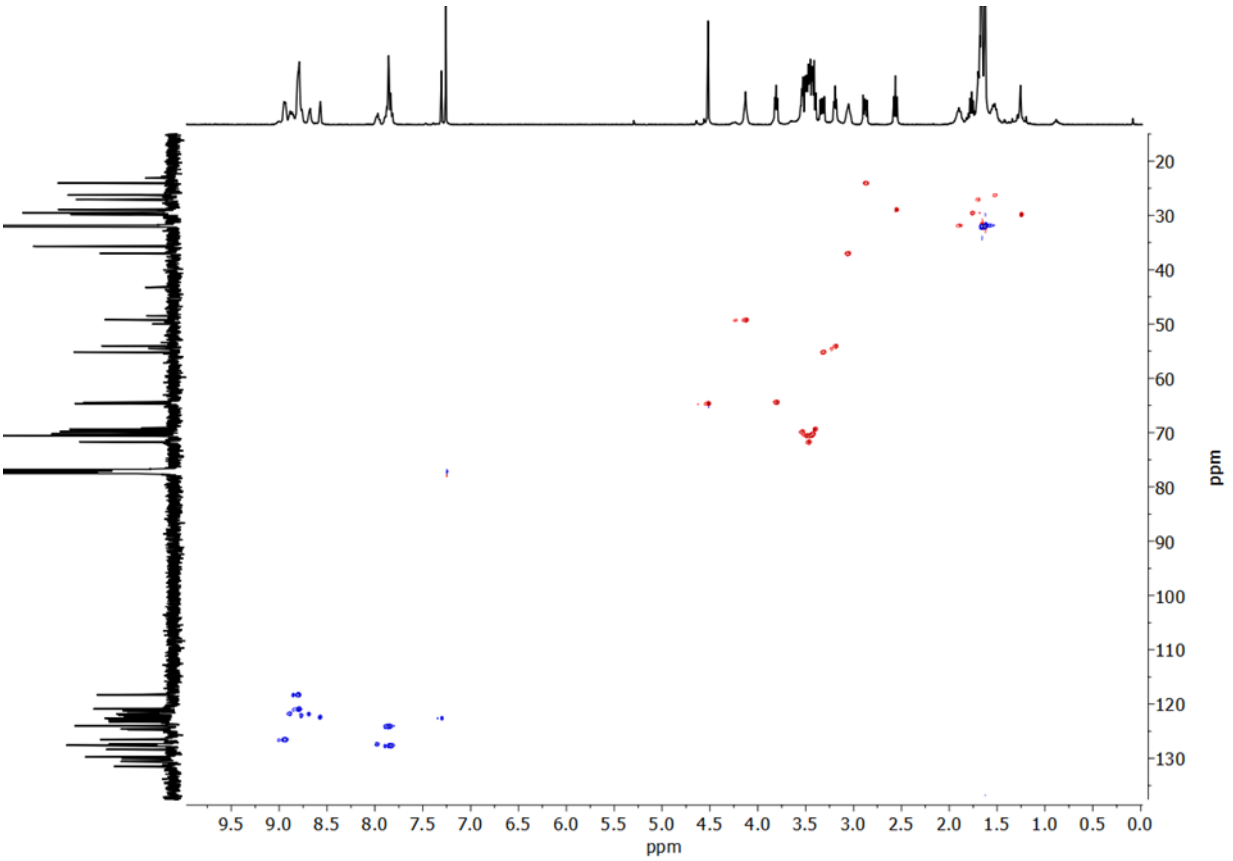

Figure S50. HSQC NMR (500 and 126 MHz, $CDCl_3$) spectrum of compound **5**.

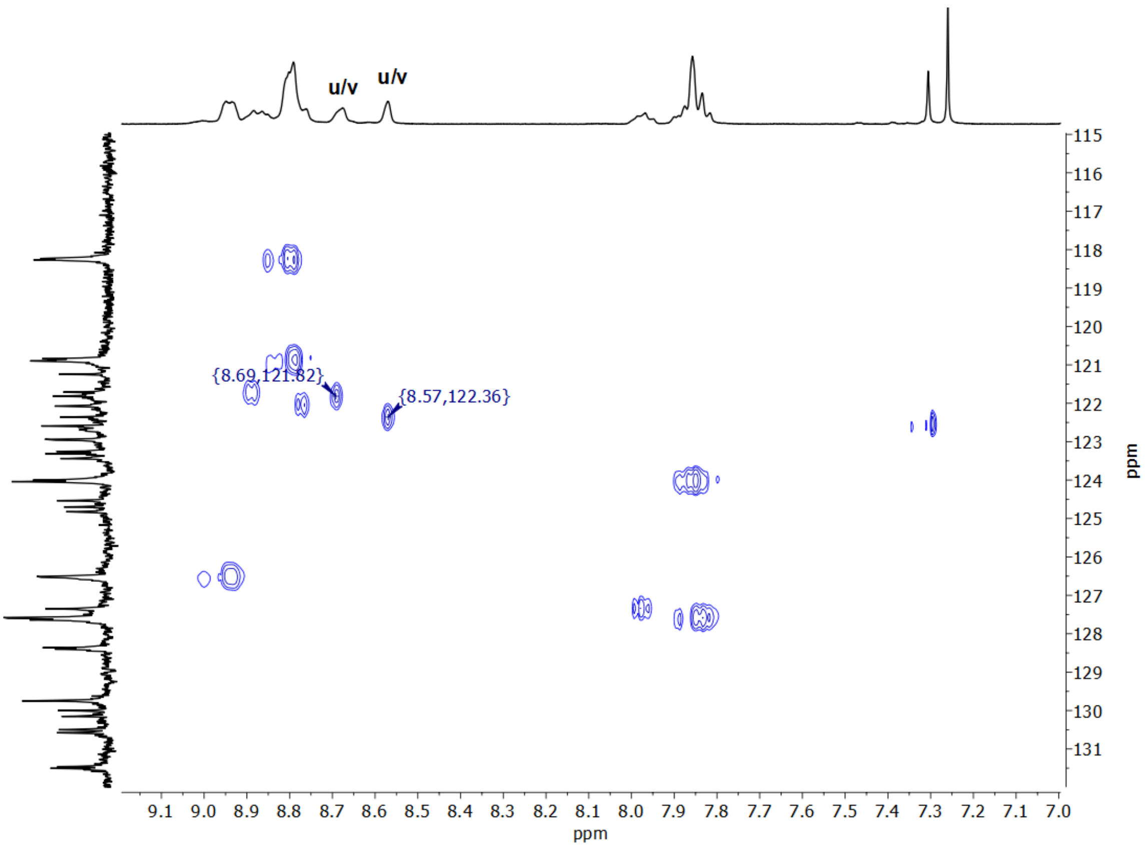

Figure S51. Partial HSQC NMR (500 and 126 MHz, $CDCl_3$) spectrum of compound **5**.

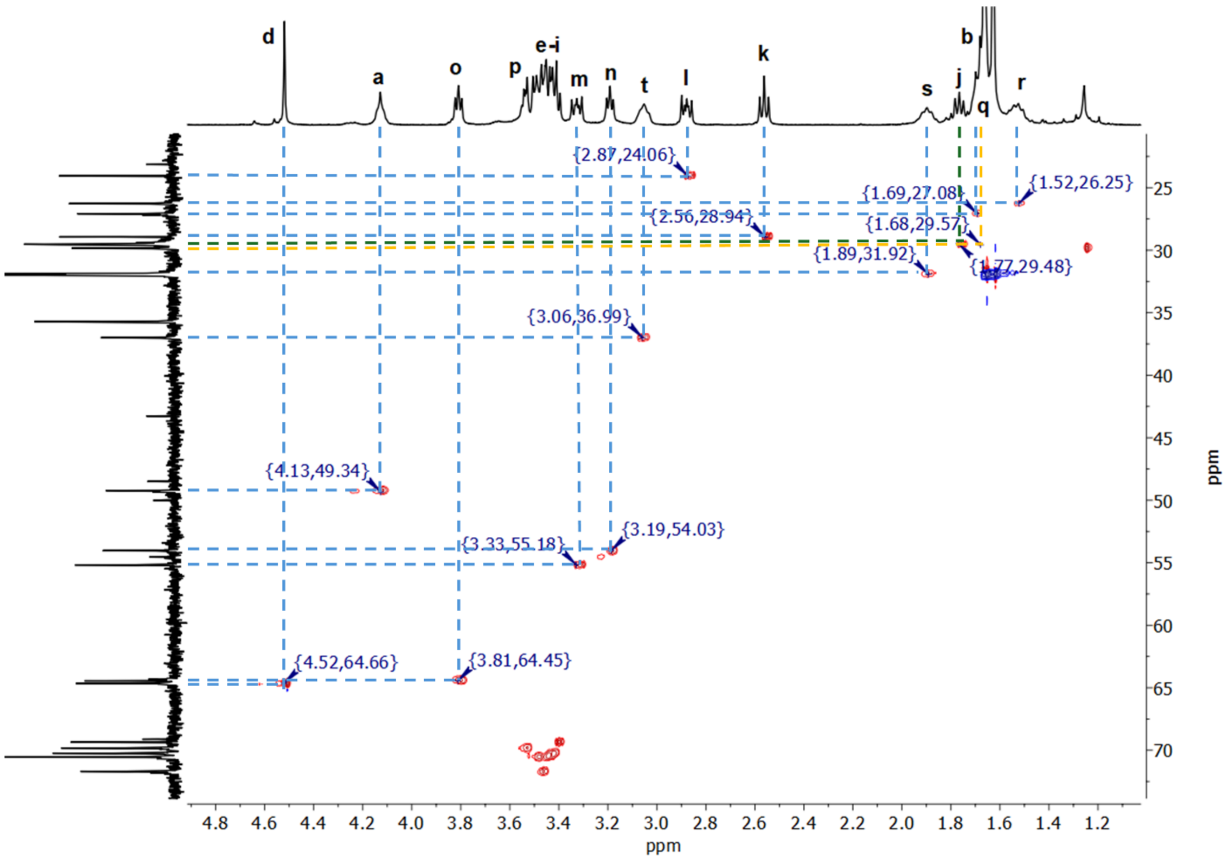

Figure S52. Partial HSQC NMR (500 and 126 MHz, $CDCl_3$) spectrum of compound **5**.

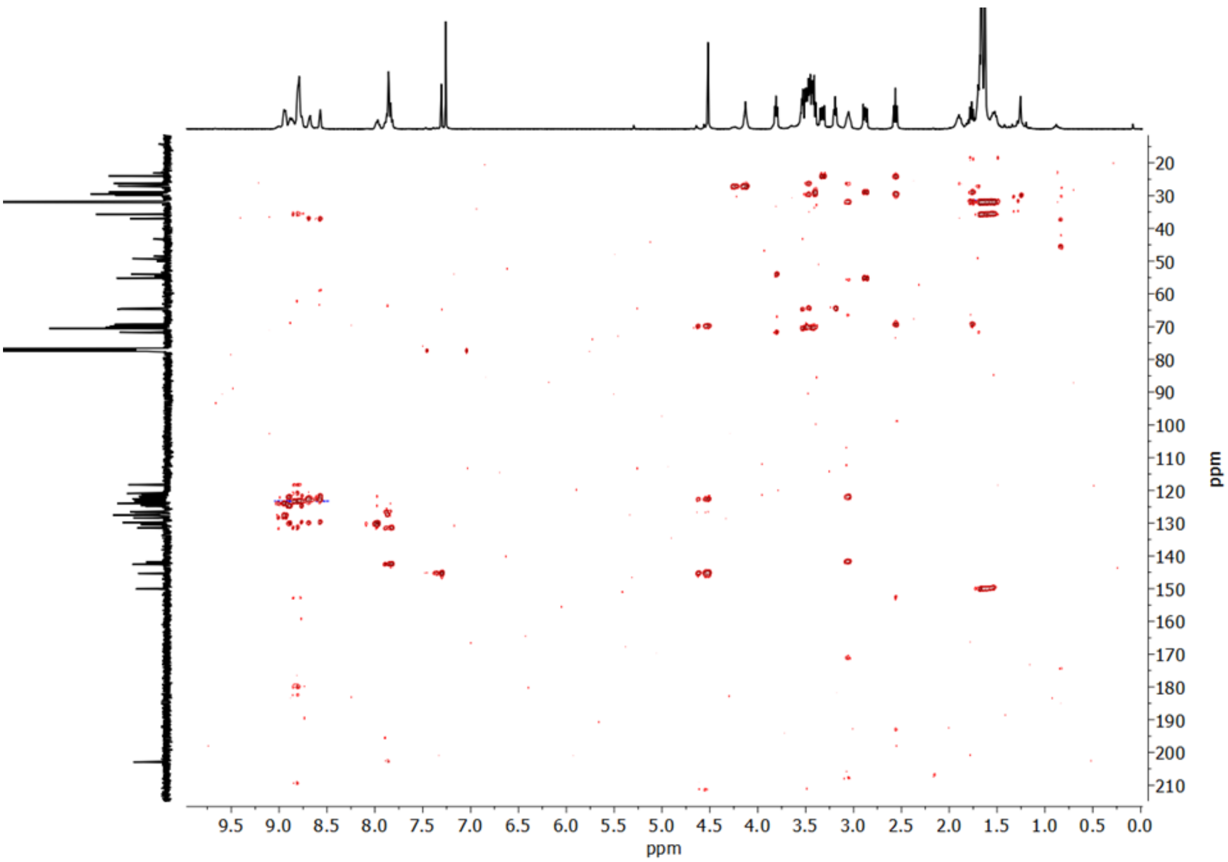

Figure S53. HMBC NMR (500 and 126 MHz, $CDCl_3$) spectrum of compound **5**.

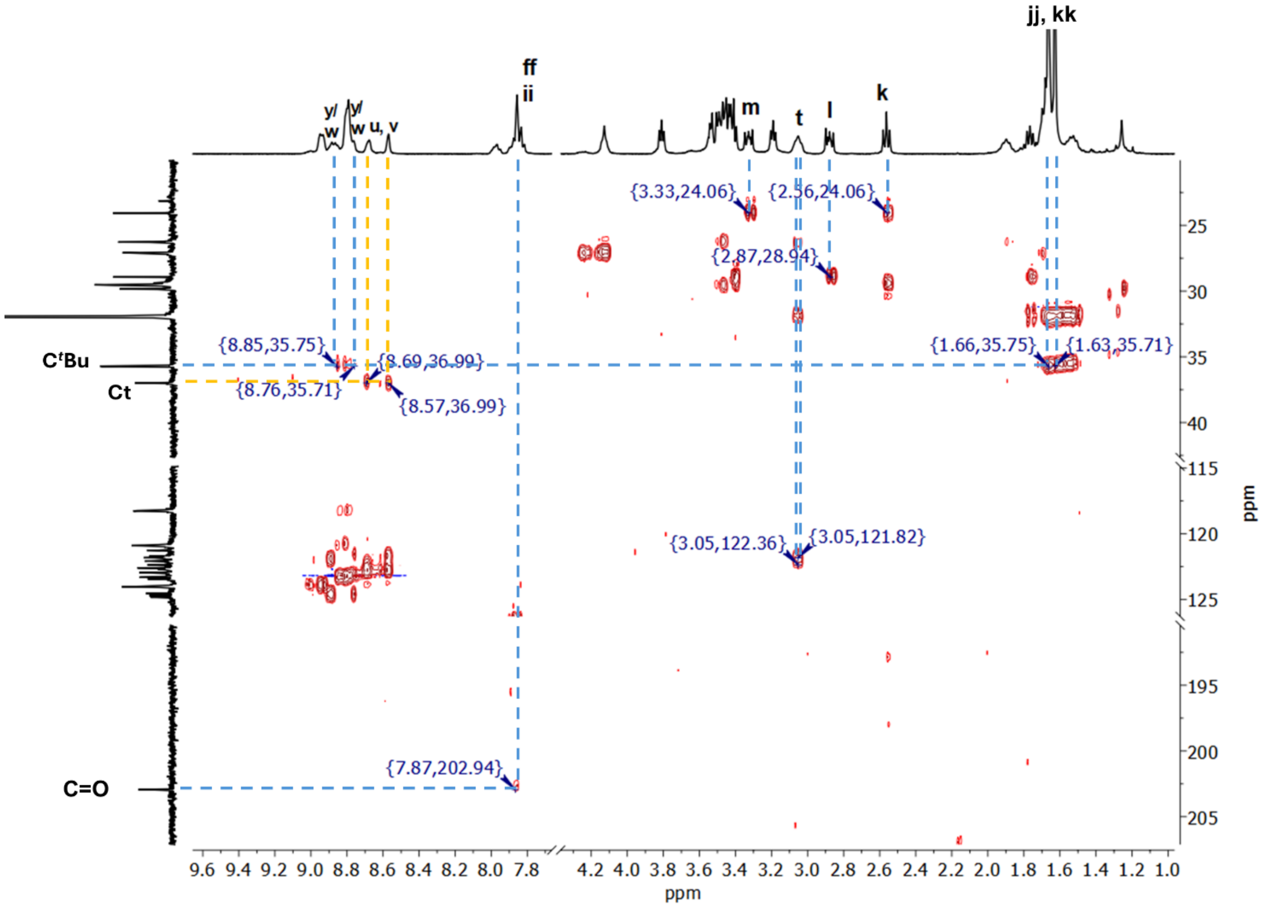

Figure S54. Partial HMBC NMR (500 and 126 MHz, $CDCl_3$) spectrum of compound **5**.

Rotaxane 4

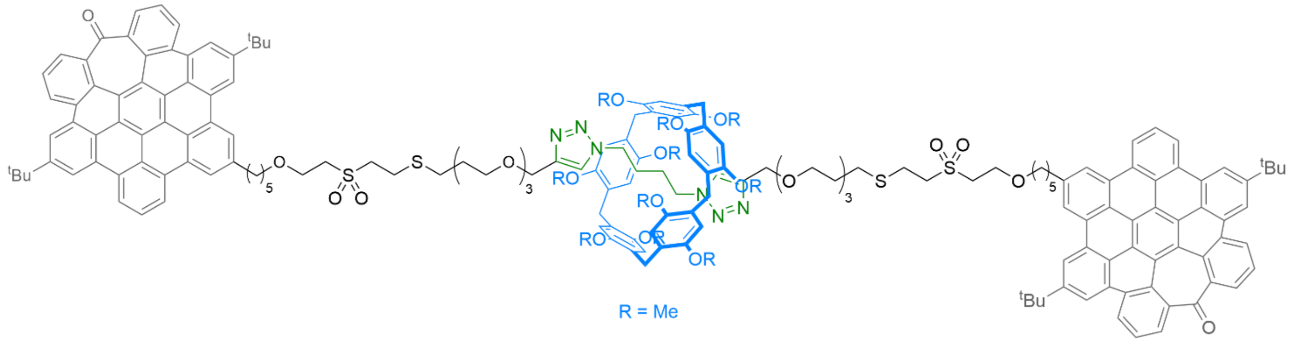

4

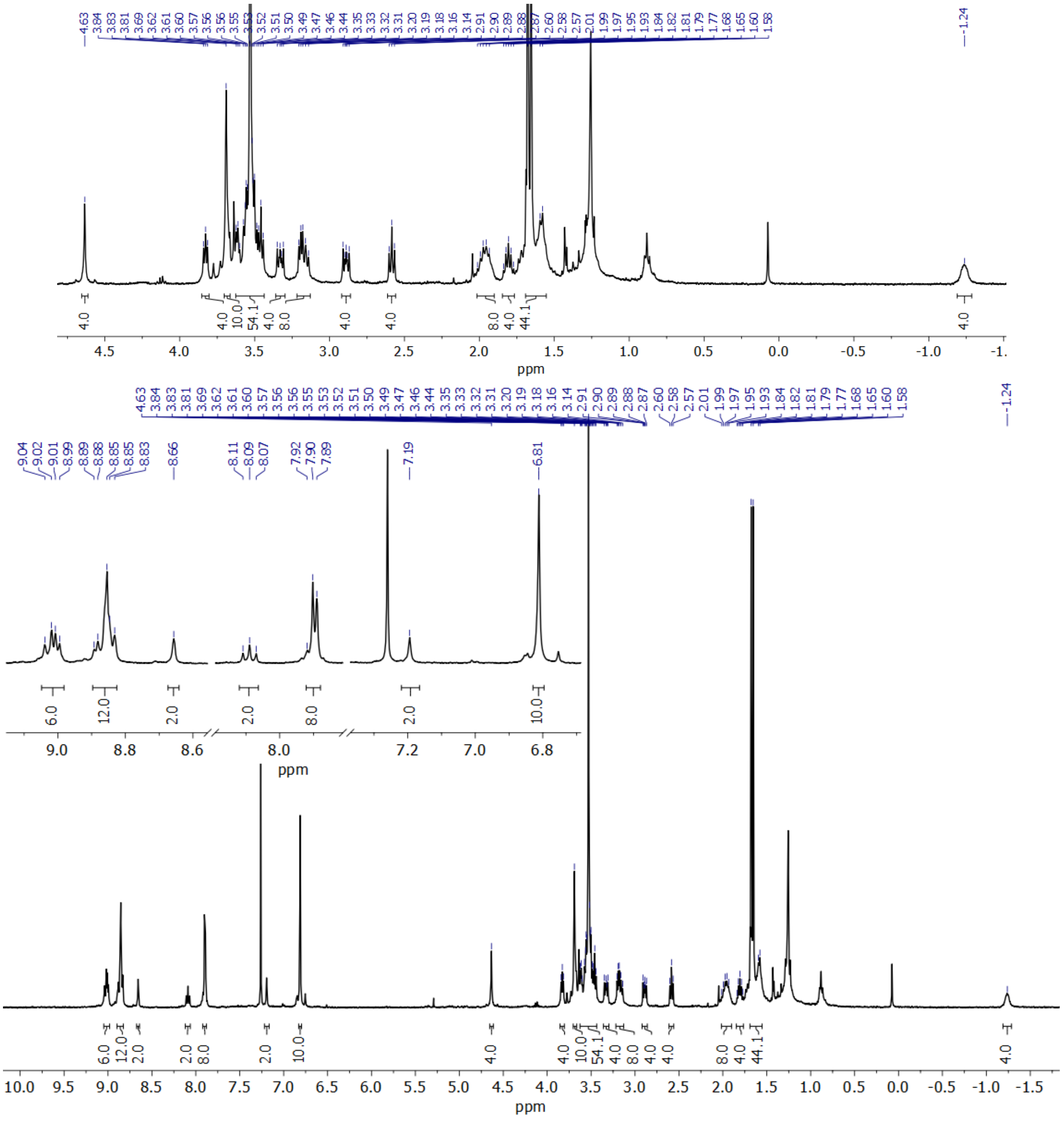

Figure S55. ^{1}H NMR (400 MHz, $CDCl_3$) spectrum of compound **4**.

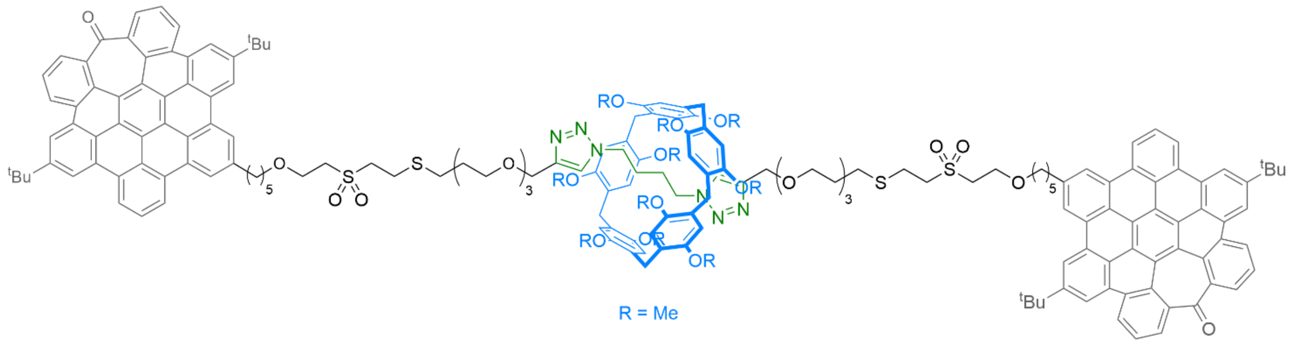

4

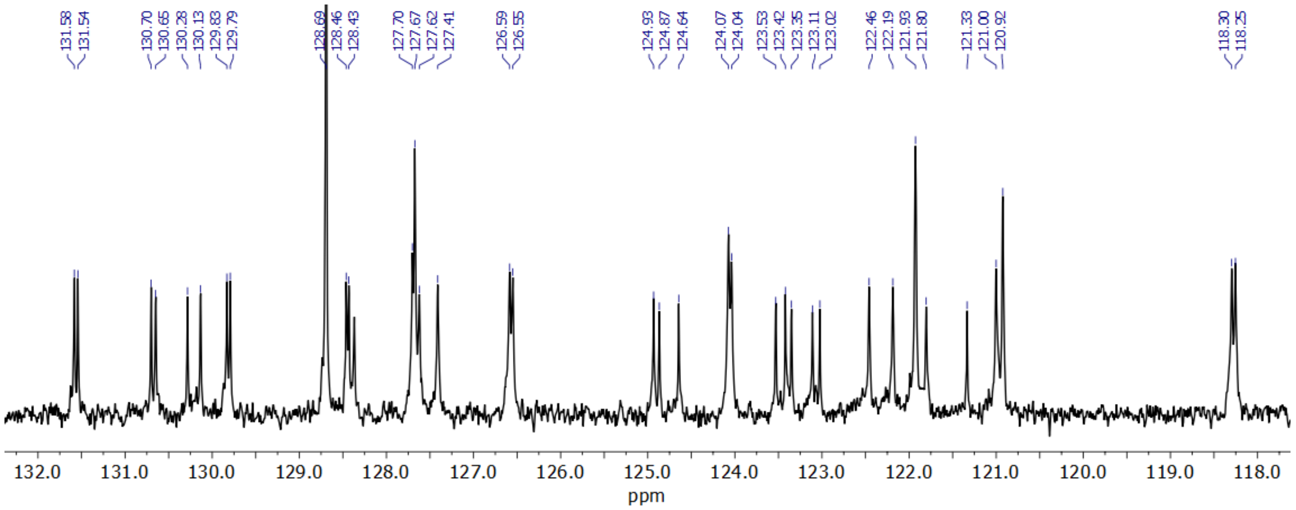

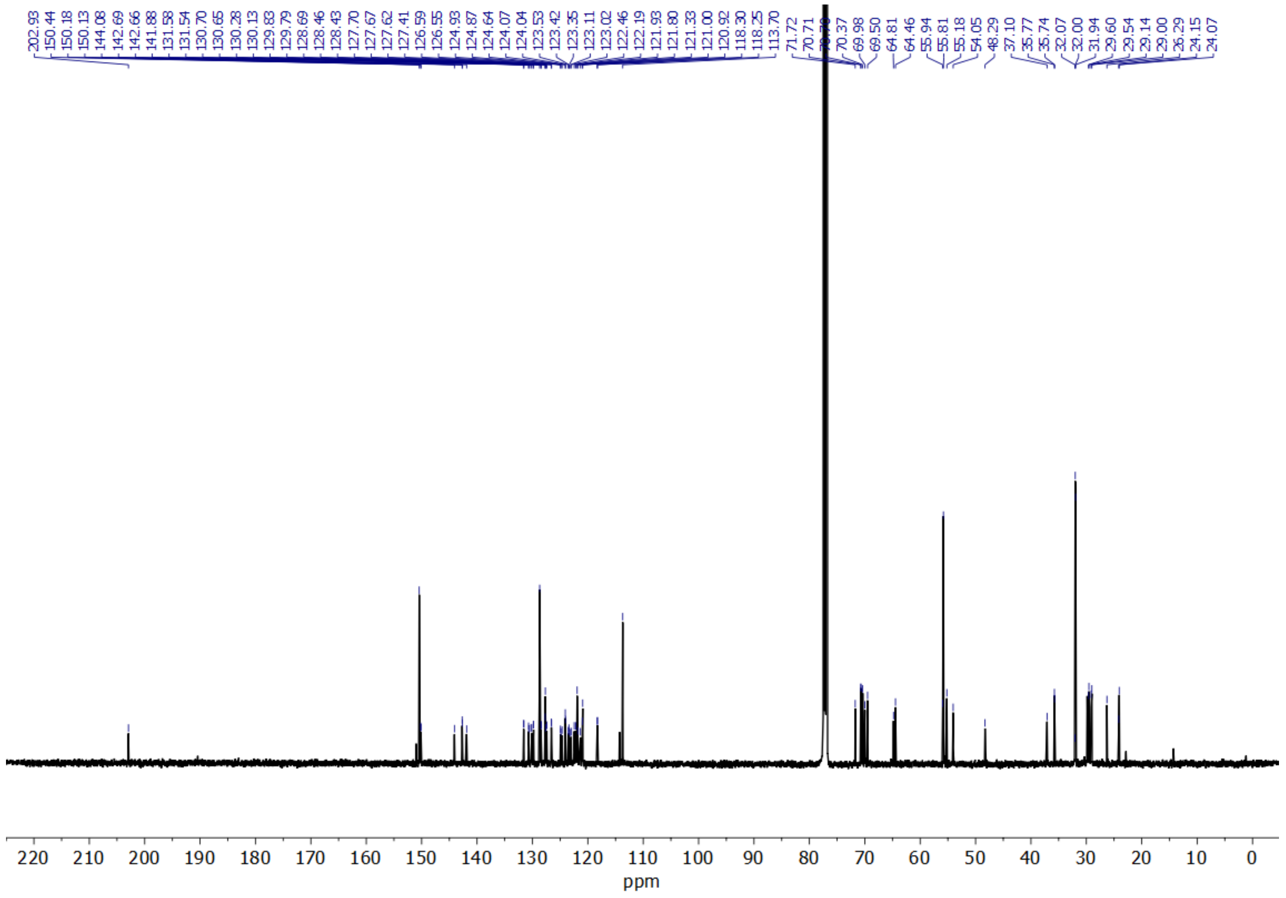

Figure S56. $^{13}C\{^{1}H\}$ NMR (126 MHz, $CDCl_3$) spectrum of compound **4**.

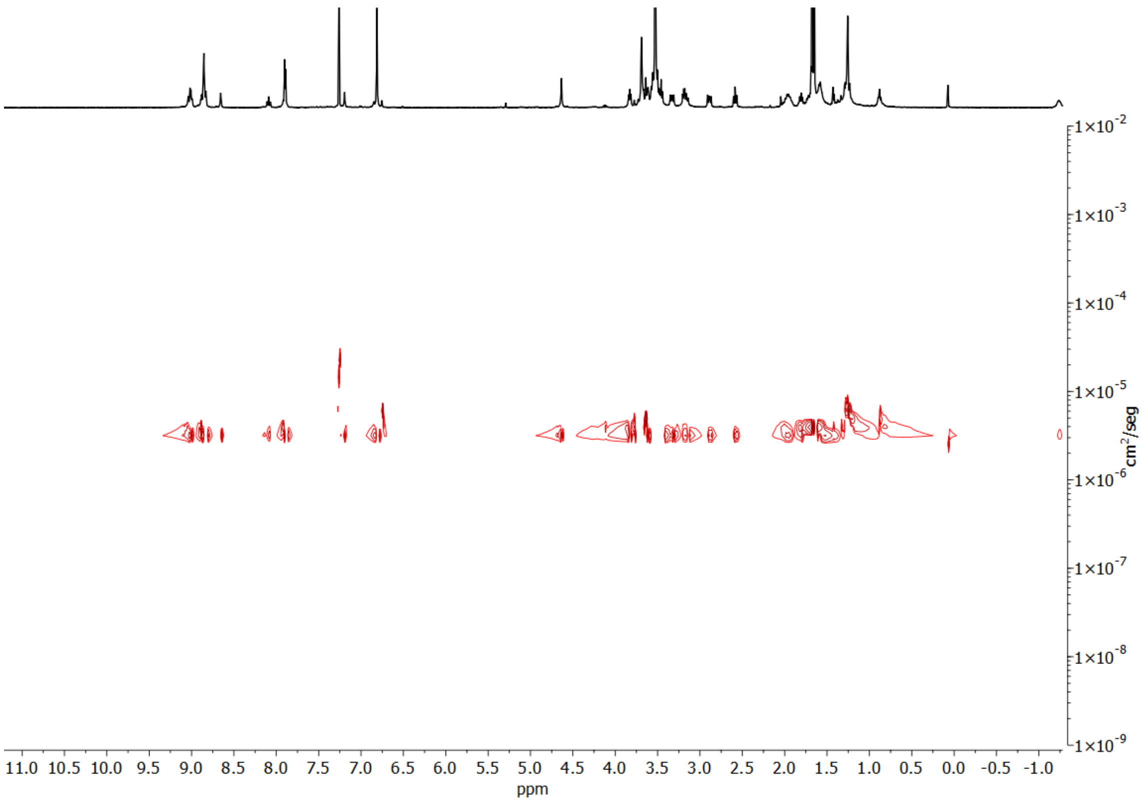

Figure S57. DOSY NMR (500 MHz, $CDCl_3$) spectrum of compound **4**. $CDCl_3$ was used as reference (D = 1.9×10^{-9} m^2/s,).

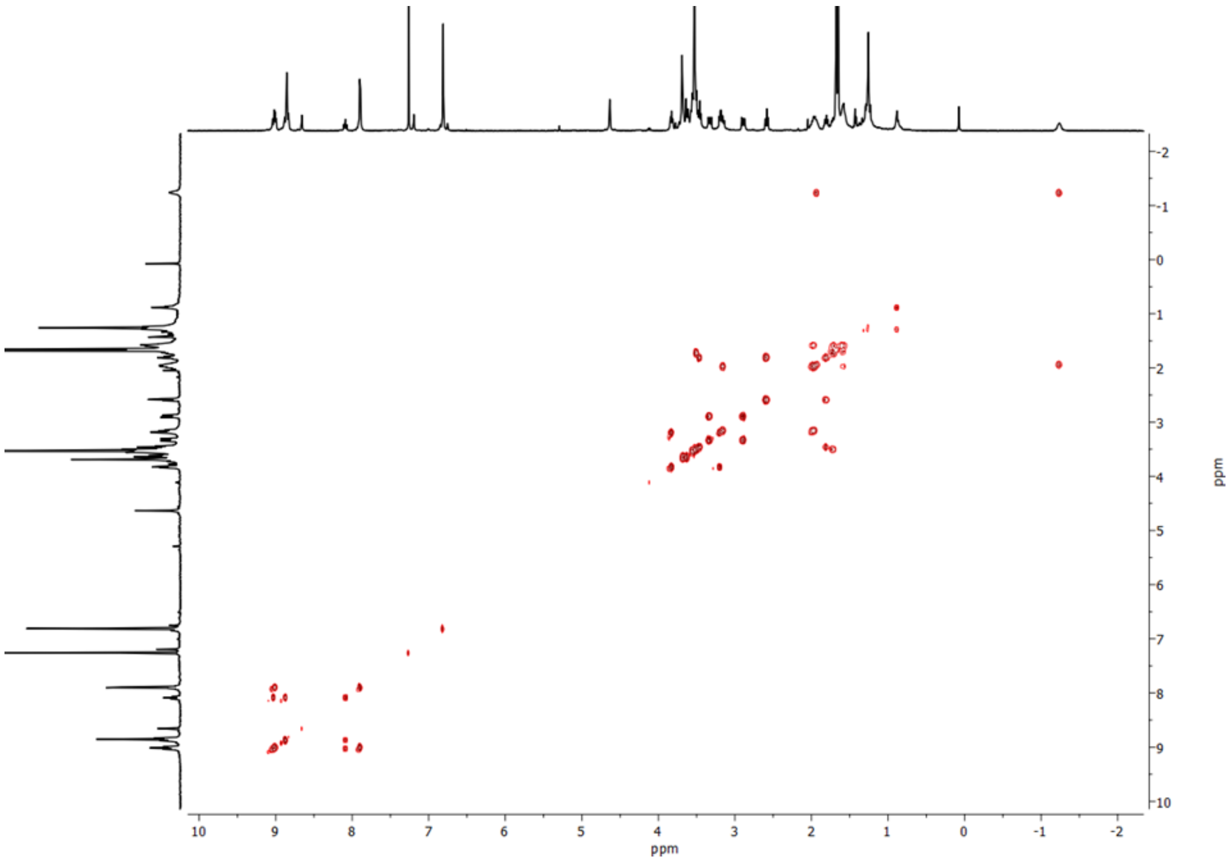

Figure S58. COSY NMR (500 MHz, $CDCl_3$) spectrum of compound **4**.

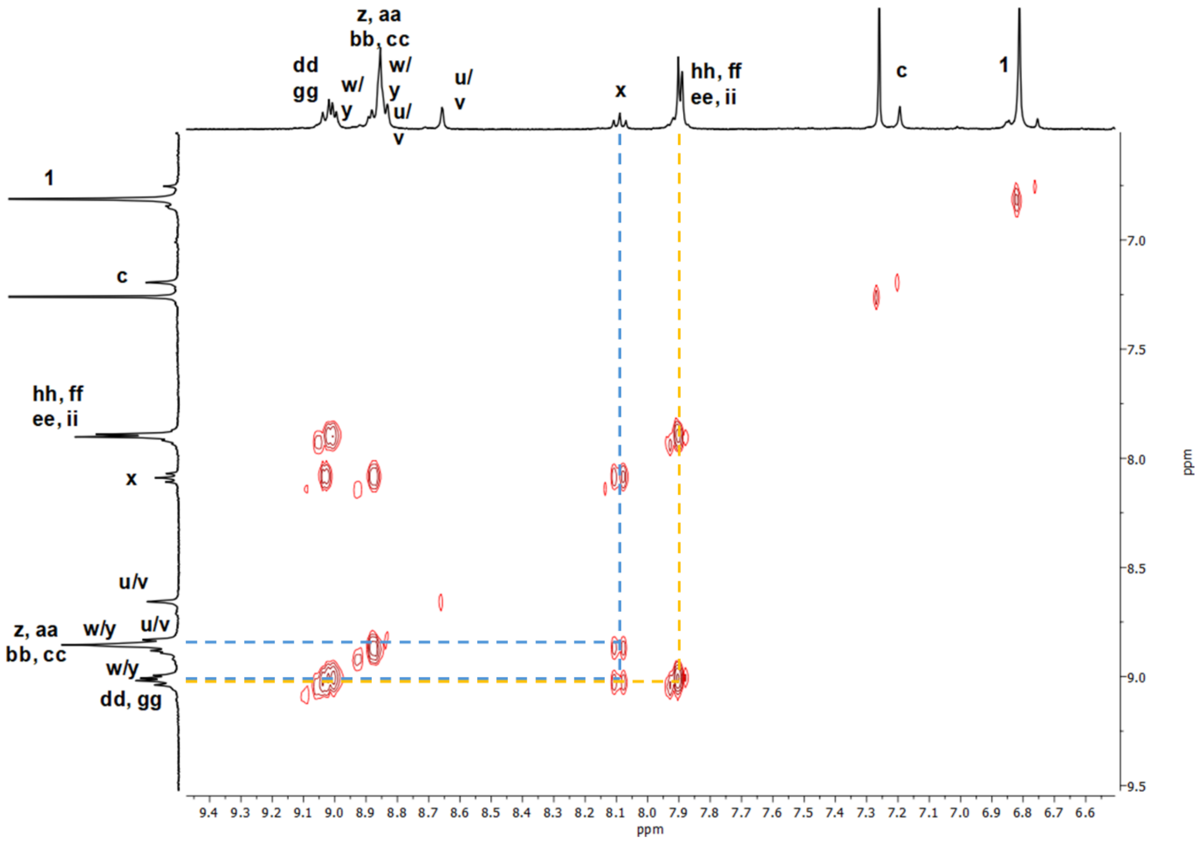

Figure S59. Partial COSY NMR (500 MHz, $CDCl_3$) spectrum of compound **4**.

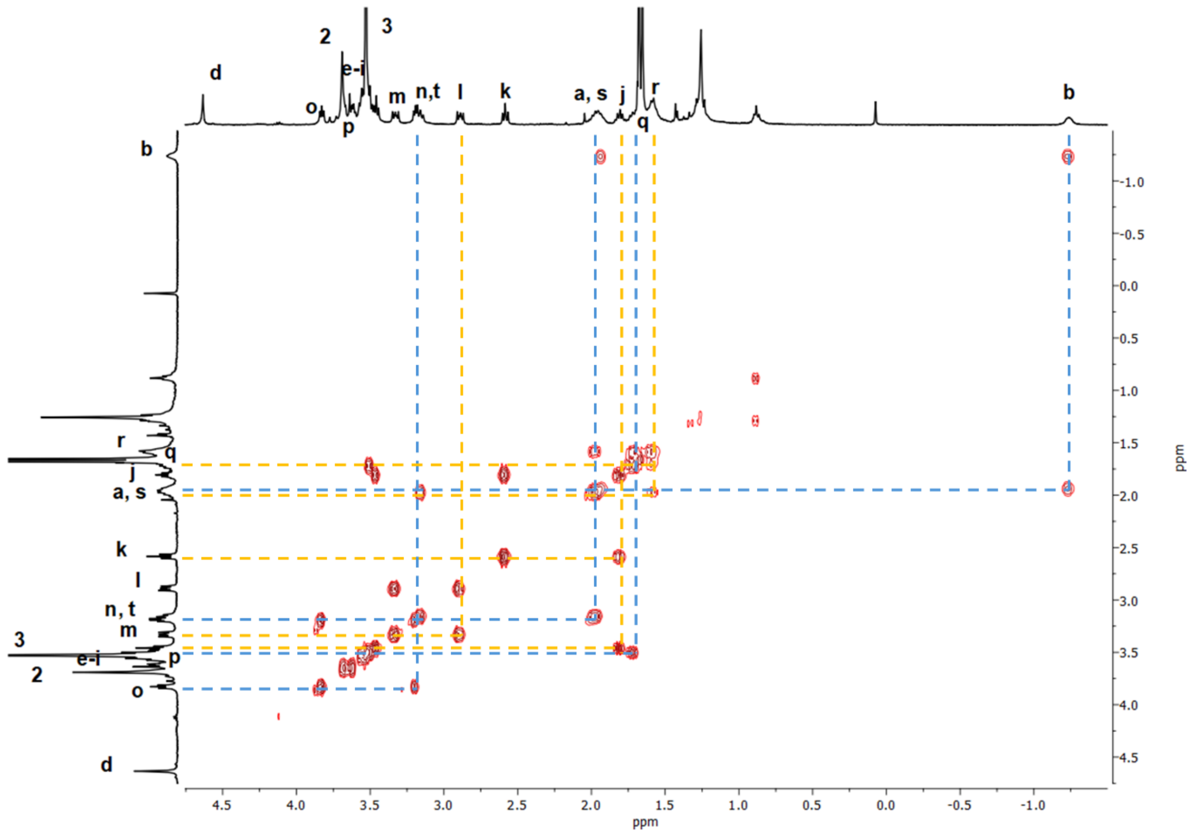

Figure S60. Partial COSY NMR (500 MHz, $CDCl_3$) spectrum of compound **4**.

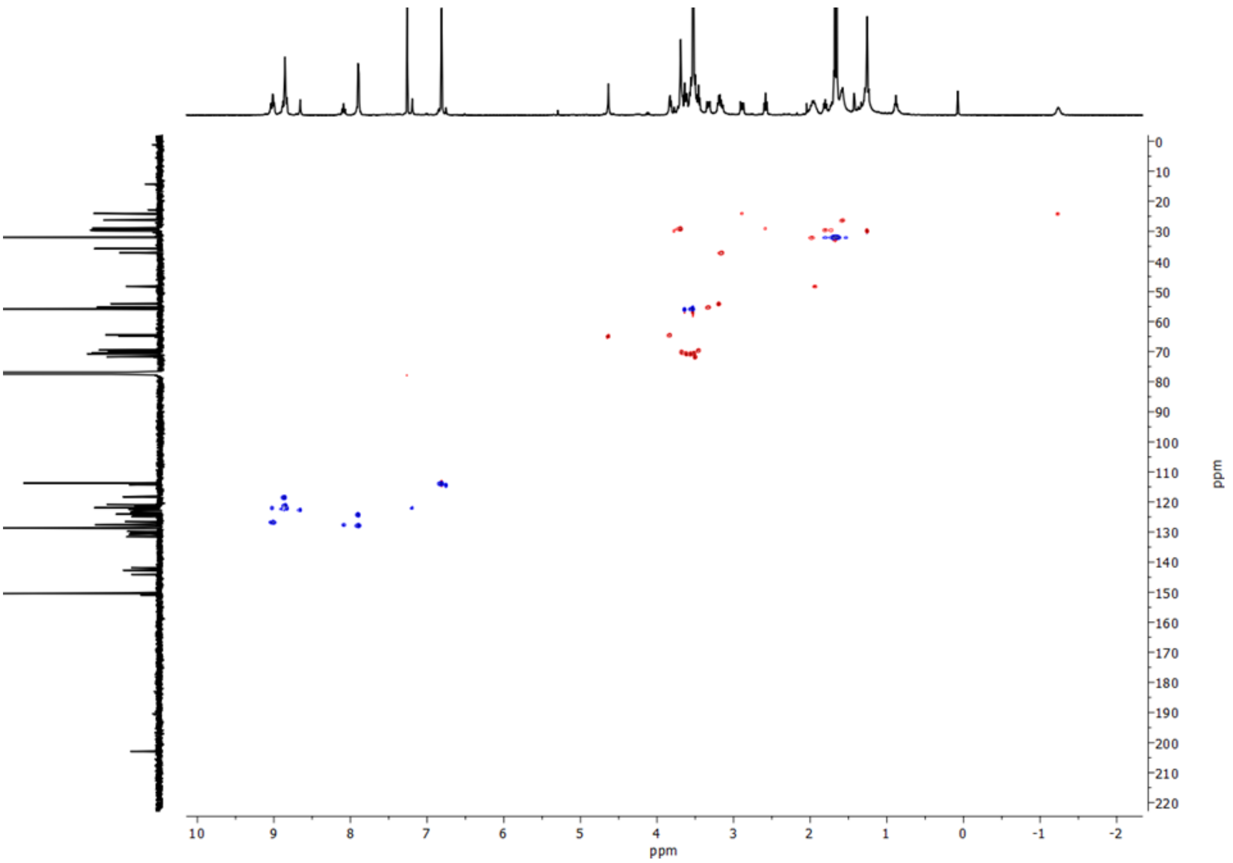

Figure S61. HSQC NMR (500, 126 MHz, $CDCl_3$) spectrum of compound **4**.

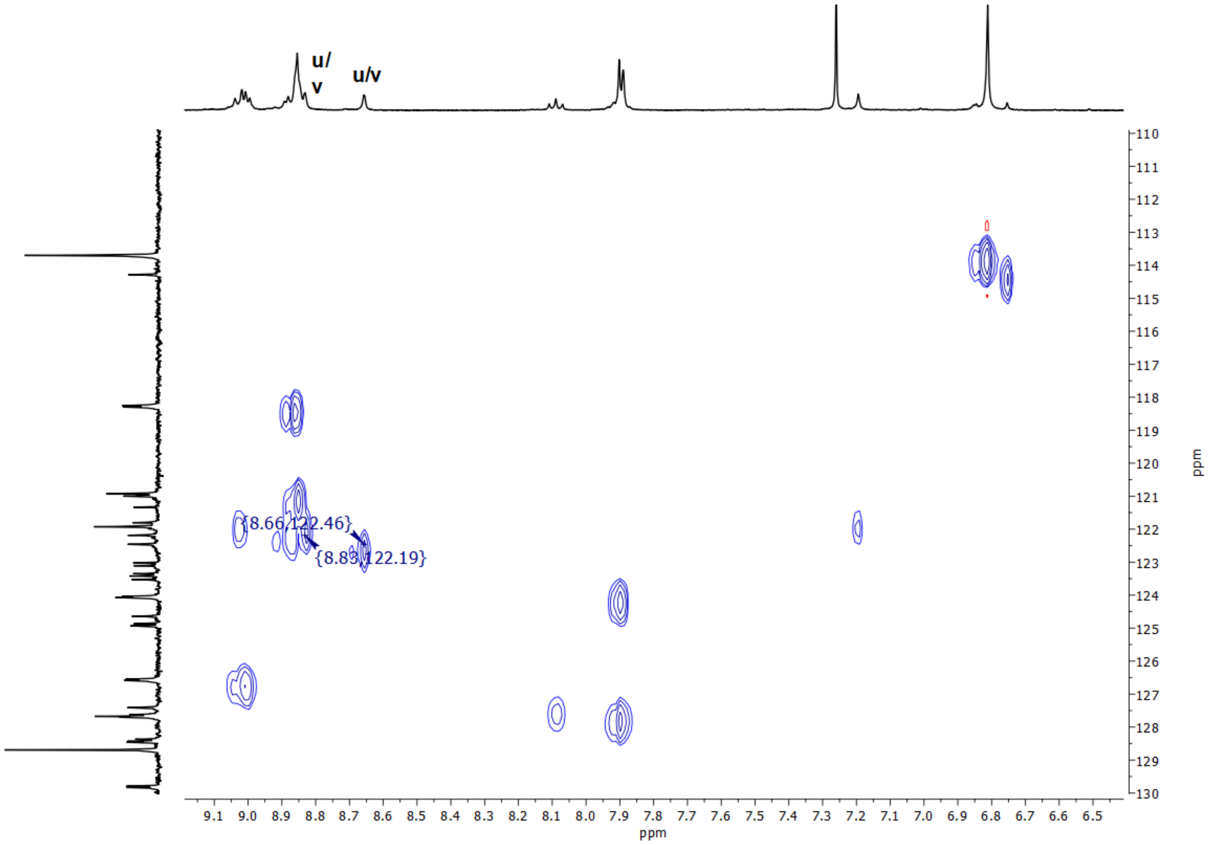

Figure S62. Partial HSQC NMR (500, 126 MHz, $CDCl_3$) spectrum of compound **4**.

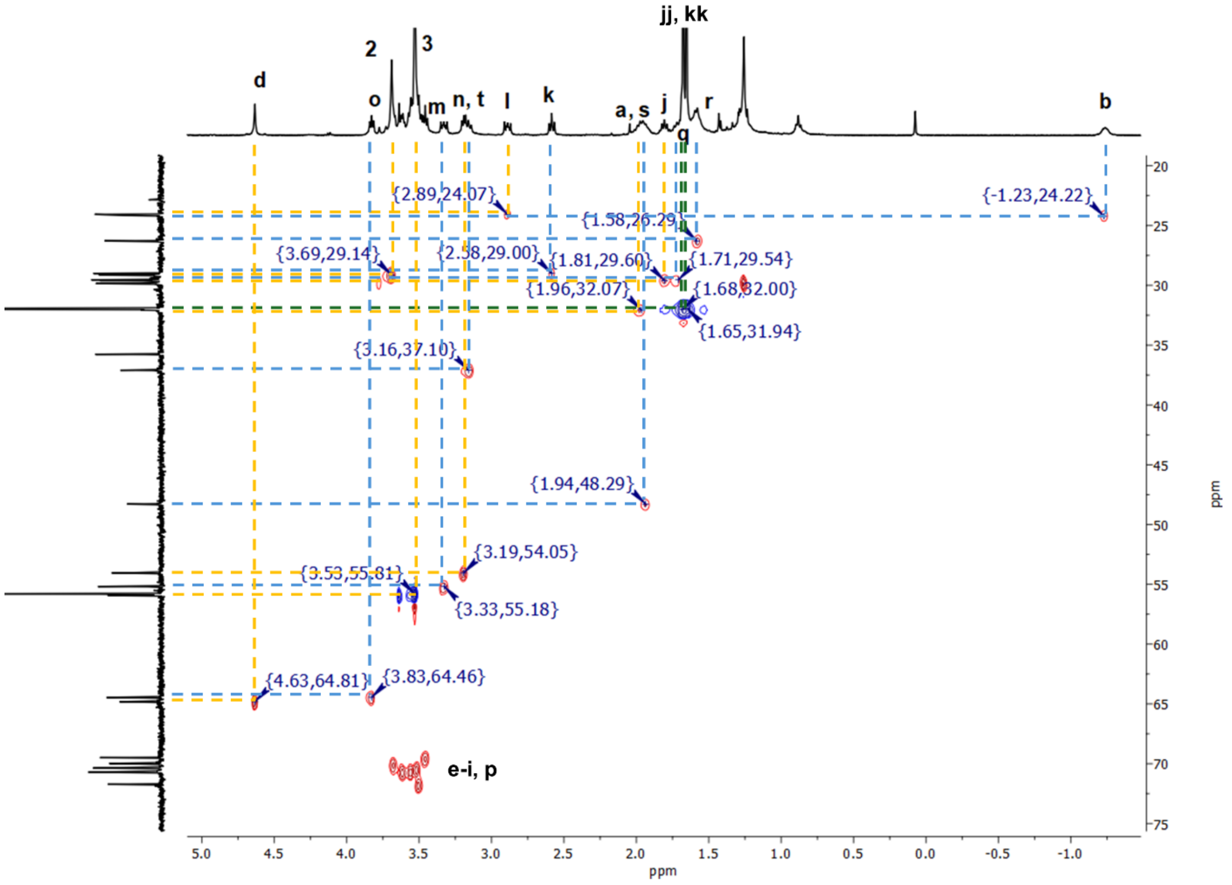

Figure S63. Partial HSQC NMR (500, 126 MHz, $CDCl_3$) spectrum of compound **4**.

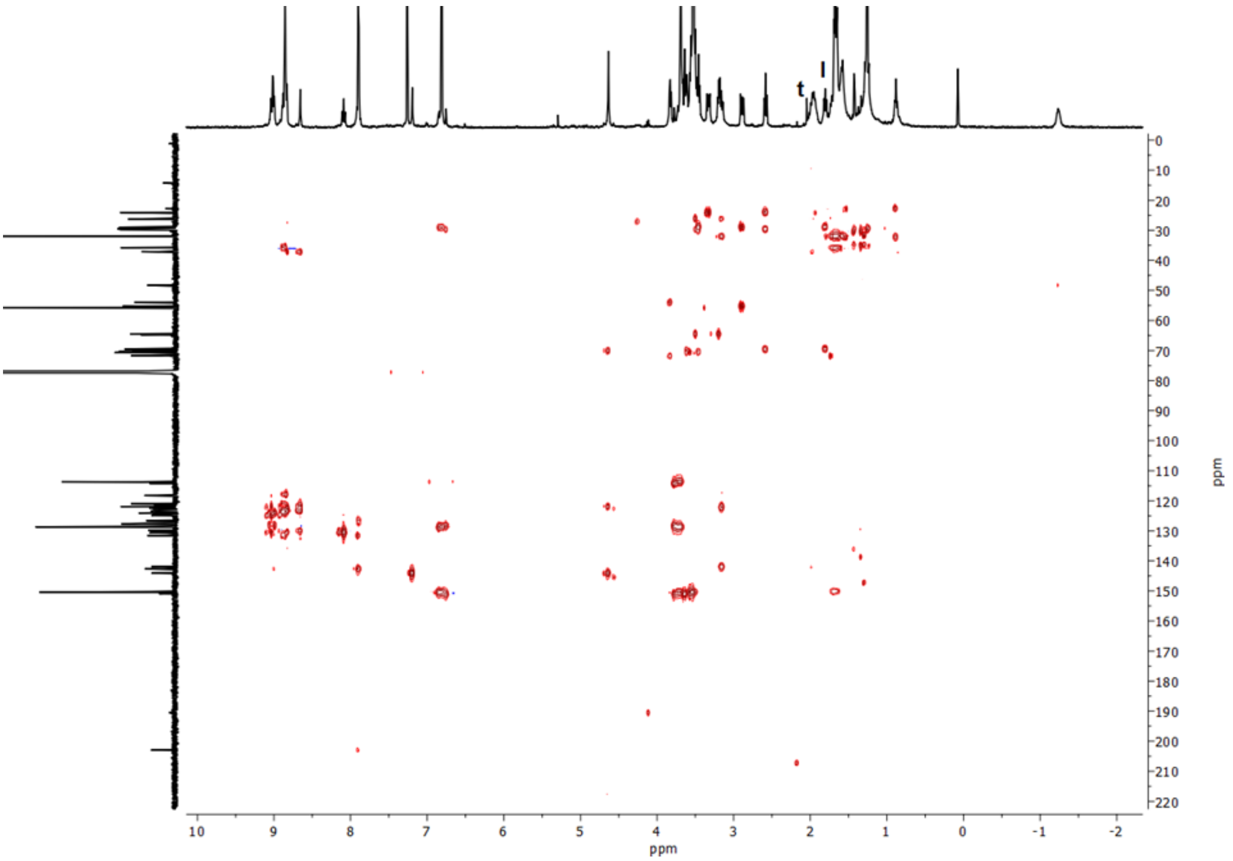

Figure S64. HMBC NMR (500 and 126 MHz, $CDCl_3$) spectrum of compound **4**.

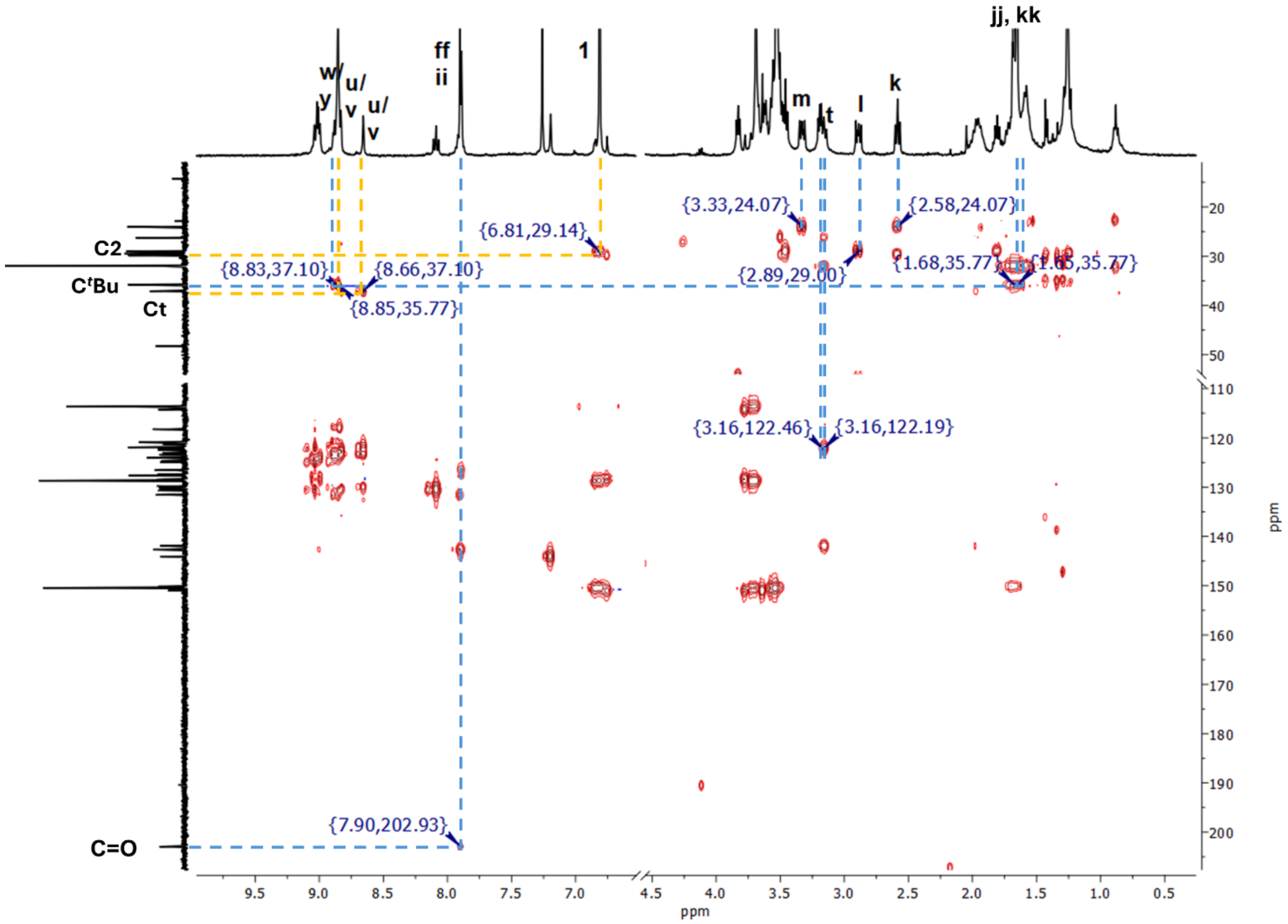

Figure S65. Partial HMBC NMR (500 and 126 MHz, $CDCl_3$) spectrum of compound **4**.

2.3. ^{1}H NMR spectra of known compounds

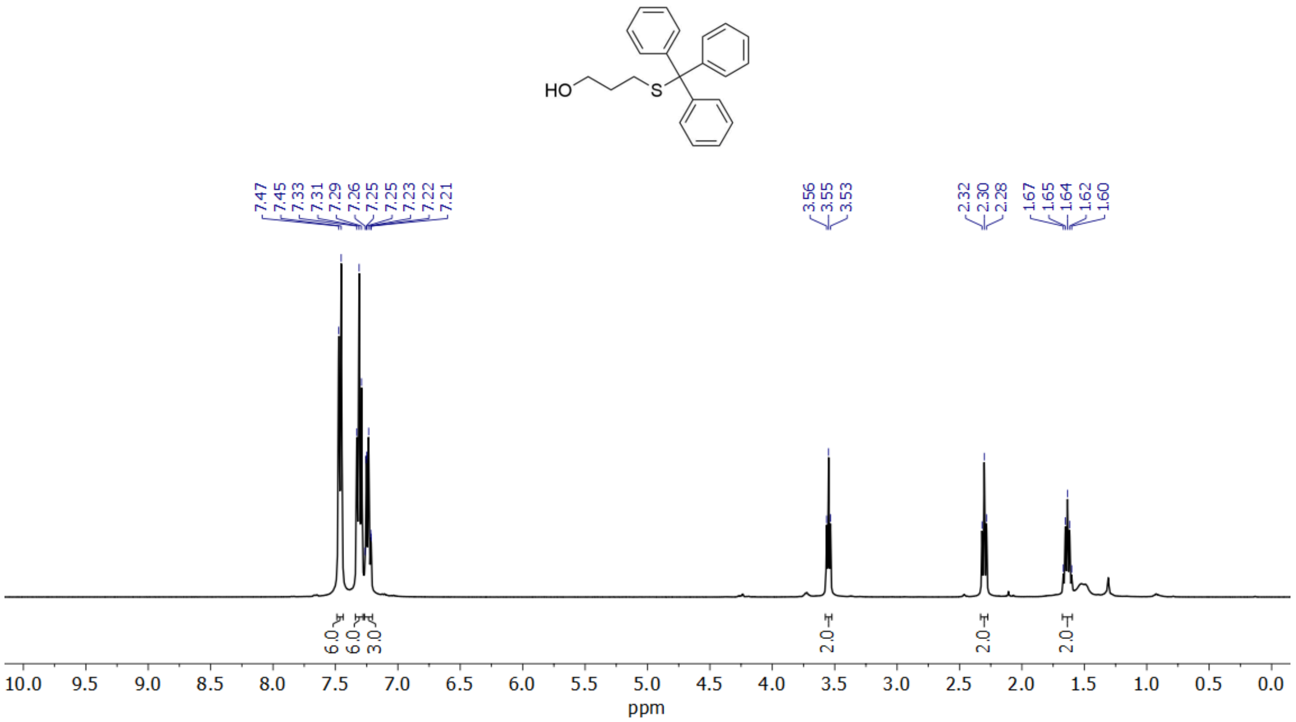

Figure S66. ^{1}H NMR (400 MHz, $CDCl_3$) spectrum of compound **13**.

3. MALDI-HRMS spectra

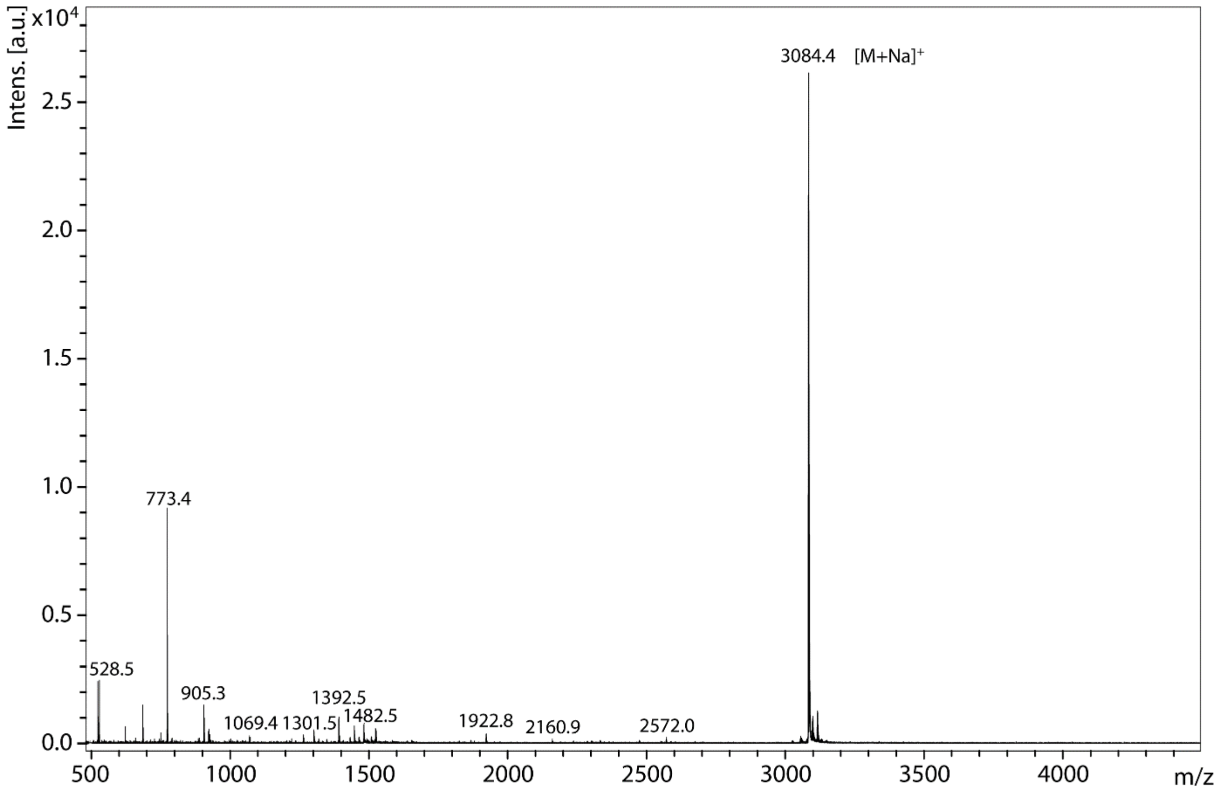

Figure S67. Mass Spectrometry (MALDI-TOF$^+$) spectrum of rotaxane **4**.

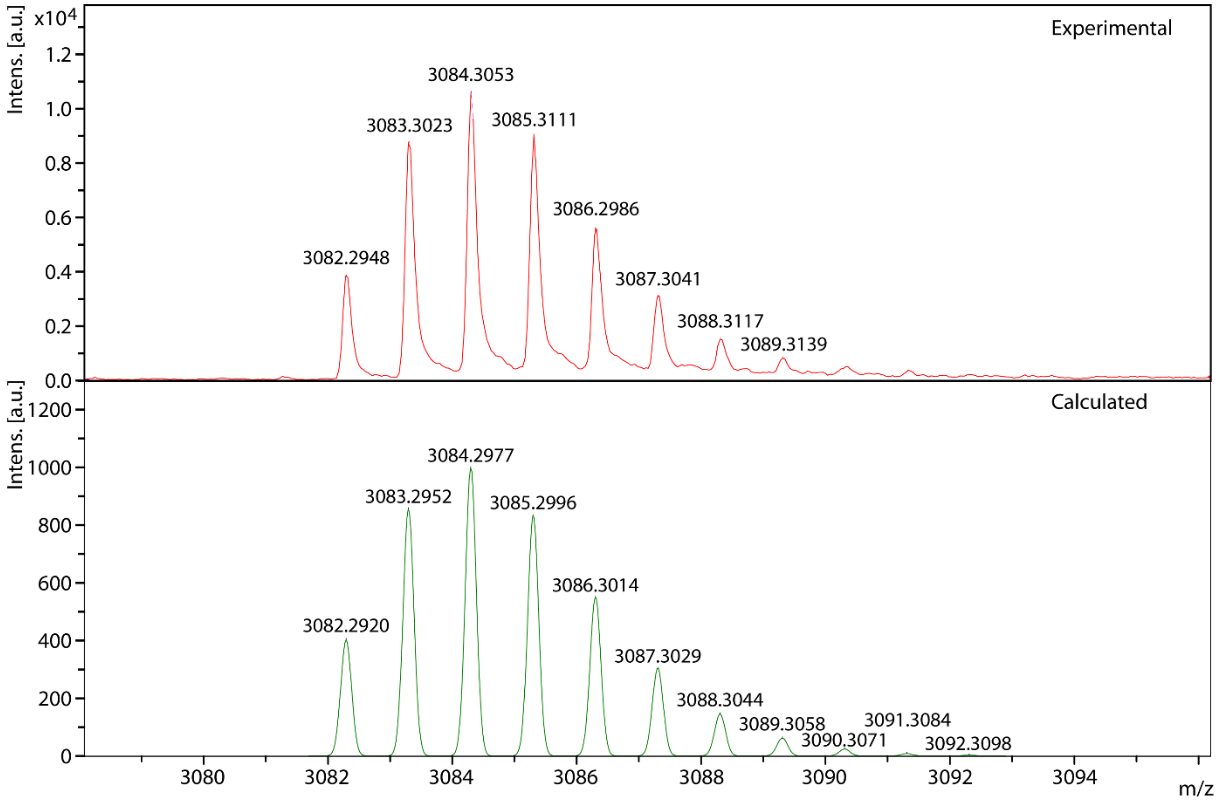

Figure S68. Experimental (top) and theoretical (bottom) HRMS isotopic distribution for the [M+Na]$^+$ ion ($C_{189}H_{194}N_6NaO_{24}S_4$) of **4**.

4. Photophysical properties

The UV-vis absorption spectra were recorded with a JASCO V-540 spectrophotometer. The photoluminescence spectra were performed on a Horiba Jobin Yvon Fluorolog 3-22 spectrofluorimeter equipped with a 450 W xenon lamp. The fluorescence quantum yields were measured upon excitation using Coumarin 153 in dimethyl sulfoxide (DMSO) as standard (fluorescence quantum yield of 0.465, λ_{em} = 500-600 nm). Several solutions were prepared in the 0.3-0.7 μM concentration range. To avoid non-linear effects, the absorbances in the 5 mm quartz cuvettes were keep bellow 0.06 at the excitation wavelength (377 nm). The fluorescence spectra were measured for each sample and standard at the same conditions. The slopes (m) obtained from the linear fit were used to calculate the emission quantum yield (ϕ) according to eq. 1:

$$\phi = \phi_s \frac{m}{m_s} \frac{n^2}{n_s^2} \qquad \text{(eq. 1)}$$

where the subscript *s* denoted the standard, *m* is the slope from the plot of integrated fluorescence intensity versus the absorbance, and *n* the refractive index of the solvent.

The emission lifetimes were measured by the Single-Photon Timing technique in a home-built setup using a linear excitation source operating at 340 nm with a 4 MHz repetition rate (second harmonic of a Coherent Radiation Dye laser 700 series, 610-680 nm, 130 mW, 5 ps, 4 MHz), an Hamamatsu R2809U-01 MCP-PMT (290−700 nm) as the detector and an SPC-160 photon counting board from Becker & Hickl GmbH. The emission at 500 nm was collected at the magic angle. The instrument response functions (IRF) for deconvolution were generated by scattering dispersions of colloidal silica in water. The solution was kept under gentle stirring during the data collection. Blank decays were acquired to ensure that dark photon counts were negligible. Decay curves were stored in 1024 channels with 24.4 ps per channel and an accumulation of 20k counts in the peak channel. The fluorescence decays were analyzed by a non-linear least-squares reconvolution method using the TRFA DP software by SSTC (Scientific Software Technologies Center, Belarusian State University, Minsk, Belarus).

The two-photon absorption spectrum was measured by two-photon excited fluorescence using Coumarin 153 in DMSO and in toluene as a standard to account for collection efficiency and pulse characteristics.[S8] A modified setup that follows closely the one described by Xu and Webb[S9] was used. To select a narrow bandwidth of emission wavelengths an H20Vis Jobin Yvon monochromator was placed at the entrance of a PMC-100-4 photomultiplyer tube (Becker and Hickl GmbH). The integrated intensity over the entire emission band was extrapolated using the emission spectra corrected by the detector sensitivity. The excitation source was a Ti:Sapphire laser (Tsunami BB, Spectra-Physics, 710-990 nm, 1.7 W, 100 fs, 82 MHz). In general, solutions of *ca.* 10-50 μM concentration in dichloromethane were used in the characterization of the non-linear properties.

The two-photon absorption cross-section was calculated from eq. 2:

$$\sigma_2 = \left(\frac{F_2}{\phi C n}\right)_{rot} \left(\frac{\phi C n \sigma_2}{F_2}\right)_s \qquad \text{(eq. 2)}$$

where F_2 stands for two-photon induced fluorescence intensity, ϕ is the one-photon excited fluorescence quantum yield, *n* refers to the refractive index in solution, *C* is the concentration and *rot* and *s* are relative to the rotaxane and the TPA standard, respectively. The emission intensity dependence of the excitation power was checked to be quadratic at 735 nm for all the compounds.

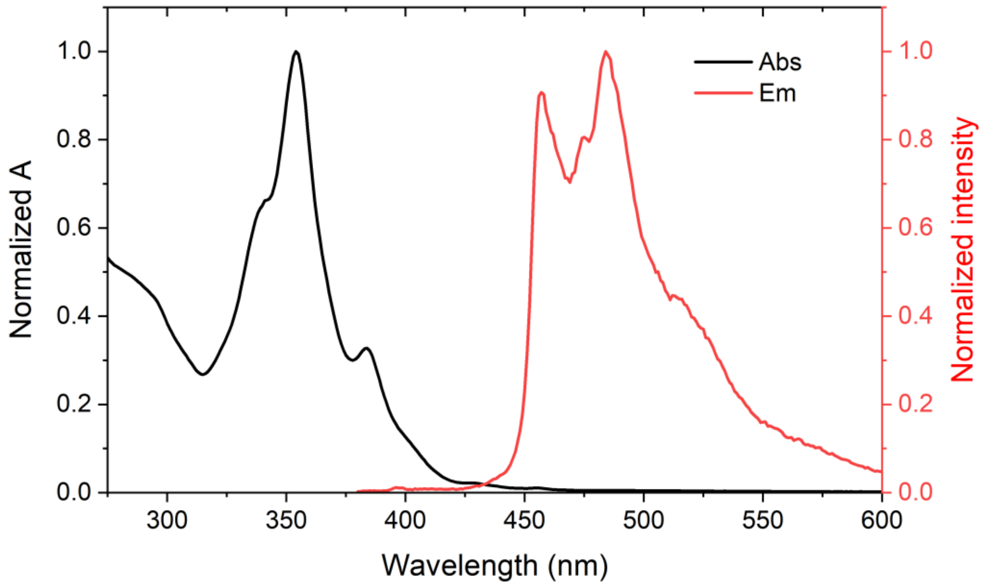

Figure S69. Normalized linear absorption (black line) and fluorescence (red line, λ_{exc} = 354 nm) spectra of rotaxane **4** in CH_2Cl_2 at 15 μM concentration.

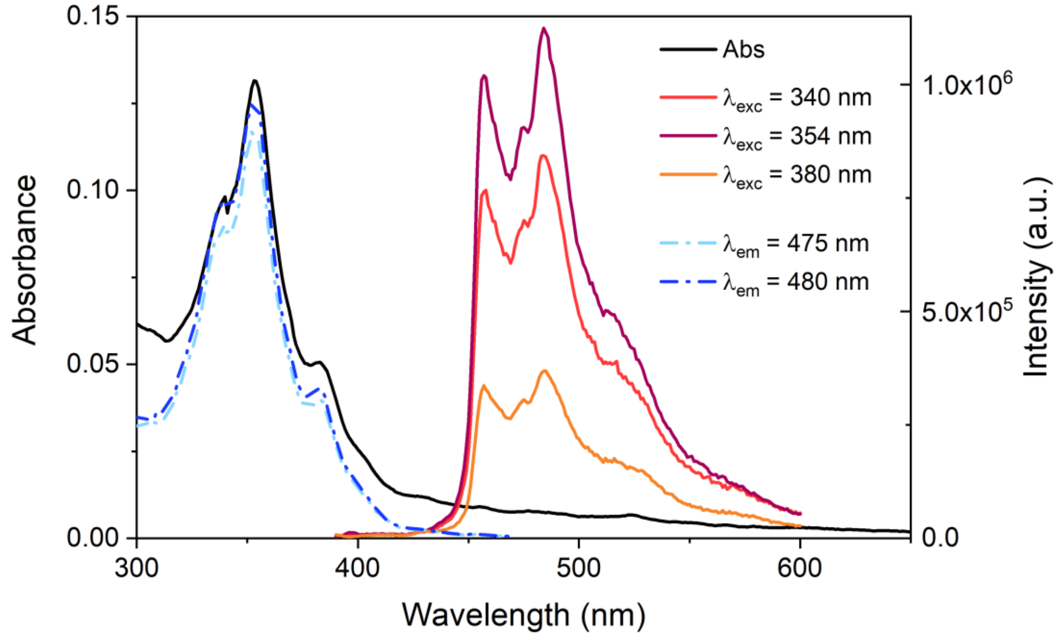

Figure S70. Linear absorption (black line), excitation (blue dashed lines) and emission (orange, red and dark red lines) spectra of rotaxane **4** (CH_2Cl_2, 1.5 μM) at different λ_{em} or λ_{exc}, respectively.

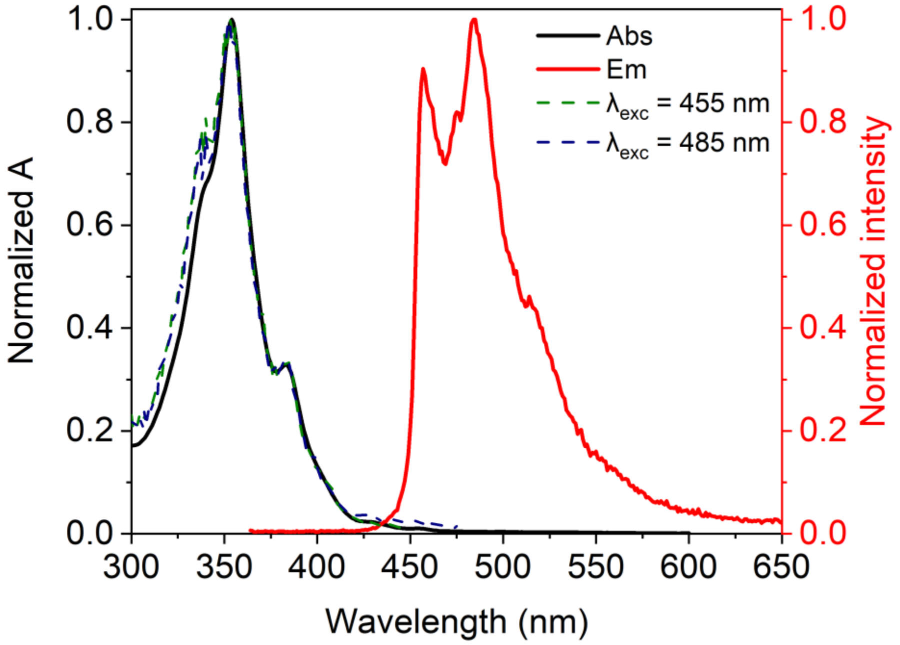

Figure S71. Normalized linear absorption (black line), excitation (blue and green dashed lines) and fluorescence (red line, λ_{exc} = 354 nm) spectra of thread **5** in CH_2Cl_2 at 10 μM concentration.

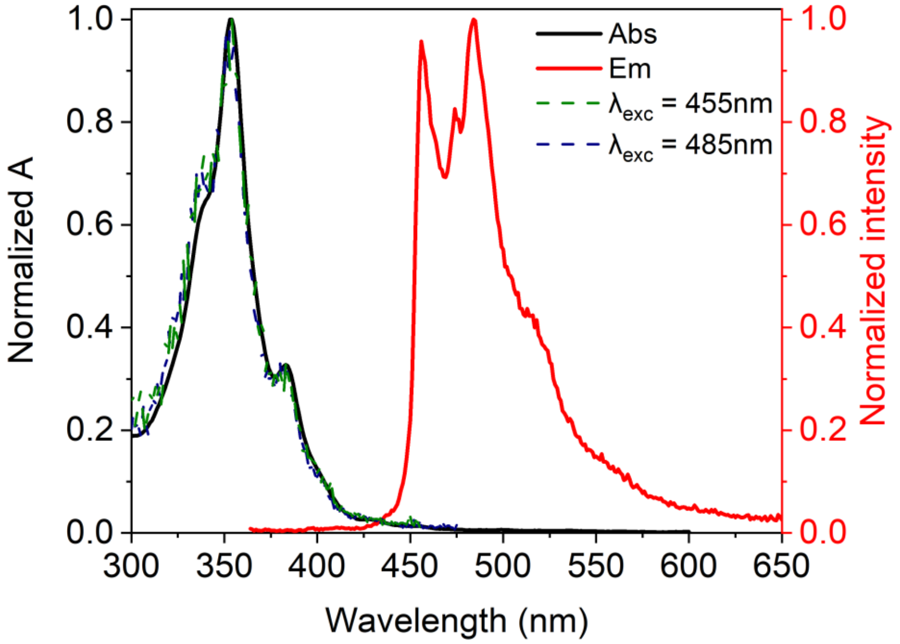

Figure S72. Normalized linear absorption (black line), excitation (blue and green dashed lines) and fluorescence (red line, λ_{exc} = 354 nm) spectra of **11** in CH_2Cl_2 at 10 μM concentration.

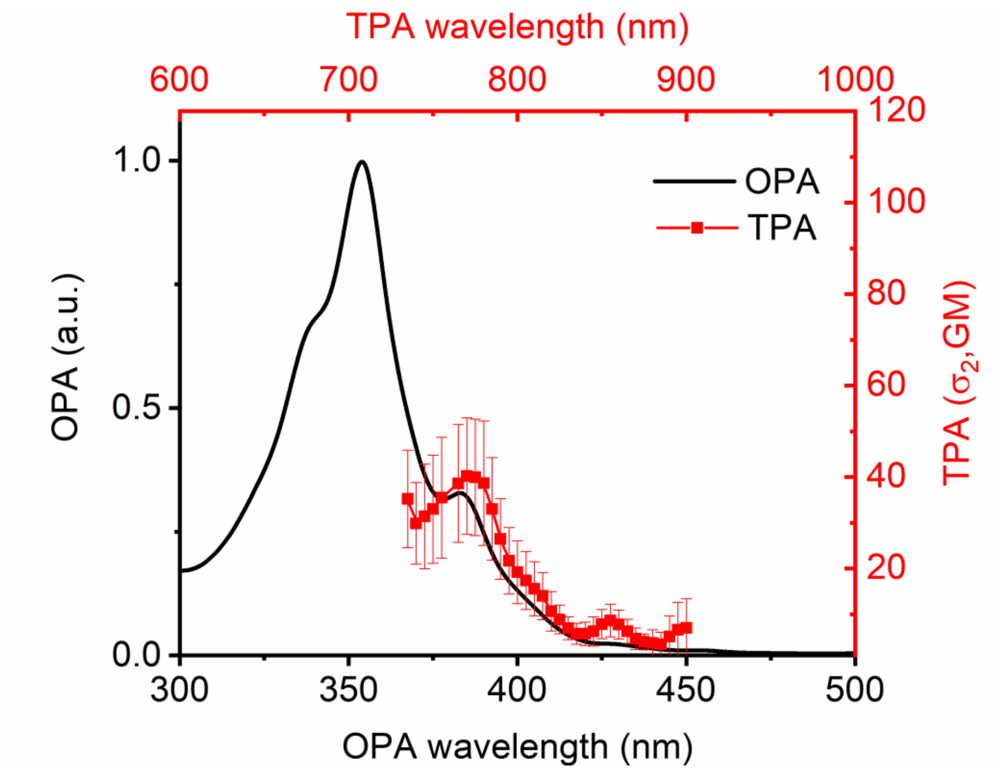

Figure S73. Normalized linear absorption (OPA, black line) and nonlinear two-photon absorption (TPA, red line) spectra of thread **5** in CH_2Cl_2 at 10 μM and 50 μM concentration, respectively.

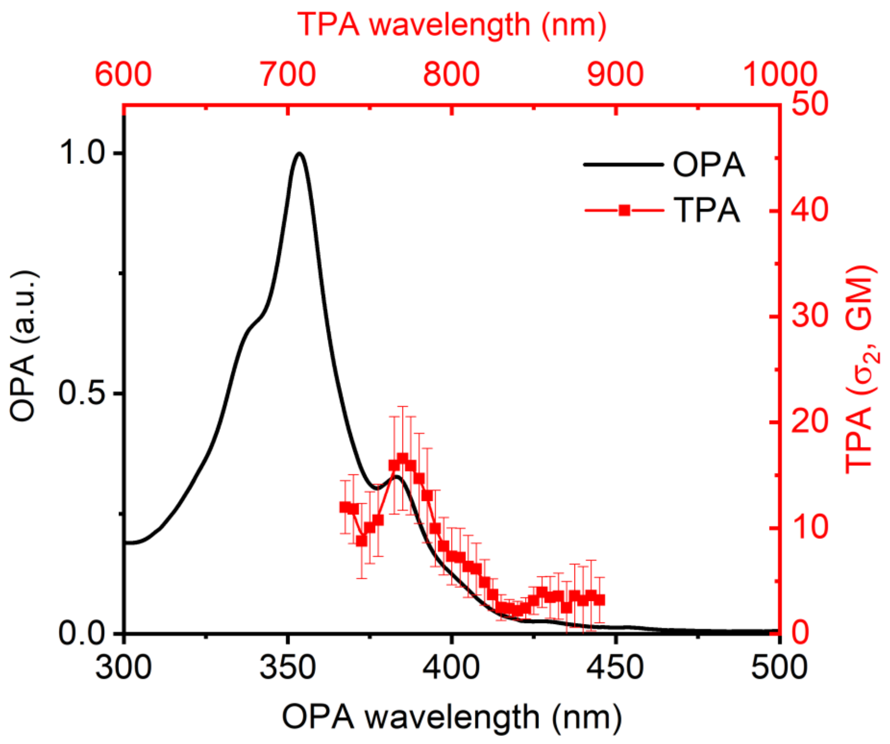

Figure S74. Normalized linear absorption (OPA, black line) and two-photon absorption (TPA, red line) spectra of *hept*-HBC derivative **11** in CH_2Cl_2 at 10 μM and 49 μM concentration, respectively.

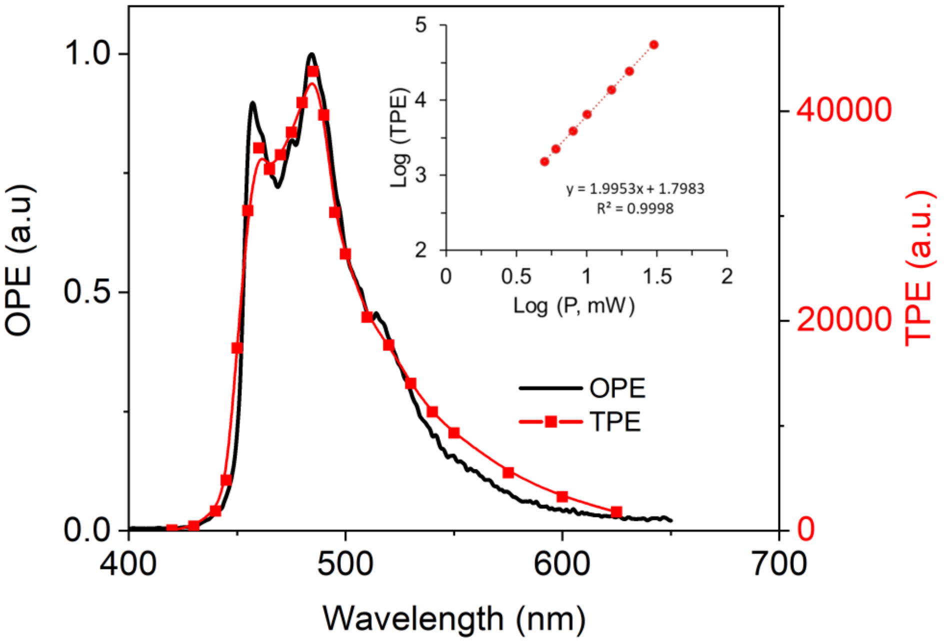

Figure S75. One-photon induced emission (OPE, black line, λ_{exc} = 354 nm) and two-photon induced emission (TPE, red line, λ_{exc} = 785 nm) spectra of thread **5** in CH_2Cl_2 at 10 μM and 50 μM concentration, respectively. Inset: log–log plot of the TPE intensity against the power of the excitation source at 735 nm.

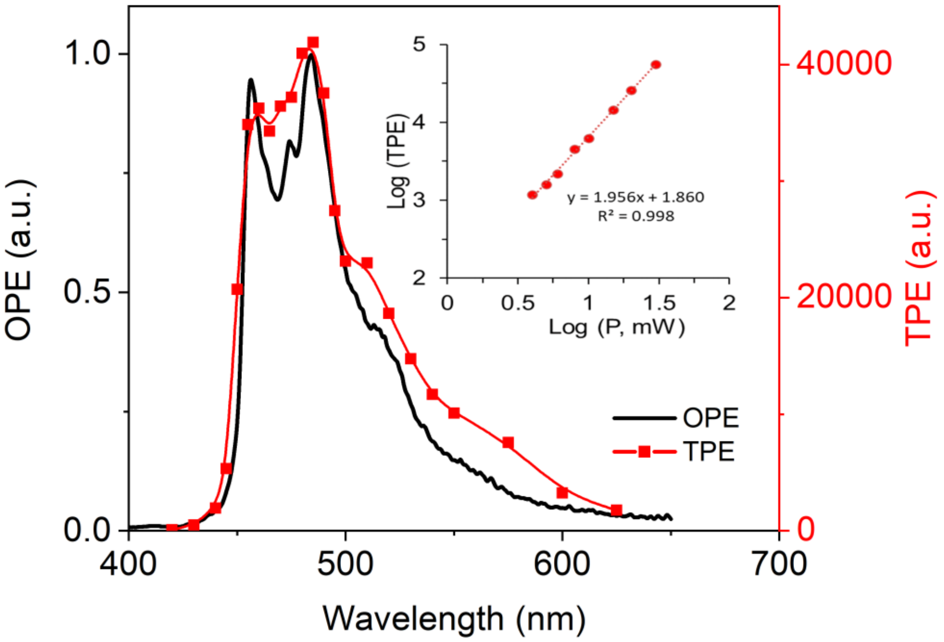

Figure S76. One-photon induced emission (OPE, black line, λ_{exc} = 354 nm) and two-photon induced emission (TPE, red line, λ_{exc} = 785 nm) spectra of compound **11** in CH_2Cl_2 at 10 μM and 49 μM concentration, respectively. Inset: log–log plot of the TPE intensity against the power of the excitation source at 735 nm.

Table S1. Summary of the photophysical properties of rotaxane **4**, thread **5** and *hept*-HBC derivative **11**.

Compound	ε ($M^{-1}cm^{-1}$)[a]	$\lambda_{Abs\ max}$ (nm)	$\lambda_{Em\ max}$ (nm)	Φ	Fluorescence Lifetime (ns)	TPA (GM)
hept-HBC **11**	7.73×10^4	354	484	0.012	3.7	17 ± 5 (770 nm)
Thread **5**	1.17×10^5	354	485	0.0073	3.6	40 ± 12 (770 nm)
Rotaxane **4**	1.68×10^5	354	484	0.0107	3.6	38 ± 9 (770 nm)

[a] Molar linear absorptivity coefficient measured at $\lambda_{Abs\ max}$ using a 10 μM solution in CH_2Cl_2.

5. Computational methods

Theoretical calculations were carried out with the Gaussian 09 software package.[S10] The optimization of a model structure (**S1**, Figures S77-S78) of the *hept*-HBC core in rotaxane **4** was carried out using the range-separated functional ωB97XD, which includes empirical dispersion,[S11] and the split-valence basis set def2-SVP.[S12] The structure obtained was studied by frequencies analysis to demonstrate it corresponds to a stationary point.

TD-DFT calculations were carried out with the long-range-corrected CAM-B3LYP[S13] functional and the triple-zeta def2-TZVP[S12] basis set using the coordinates obtained from the optimization of the structure at the ωB97XD/def2-SVP level of theory.

All calculations were carried out in CH_2Cl_2 using the Polarizable Continuum Model (PCM) with the integral equation formalism variant (IEFPCM) available in Gaussian 09.

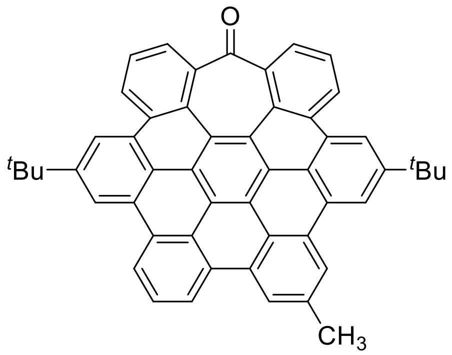

S1

Figure S77. Chemical structure of *hept*-HBC model **S1**.

5.1. Geometry optimization

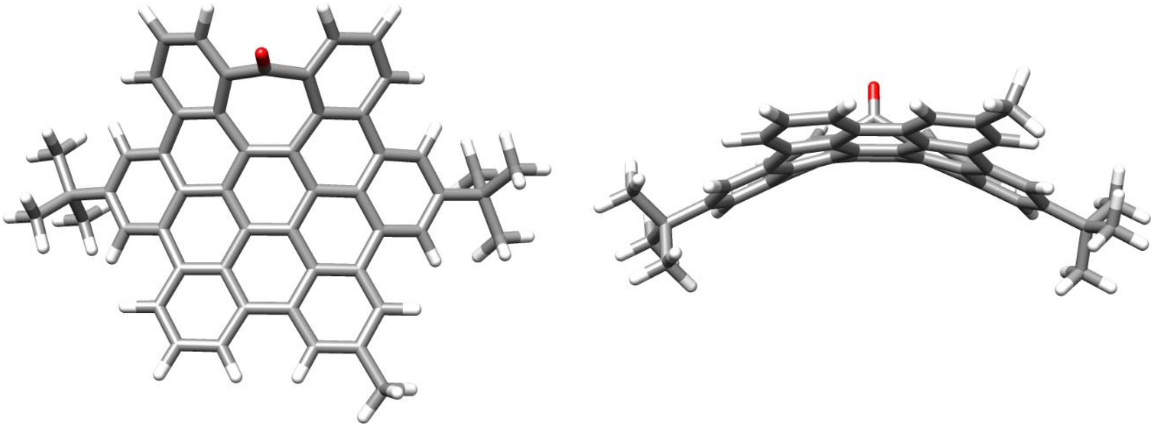

Figure S78. Top (left) and front (right) views of the optimized geometry (ωB97XD/def2-SVP) of **S1**.

Table S2. Cartesian coordinates of the optimized structure (ωB97XD/def2-SVP) of **S1**.

Atom	X	Y	Z	Atom	X	Y	Z
C	2.941456	-5.003715	-0.441966	C	2.865403	2.258320	-0.446960
C	3.398642	-3.834826	0.137217	C	3.631172	3.395697	-0.710587
C	2.669442	-2.634448	0.068488	C	3.046355	4.586436	-1.145453
C	1.366969	-2.624415	-0.502487	C	1.659138	4.636279	-1.263244
C	0.972137	-3.810828	-1.160394	C	0.851602	3.520687	-1.006870
C	1.746245	-4.969360	-1.148841	C	-0.612322	3.602233	-1.019537
C	0.564397	-1.377850	-0.486933	C	-1.287678	4.799738	-1.293349
C	-0.872171	-1.300114	-0.498367	C	-2.669839	4.879638	-1.192162
C	-1.803628	-2.452199	-0.526316	C	-3.404671	3.776973	-0.771889
C	-1.528526	-3.676100	-1.177048	C	-2.767602	2.564542	-0.491892
C	-0.283451	-3.936272	-1.935756	C	-3.495868	1.400708	0.017652
C	1.308199	-0.171917	-0.369233	C	-4.764555	1.531263	0.581906
C	0.679134	1.088890	-0.523914	C	-5.469789	0.439887	1.098292
C	-0.720307	1.165364	-0.534433	C	-4.888354	-0.817409	0.962556
C	-1.483668	-0.020324	-0.391245	C	-3.631251	-0.997447	0.368792
C	-3.108551	-2.320573	0.024450	C	-6.832143	0.661681	1.768837
C	-3.961743	-3.437210	0.086205	C	6.659477	-0.150822	1.890183
C	-3.623484	-4.649829	-0.484092	O	-0.301788	-4.416107	-3.049199
C	-2.421897	-4.745057	-1.174947	C	-7.458170	-0.651681	2.254307
C	2.710675	-0.176593	0.007868	C	7.290618	1.245812	1.951575
C	1.467320	2.296034	-0.654080	C	7.616180	-1.082571	1.123001
C	-1.369496	2.453757	-0.676135	C	6.478078	-0.666369	3.329824
C	-2.884041	0.124316	-0.036278	C	-7.794477	1.314608	0.759368
C	3.325674	-1.375101	0.426819	C	-6.647329	1.590389	2.983461
C	4.579269	-1.327576	1.040697	H	3.536347	-5.917067	-0.392174
C	5.295354	-0.137468	1.187128	H	4.381328	-3.842587	0.605844
C	4.725291	1.019770	0.660475	H	1.392213	-5.844835	-1.695369
C	3.454985	1.022950	0.072689	H	-4.945278	-3.339331	0.542489

Atom	X	Y	Z
H	-4.312471	-5.494804	-0.440345
H	-2.156398	-5.654859	-1.715485
H	5.022327	-2.248157	1.417852
H	5.261145	1.962716	0.741022
H	4.715550	3.361669	-0.592676
H	1.204498	5.582405	-1.557409
H	-0.734160	5.691540	-1.584747
H	-3.178765	5.816724	-1.425511
H	-4.487397	3.864518	-0.679443
H	-5.203534	2.526025	0.658882
H	-5.428162	-1.687281	1.326909
H	-6.824660	-1.156829	2.999356
H	-7.641346	-1.350392	1.423648
H	-8.427647	-0.443684	2.731193
H	7.472624	1.658068	0.947315
H	6.660648	1.955848	2.508853
H	8.261350	1.189342	2.466454
H	7.245095	-2.117767	1.091046
H	7.750872	-0.738591	0.085952
H	8.603795	-1.098003	1.609975
H	5.791873	-0.017315	3.895296
H	7.446555	-0.680374	3.853884
H	6.072632	-1.688890	3.351973
H	-7.931537	0.672137	-0.124039
H	-8.780436	1.474471	1.223060
H	-7.427569	2.292498	0.413896
H	-5.956732	1.145003	3.716015
H	-7.613906	1.760533	3.483065
H	-6.245007	2.571966	2.691999
C	3.899552	5.780807	-1.480349
H	4.735945	5.885252	-0.774392
H	4.332885	5.675668	-2.487886
H	3.315113	6.711000	-1.465111

0 imaginary frequencies
Zero-point correction = 0.728797 (Hartree/Particle)
Thermal correction to Energy = 0.768151
Thermal correction to Enthalpy = 0.769095
Thermal correction to Gibbs Free Energy = 0.658821
Sum of electronic and zero-point Energies = −2075.770103
Sum of electronic and thermal Energies = −2075.730750
Sum of electronic and thermal Enthalpies = −2075.729806
Sum of electronic and thermal Free Energies = −2075.840080

5.2. TD-DFT

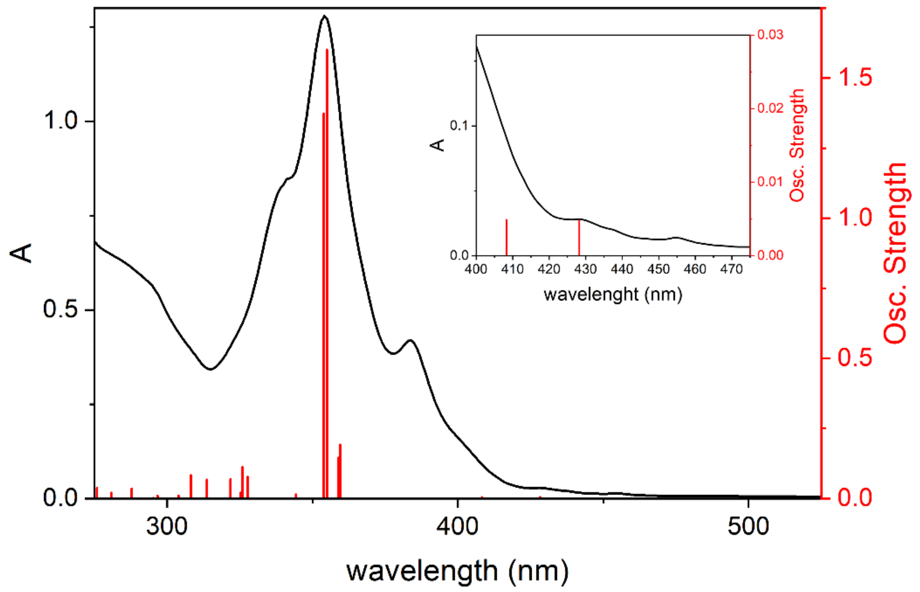

Figure S79. Experimental UV-vis spectrum of rotaxane **4** (10 µM in CH_2Cl_2, black line) and TD-DFT calculated (CAM-B3LYP/def2TZVP) electronic transitions (red lines) for model *hept*-HBC derivative **S1**. Correction = −0.45 eV.

Table S3. 50 lowest-energy calculated (CAM-B3LYP/def2-TZVP) electronic transitions of **S1**, including the predicted wavelength (nm) and their oscillator strength (cgs). The wavelengths shown result from the correction of the energy of each transition by −0.45 eV.

Transition number	Wavelength (nm)	Osc. Strength (cgs)	Transition number	Wavelength (nm)	Osc. Strength (cgs)
1	428.24	0.0048	18	280.73	0.0206
2	408.32	0.0049	19	275.86	0.0385
3	359.48	0.1918	20	274.25	0.0855
4	358.92	0.1454	21	272.87	0.0132
5	354.95	1.6017	22	269.17	0.0446
6	353.88	1.3739	23	266.94	0.0055
7	344.29	0.0143	24	265.16	0.002
8	327.68	0.0773	25	262.37	0.0158
9	325.81	0.1115	26	260.90	0.0196
10	325.23	0.0204	27	260.57	0.0036
11	321.76	0.0694	28	258.04	0.0054
12	313.72	0.0666	29	254.99	0.006
13	308.10	0.083	30	252.52	0.0062
14	303.96	0.0094	31	252.02	0.0597
15	296.71	0.0095	32	251.02	0.0522
16	295.10	0.0018	33	245.59	0.3725
17	287.82	0.0342	34	244.62	0.3322

35	242.83	0.0762	43	230.14	0.2022
36	240.70	0.0378	44	227.71	0.5981
37	238.37	0.4667	45	227.55	0.4128
38	236.75	0.2045	46	226.85	0.0948
39	236.50	0.0048	47	225.30	0.0535
40	234.59	1.1812	48	224.75	0.0664
41	233.39	0.0288	49	222.54	0.1724
42	231.16	0.0831	50	221.18	0.1441

6. References

S1 Ogoshi, T.; Kanai, S.; Fujinami, S.; Yamagishi, T.-A.; Nakamoto, Y. *Para*-Bridged Symmetrical Pillar[5]arenes: Their Lewis Acid Catalyzed Synthesis and Host–Guest Property. *J. Am. Chem. Soc*., **2008**, *130*, 5022-5023. DOI: https://doi.org/10.1021/ja711260m

S2 Márquez, I. R.; Fuentes, N.; Cruz, C. M.; Puente-Muñoz, V.; Sotorrios, L.; Marcos, M. L.; Choquesillo-Lazarte, D.; Biel, B.; Crovetto, L.; Gómez-Bengoa, E.; González, M. T.; Martin, R.; Cuerva J. M.; Campaña, A. G. Versatile synthesis and enlargement of functionalized distorted heptagon-containing nanographenes. *Chem. Sci.,* **2017**, *8*, 1068-1074. DOI: https://doi.org/10.1039/C6SC02895K

S3 Holmes, M.; Kwon, D.; Taron, M.; Britton, R. Total Synthesis of Amphirionin-4. *Org. Lett.*, **2015**, *17*, 3868-3871. DOI: https://doi.org/10.1021/acs.orglett.5b01844

S4 Diot, J.; García-Moreno, M. I.; Gouin, S. G.; Mellet, C. O.; Haupt, K.; Kovensky, J. Multivalent iminosugars to modulate affinity and selectivity for glycosidases. *Org. Biomol. Chem.*, **2009**, *7*, 357-363. DOI: https://doi.org/10.1039/B815408B

S5 Ju, Y.; Kumar, D.; Varma, R. S. Revisiting Nucleophilic Substitution Reactions: Microwave-Assisted Synthesis of Azides, Thiocyanates, and Sulfones in an Aqueous Medium. *J. Org. Chem*., **2006**, *71*, 6697-6700. DOI: https://doi.org/10.1021/jo061114h

S6 Connolly, B. A.; Rider, P. Chemical synthesis of oligonucleotides containing a free sulphydryl group and subsequent attachment of thiol specific probes. *Nucleic Acids Res.,* **1985**, *13*, 4485-4502. DOI: https://doi.org/10.1093/nar/13.12.4485

S7 Tormena, C. F.; Evans, R.; Haiber, S.; Nilsson, M.; Morris, G. A. Matrix-assisted diffusion-ordered spectroscopy: application of surfactant solutions to the resolution of isomer spectra. *Magn. Reson. Chem.,* **2012**, *50*, 458–465. DOI: https://doi.org/10.1002/mrc.3822

S8 Reguardati, S.; Pahapill, J.; Mikhailov, A.; Stepanenko, Y.; Rebane, A. High-accuracy reference standards for two-photon absorption in the 680–1050 nm wavelength range. *Opt. Express*, **2016**, *24*, 9053-9066. DOI: https://doi.org/10.1364/OE.24.009053

S9 Xu, C.; Webb, W. W. Measurement of two-photon excitation cross sections of molecular fluorophores with data from 690 to 1050 nm. *J. Opt. Soc. Am. B*, **1996**, *13*, 481-491. DOI: https://doi.org/10.1364/JOSAB.13.000481

S10 Frisch, M. J.; Trucks, G. W.; Schlegel, H. B.; Scuseria, G. E.; Robb, M. A.; Cheeseman, J. R.; Scalmani, G.; Barone, V.; Mennucci, B.; Petersson, G. A.; Nakatsuji, H.; Caricato, M.; Li, X.; Hratchian, H. P.; Izmaylov, A. F.; Bloino, J.; Zheng, G.; Sonnenberg, J. L.; Hada, M.; Ehara, M.; Toyota, K.; Fukuda, R.; Hasegawa, J.; Ishida, M.; Nakajima, T.; Honda, Y.; Kitao, O.; Nakai, H.; Vreven, T.; Montgomery Jr., J. A.; Peralta, J. E.; Ogliaro, F.; Bearpark, M.; Heyd, J. J.; Brothers, E.; Kudin, K. N.; Staroverov, V. N.; Kobayashi, R.; Normand, J.; Raghavachari, K.; Rendell, A.; Burant, J. C.; Iyengar, S. S.; Tomasi, J.; Cossi, M.; Rega, N.; Millam, J. M.; Klene, M.; Knox, J. E.; Cross, J. B.; Bakken, V.; Adamo, C.; Jaramillo, J.; Gomperts, R.; Stratmann, R. E.; Yazyev, O.; Austin, A. J.; Cammi, R.; Pomelli, C.; Ochterski, J. W.; Martin, R. L.; Morokuma, K.; Zakrzewski, V. G.; Voth, G. A.; Salvador, P.; Dannenberg, J. J.; Dapprich, S.; Daniels, A. D.; Farkas, O.; Foresman, J. B.; Ortiz, J. V.; Cioslowski, J.; Fox, D. J. Gaussian 09, Revision B.01. Gaussian Inc., Wallingford, **2010**.

S11 Chai, J.-D.; Head-Gordon, M. Long-range corrected hybrid density functionals with damped atom–atom dispersion corrections. *Phys. Chem. Chem. Phys.,* **2008**, *10*, 6615-6620. DOI: https://doi.org/10.1039/B810189B

S12 Weigend, F.; Ahlrichs, R. Balanced basis sets of split valence, triple zeta valence and quadruple zeta valence quality for H to Rn: Design and assessment of accuracy. *Phys. Chem. Chem. Phys.*, **2005**, *7*, 3297-3305. DOI: https://doi.org/10.1039/B508541A

S13 Yanai, T.; Tew, D.; Handy, N. A new hybrid exchange–correlation functional using the Coulomb-attenuating method (CAM-B3LYP). *Chem. Phys. Lett.*, **2004**, *393*, 51-57. DOI: https://doi.org/10.1016/j.cplett.2004.06.011
